# Deconstructing a Species-Complex: Geometric Morphometric and Molecular Analyses Define Species in the Western Rattlesnake (*Crotalus viridis*)

**DOI:** 10.1371/journal.pone.0146166

**Published:** 2016-01-27

**Authors:** Mark A. Davis, Marlis R. Douglas, Michael L. Collyer, Michael E. Douglas

**Affiliations:** 1 Illinois Natural History Survey, Prairie Research Institute, University of Illinois Urbana Champaign, Champaign, IL, 61820, United States of America; 2 Department of Biological Sciences, University of Arkansas, Fayetteville, AR, 72701, United States of America; 3 Department of Biology, Western Kentucky University, Bowling Green, KY, 42101, United States of America; The University of Texas Arlington, UNITED STATES

## Abstract

Morphological data are a conduit for the recognition and description of species, and their acquisition has recently been broadened by geometric morphometric (GM) approaches that co-join the collection of digital data with exploratory ‘big data’ analytics. We employed this approach to dissect the Western Rattlesnake (*Crotalus viridis*) species-complex in North America, currently partitioned by mitochondrial (mt)DNA analyses into eastern and western lineages (two and seven subspecies, respectively). The GM data (i.e., 33 dorsal and 50 lateral head landmarks) were gleaned from 2,824 individuals located in 10 museum collections. We also downloaded and concatenated sequences for six mtDNA genes from the NCBI GenBank database. GM analyses revealed significant head shape differences attributable to size and subspecies-designation (but not their interactions). Pairwise shape distances among subspecies were significantly greater than those derived from ancestral character states via squared-change parsimony, with the greatest differences separating those most closely related. This, in turn, suggests the potential for historic character displacement as a diversifying force in the complex. All subspecies, save one, were significantly differentiated in a Bayesian discriminant function analysis (DFA), regardless of whether our priors were uniform or informative (i.e., mtDNA data). Finally, shape differences among sister-clades were significantly greater than expected by chance alone under a Brownian model of evolution, promoting the hypothesis that selection rather than drift was the driving force in the evolution of the complex. Lastly, we combine head shape and mtDNA data so as to derived an integrative taxonomy that produced robust boundaries for six OTUs (operational taxonomic units) of the *C*. *viridis* complex. We suggest these boundaries are concomitant with species-status and subsequently provide a relevant nomenclature for its recognition and representation.

## Introduction

Morphometric and molecular data can each carry significant phylogenetic signal, yet molecular approaches have been in ascendancy for the past decade, due in large part to their contemporary status [1], and a recognized capacity for detecting gaps that separate biodiversity elements. Both approaches offer a unique perspective, yet their juxtaposition has developed but slowly [2], due largely to the preemptive application of molecular methods [3], as well as an implication of incongruence when morphometric and molecular data are juxtaposed [4–5]. We recognize the accessibility and relative ease of molecular methods may indeed be indisputable, yet we suggest that arguments with regard to their congruence are still open to question [6–7].

We also submit the premise that the elevated accuracy and statistical power accrued from a more contemporary geometric morphometric (GM) approach now promotes comparative (and synchronous) analyses with molecular data [8–10]. This improvement in analytical resolution is particularly acute for the derivation of head or skull shape, in that the composite nature of this structure is critical for feeding, mating, and territorial defense across numerous taxonomic groups [11–12]. As such, it can be interpreted as a phenotypic component central to both resource accrual and reproductive segregation, and thus represents a paradigm for mosaic evolution [13].

Our study group, the Western Rattlesnake Complex, presents a compelling context within which to explore the congruence of molecular and composite morphological data. Mitochondrial (mt)DNA strongly support the recognition of two divergent lineages, with strong implications of reciprocal monophyly among constituents of the complex [14–16]. In this context, three species (*C*. *cerberus*, *C*. *oreganus*, and *C*. *viridis*) have been putatively recognized [17], yet their differentiation is based solely upon a single character set, and consequently its capacity for delineation is not recognized by many institutions. Furthermore, taxonomic boundaries as provided by legacy data (predominately meristic, sparse morphometric, and venom constituents) are relatively non-discriminatory [18]. As a result, older taxonomic designations are adhered to. Given this, and two centuries of accumulated data, a reevaluation of the complex is not only past due but also imperative in that conservation priorities are derived from, and thus require, accurate taxonomies [19]. Ample verification is provided by the unrelenting nature of the Anthropocene, with impacts duly recorded on biodiversity in general [20] and snakes in particular [21].

Here we apply GM analyses to untangle the morphological diversity within the Western Rattlesnake complex, and to evaluate these data concomitantly with previously derived estimates of genetic differentiation, with an endpoint being an integrative and collaborative taxonomy. In this sense, mtDNA data imply the presence of distinct lineages, yet they cannot sustain species-status [22]. Previous morphological data have also been less diagnostic, thus arguing for the application of more contemporary morphometric approaches, as herein.

**Fig 1:**
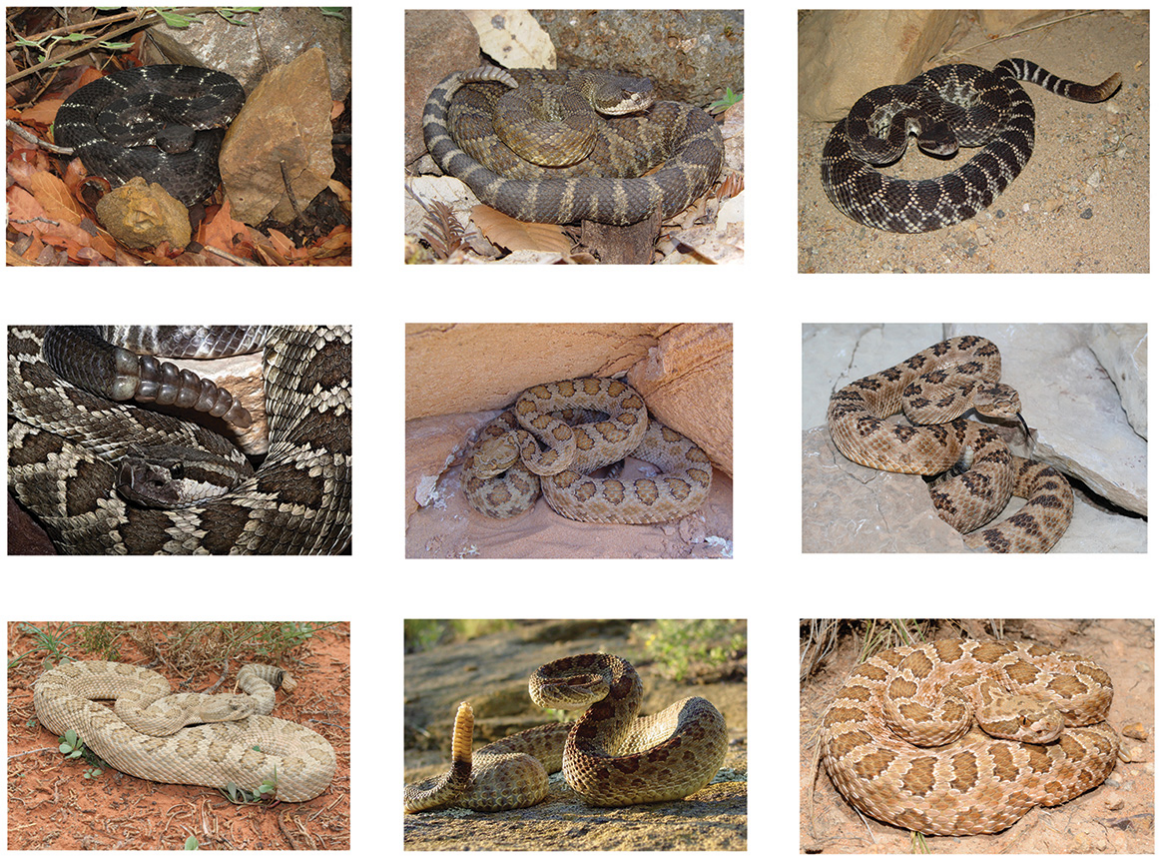
Operational Taxonomic Units in the Western Rattlesnake Complex. The Western Rattlesnake, historically composed of 9 subspecies (sensu Klauber), displays a level of phenotypic variability that is concomitant with its vast geographic range in North America. This variability is captured in the following images, identified to subspecies and location, and published with permission of the photographer (as indicated). Subspecies are as follows (from left to right, top to bottom): *Crotalus cerberus* (Pinal County AZ: Martin Feldner); *C*. *oreganus* (San Luis Obispo County CA: Martin Feldner); *C*. *o*. *helleri* (Los Angeles County CA: Martin Feldner); *C*. *o*. *caliginis* (San Coronado Island, Baja California: Rob Olivier); *C*. *o*. *concolor* (Coconino County AZ: Martin Feldner); C. *o*. *lutosus* (Mohave County AZ: Martin Feldner); *C*. *o*. *abyssus* (Coconino County AZ: Martin Feldner); *C*. *vviridis viridis* (Harding County SD: Mark Davis); *C*. *v*. *nuntius* (Coconino County AZ: William Wells).

In this context, we acquire geometric morphometric (GM) head shape data across the nine OTUs (operational taxonomic units) of the complex (Fig 1) and we apply multivariate analyses as a means to distinguish them in pairwise comparisons one from another. We also employed a diversification model driven by Brownian motion so as to compare the magnitude of morphological disparity within- versus among-lineages, and to test if disparity is greater among-lineages than expected by chance alone. Finally, we clarify the taxonomic status of the *C*. *viridis* OTUs by deriving an integrative taxonomy based upon our combined morphometric and molecular analyses. Our results suggest that six (of nine) OTUs in the Western Rattlesnake complex are indeed diagnosable as distinct species, and we offer a nomenclature to reflect these considerations.

## Materials and Methods

### Study Organism

**Fig 2:**
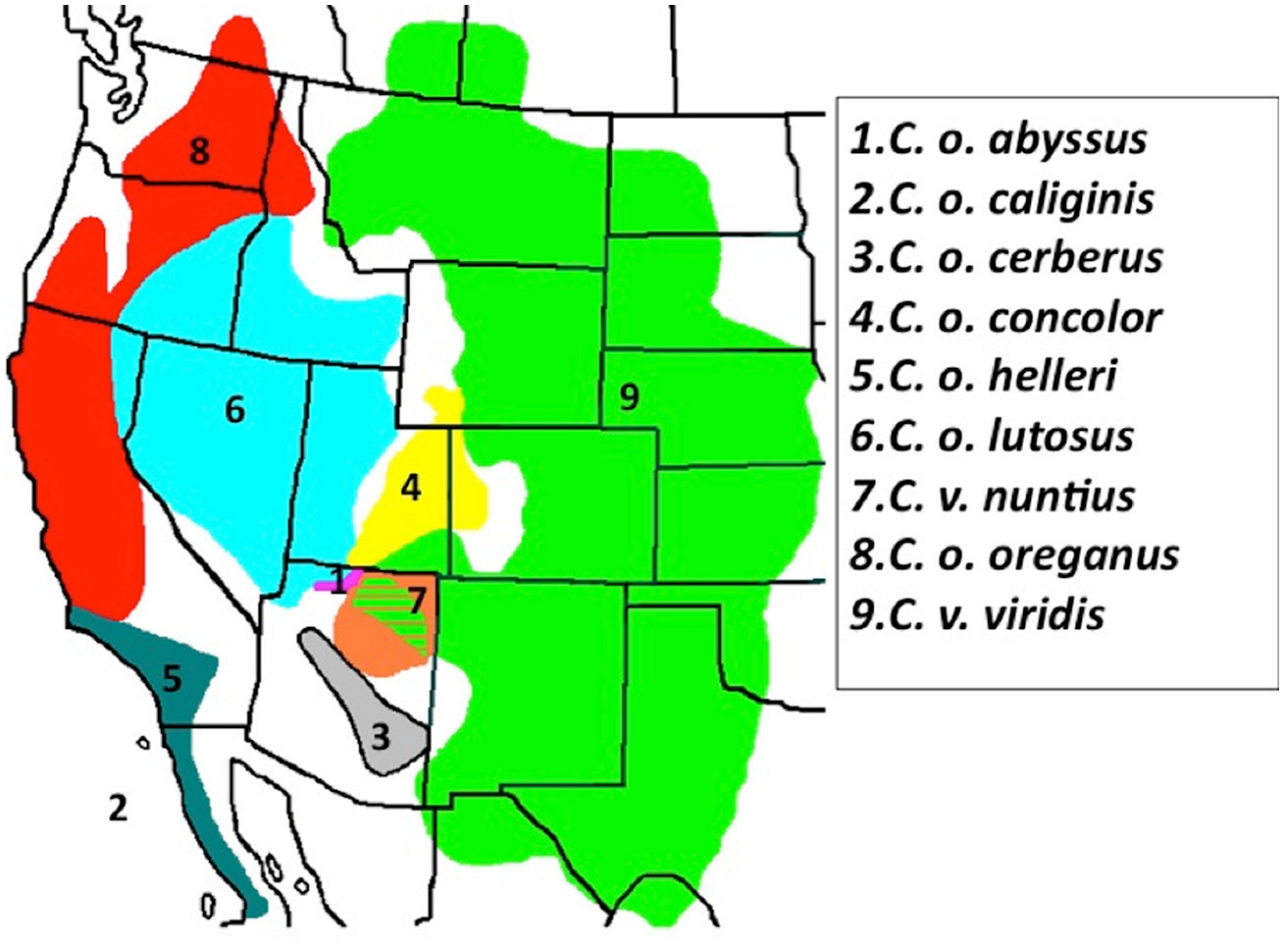
The topographic distribution of nine Western Rattlesnake OTUs (Operational Taxonomic Units) in North America. The nine Western Rattlesnake OTUs vary greatly in size and extent of distributional area. The Colorado Plateau represents a suture between eastern and western lineages, as six of nine occur there as either parapatric or sympatric.

The Western Rattlesnake (*Crotalus viridis*) is a polytypic North American pit viper [16, 23–24], widely distributed across broad latitudinal and elevational gradients. It extends from the Missouri River in the east, into Saskatchewan, Alberta, and British Columbia to the north, along the west coast of the United States, and south into Mexico (Fig 2; [23]). Habitat includes deciduous and coniferous forests, scrub, prairie grasslands, shrub steppe, desert margins, and sand dunes as arrayed across a gradient from sea level to 4000m [24]. As a consequence, it displays considerable variation within and among populations, life stages, and subspecies.

The traditionally accepted taxonomy is a single, polytypic complex (*Crotalus viridis*) defined largely via geography, meristics, and an assumption of intergradation. It is composed of nine OTUs, and the sample sizes for our GM analyses are indicated in parentheses following each taxonomic designation: *C*. *v*. *abyssus* (N = 31); *caliginis* (N = 33); *cerberus* (N = 163); *concolor* (N = 144); *helleri* (N = 446); *lutosus* (N = 515); *nuntius* (N = 256); *oreganus* (N = 590); *viridis* (N = 646). Molecular data suggests the presence of three species: *C*. *viridis* (to include *viridis* and *nuntius*); *C*. *oreganus* (to include *abyssus*, *caliginis*, *concolor*, *helleri*, *lutosus*, *oreganus*); and *C*. *cerberus* [17].

### Acquisition of Shape Data

High-resolution digital photographs of the dorsal and lateral head were obtained from 3,170 adult, juvenile, and neonate museum specimens of Western Rattlesnake (S1 Table) using a Nikon D90 digital single lens reflex camera and a Nikon 105mm f/2.8G ED-IF AF-S VR Micro-Nikkor Lens. Of these, 2,824 (89.1%) were quantified, effectively spanning the range of each as an OTU (operational taxonomic unit).

**Fig 3:**
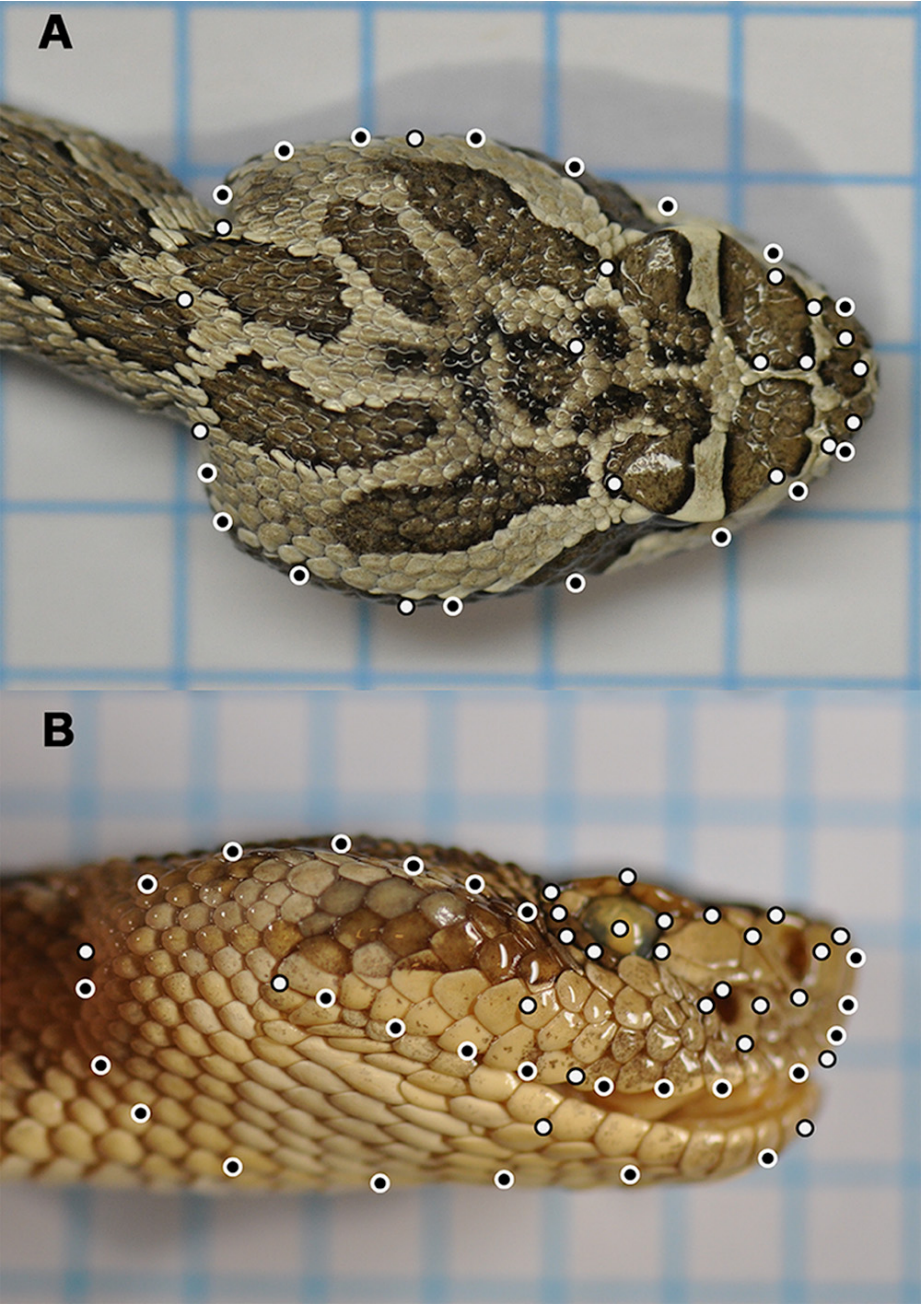
Lateral and dorsal landmarks used for subsequent shape analysis in the Western Rattlesnake complex. A total of 33 dorsal and 50 lateral landmarks were selected to assess shape in the Western Rattlesnake complex. Landmarks were composed of both Type I (white) and sliding semi (black) landmarks.

A total of 33 dorsal and 50 lateral homologous landmarks were identified and digitized on 2,824 individuals using the tpsDIG2 program [25] (Fig 3). Landmarks consisted of both “fixed” and “sliding” semi-landmarks [26–27], defined by their Cartesian coordinates. Generalized Procrustes Analyses (GPA; [28–29]) were applied to equilibrate landmark configurations with regard to size, orientation, and position, such that variation among landmarks was attributed only to variation in shape. This is a critical yet often overlooked consideration in GM analyses, in that it promotes the use of collections and specimens that vary in age and method of preservation. Minimum bending energy was used to slide landmarks, and their configurations were then aligned and centered by GPA (and termed “Procrustes residuals”) so as to depict individuals in “shape space.” Values from this non-Euclidean space were then projected into its tangent space so as to yield shape variables applicable for statistical analyses [30–31]

The lateral landmark configurations included anatomical landmarks associated with an articulated structure, the mandible. This angle of articulation must be accommodated so as to eliminate its respective non-shape landmark variation in the overall configuration. To do so, we applied the “fixed angle” method [32] that aligns the angle of articulation (arbitrarily set at 40°) prior to GPA.

Additionally, after GPA, we sought to determine if lateral and dorsal landmark configurations could be considered integrated subsets of a combined head shape. To test for morphological integration, we performed a two-block partial least squares (PLS) analysis between dorsal and lateral landmark configurations [33]. PLS indicated significant correlation (*r*_PLS_ = 0.5485, *P* = 0.0001), suggesting dorsal and lateral shapes were integrated and an independent analysis of each would be inappropriate. Therefore, dorsal and lateral landmarks were combined into a single data set by applying the modified “separate subsets” method [32]. Each was scaled by the ratio of the subset centroid size to the sum of both for each subject; i.e.,

$$y_{T}^{t}=\left[\begin{array}{cc} \frac{CS_{D}}{CS_{D}+CS_{L}}y_{D}^{t} & \frac{CS_{L}}{CS_{D}+CS_{L}}y_{L}^{t} \end{array}\right],$$

where subscripts _*D*_, _*L*_, and _*T*_, correspond to dorsal, lateral, or total components, respectively, of either the centroid size (*CS*) or the vectorized matrix of Procrustes residuals (**y**) (i.e., each **y**^**t**^ is a row vector of specimen Procrustes residuals that can be compiled into a data matrix.) Thus, combined Procrustes residuals represent a configuration with unit centroid size.

Procrustes residuals were projected onto the first two PCs to visualize shape variation. A thin-plate spline (TPS; [34]) function was used to create “transformation” grids [35] that depicted the mean shape associated with different locations in the shape space (as projected into the PCs of the tangent space). We performed TPS on lateral and dorsal configurations separately.

### Data Analyses

Potential sources of head shape variation included species or subspecies designation (i.e., mutually exclusive models of genetic relatedness), sex, stage of development (juvenile or adult), and head size. We constructed all possible linear models that included each of the factors (species or subspecies, sex, stage) and head size as a covariate, with factor-covariate interactions. Head size was calculated as the log of the centroid size—the square root of the summed squared distances between anatomical landmarks and the centroid of the configuration—for the dorsal landmark configuration [36]. We performed a method of model selection (S1 Text) that identified head size and subspecies as sources of variation, but not their interaction, suggesting a common shape allometry among subspecies [37–42].

We performed a non-parametric (np) multivariate analysis of variance (MANOVA) on the common-allometry model using a randomized residual permutation procedure (RRPP) so as to test model effects and gauge their sizes [43]. This procedure also permitted the calculation of pairwise distances between least squares means for subspecies in every random permutation, and the uniqueness of observed subspecies shapes to be tested. We performed np-MANOVA with these pairwise contrasts using 10,000 random permutations of the RRPP (the observed case counting as one random occurrence). Observed effects and contrasts were considered significant if their *P*-values—estimated as percentiles in sampling distributions generated from RRPP—were less than an acceptable type I error rate of α = 0.05.

For every specimen, we employed two approaches to calculate the Bayesian posterior classification probabilities for assignment to subspecies: the first used equal prior probability of assignment to any subspecies, whereas the second used prior probabilities based on phylogenetic relatedness, as derived from phylogenetic covariances between subspecies, estimated from branch lengths. Prior probabilities were calculated as

$$\text{Pr}\left(k\right)=\frac{c_{jk}}{\sum_{j=1}^{k}c_{jk}},$$

where *c* is the phylogenetic covariance between the *j*^th^ and *k*^th^ subspecies of the *k* = 9 subspecies, based on patristic distances between them assuming a Brownian motion model of evolution [44]. For any specimen, the *k* = 9 prior probabilities were calculated as such from the sum of all covariances between the actual subspecies designation, *j*, and each of the *k* hypothetical subspecies. Posterior probabilities were then calculated as

$$\text{Pr}\left(k|y_{j}^{t}\right)=\frac{\text{Pr}\left(y_{j}^{t}|k\right)\text{Pr}\left(k\right)}{\text{Pr}\left(y_{j}^{t}\right)};$$

where ${\text{y}}_{i}{}^{\text{t}}$ is a (transposed) vector of Procrustes residuals; *k* is the target group for association; $\text{Pr}\left(y_{j}^{t}|k\right)$ is the multivariate normal density estimate, found as

$$\text{Pr}\left(y_{j}^{t}|k\right)=\frac{1}{\sqrt{2\pi \left|C_{W}\right|}}\text{exp}\left(-\frac{1}{2}d_{M}^{2}\right),$$

where *d*_*M*_ is the Mahalanobis distance between $y_{j}^{t}$ and the group *k* centroid, and **C**_*W*_ is the pooled within-group covariance matrix; $\text{Pr}\left(y_{j}^{t}\right)$ is the normalization constant, solved as $\text{Pr}\left(y_{j}^{t}\right)=\sum_{j=1}^{k}\text{Pr}\left(y_{j}^{t}|k\right)\text{Pr}\left(k\right)$ (i.e., the summation of all normal density estimates for all groups); and Pr(k) is the prior probability as previously described [45].

We calculated inter-quartile ranges (IQRs) of posterior probabilities for each subspecies, based upon specimen assignments. Morphological distinction for subspecies was identified from IQRs that did not overlap between true and hypothetical comparisons, such that specimens could be assigned to subspecies.

To evaluate dispersion of subspecific means, we first regressed Procrustes residuals against centroid size to yield allometry-free shape values. We used these data in a principal component analysis (PCA) to estimate ancestral characters states using squared-change parsimony [46–48]. This allowed us to map both phylogeny and subspecies shapes within a PC space so as to test the potential for adaptive radiation. The latter was accomplished by compiling shape dispersion relative to estimated ancestral states. We using a disparity through time (DTT) analysis [49] to test the dispersion of mean shapes for evidence of adaptive radiation. This involved plotting the relative morphological disparity (i.e., average disparity within clades divided by total disparity) versus “relative time” (i.e., a scale of 0–1 from root to most recent divergence, based on the distribution of tree nodes). We then compared within-subclade relative disparities versus that expected under a Brownian motion model of trait evolution, using 10,000 simulations to find the median disparity through time and its 95% confidence limits.

All analyses were performed in R, version 3.1.2 [50], with GPA and TPS calculated using GEOMORPH, version 2.1.3 [51–52]. Estimating ancestral character states, pruning of the phylogenetic tree, and assessing the chronogram were performed using APE, version 3.0–14 [53]. DTT analysis was performed using GEIGER, version 2.0.3 [54]. PC plots with ellipsoids for within-subspecies shape variation were generated using RGL (ver. 0.92.1098) [55].

## Results

**Fig 4:**
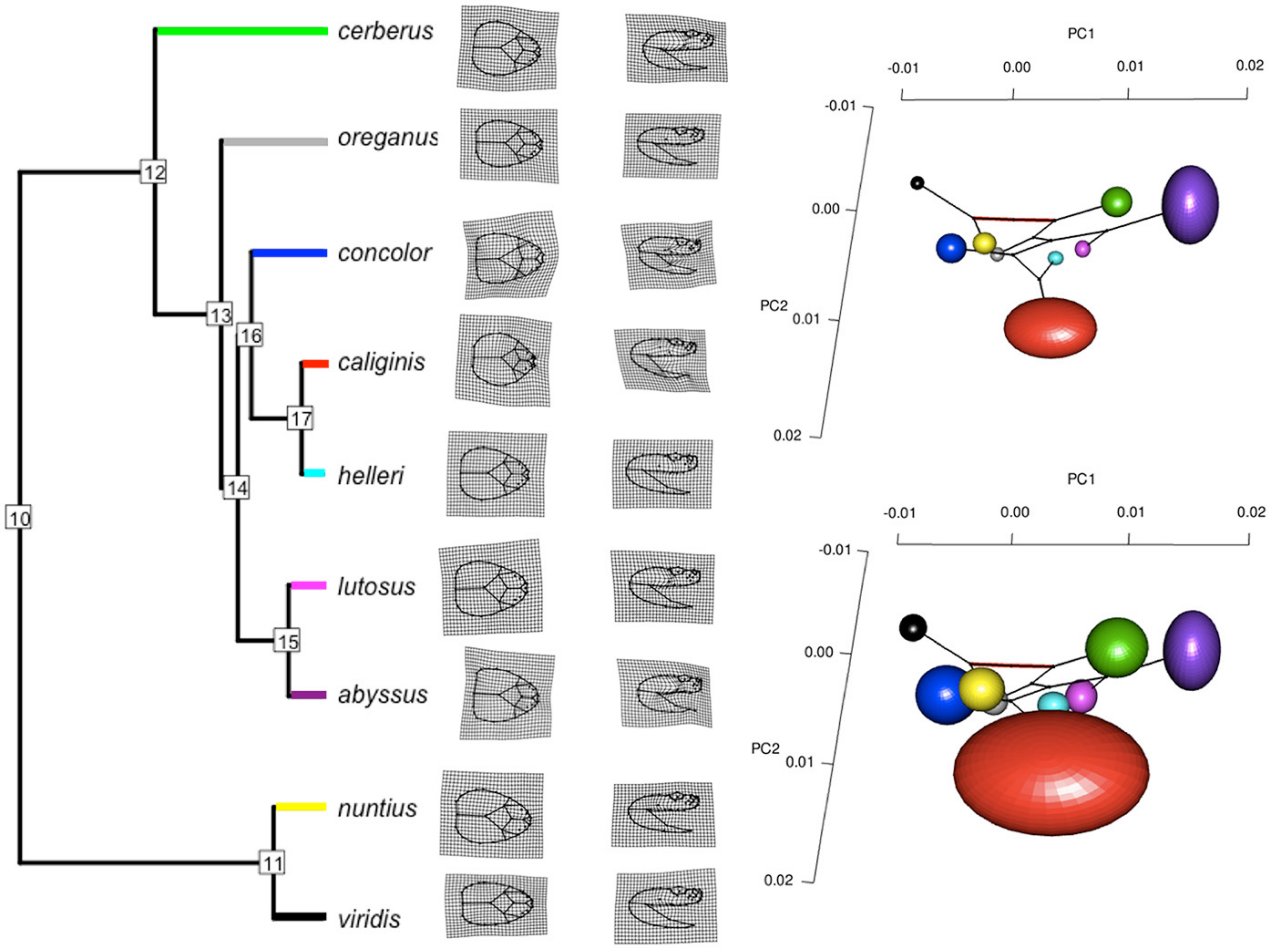
Phylogeny, transformation grids, and head shape variation within and among subspecies of the Western Rattlesnake (*Crotalus viridis*) complex. Left: A well-resolved Bayesian phylogenetic hypothesis was derived from six concatenated mtDNA sequences (to include an outgroup, *Crotalus scutulatus*, subsequently pruned from the tree). Nodes are numbered according to ancestral character states estimated (Table 1). Posterior node probabilities are 1.00, except for node 14, which is 0.99. Transformation grids that denote the deviation from mean form were derived for all nine subspecies using both dorsal and lateral landmark configurations. Transformation grids illustrate the shift from a more ovoid head shape in the eastern (*viridis* + *nuntius*) lineage to a more stereotypic, spearheaded morphology in the western lineage (*cerberus* + *oreganus* + *concolor* + *helleri* + *caliginis* + *lutosus* + *abyssus*). In addition, sister subspecies reflect a shift from an elongate snout and compressed head in the larger, more widespread form (i.e. *viridis*, *helleri*, and *lutosus*), to a shorter, more compact, and less compressed head shape in the diminutive forms (i.e. *nuntius*, *caliginis*, and *abyssus*, respectively). Right: The subspecies shapes correspond to mean positions in the among-subspecies PC plots. Ellipsoids represent scaling of one standard error of the mean (top) and 95% confidence limits (bottom) for each PC. The three PCs account for 58.0% of the variation among subspecies. Colors of ellipsoids match subspecies, as depicted in terminal branches of the phylogeny. The ancestral states and phylogeny edges are projected into the PC plots to facilitate interpretations. The bolder red edge corresponds to node 10, which separates clades.

Bayesian Analysis of six concatenated mtDNA sequences yielded a well-supported phylogenetic hypothesis of the Western Rattlesnake complex (Fig 4) that successfully accommodated differences among each, but also yielded a similar but more robust topography. The divergence of eastern and western lineages was unequivocal (1.00 posterior probability). Relationships between *viridis* and *nuntius* in the eastern lineage were also well supported (1.00 posterior probability). Within the western lineage, *C*. *v*. *cerberus* was recovered as basal (1.00 posterior probability), and with *oreganus* as sister (1.00 posterior probability). In addition, two well-supported clades were recovered sister to *oreganus*. These were: *concolor* and *lutosus* + *abyssus*, and *helleri* + *caliginis*. All relationships received posterior probabilities of 1.00, with the exception of the *concolor* and *lutosus* + *abyssus* clade (0.99).

**Fig 5:**
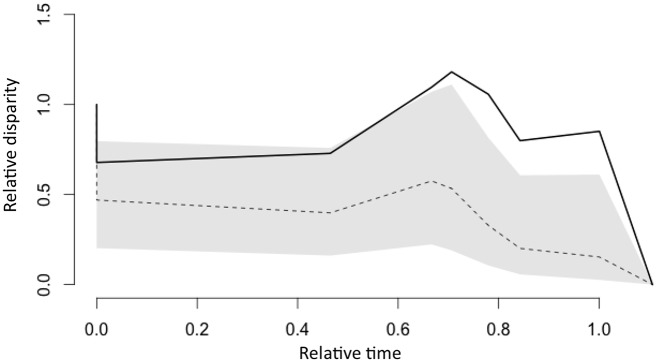
Results of a disparity through time (DTT) analysis plotted for the Western Rattlesnake (*Crotalus viridis*) complex. The solid line indicates the DTT of mean within-subclade head shapes. The dashed line indicated the median with the gray area representing 95% confidence limits, as derived from 10,000 simulations of Brownian evolution. The morphological disparity index (MDI), computed as average squared Euclidean distances between the DTT and median values, was 0.4271.

Both size and subspecies designation were significant sources of head shape variation based on np-MANOVA (Table 1). Subspecific variation was reflected by a diffuse but significant pattern of phenotypic spread in morphospace (Fig 4). Every pairwise head shape differed significantly among subspecies, except that between *C*. *v*. *abyssus* and *C*. *v*. *cerberus*. Every pairwise head shape difference (Procrustes distance) was also greater than any distance between ancestral character states (minimum *d* = 0.0020; maximum *d* = 0.0115), with the largest distance among ancestral states separating eastern and western clades (Table 2; Fig 4). Interestingly, the largest pairwise morphological distance was between sister taxa, *C*. *v*. *caliginis* and *C*. *v*. *concolor*. Furthermore, this pattern replicated itself, in that morphological divergences were similarly significant among all three sister-taxon comparisons. The greater within-subclade morphological disparity was confirmed by the DTT analysis, with much greater morphological divergence from a Brownian model of trait evolution than expected by chance along, particularly for nodes more shallow in configuration (Table 3, Fig 5).

**Table 1:** Results of a non-parametric multivariate analysis of variance (np-MANOVA) for head shape conducted on the Western Rattlesnake (*Crotalus viridis*) complex. Type I (sequential) sums of squares (= SS) were used to calculate sums of squares and cross-products matrices (SSCP) (see Supplemental Information for details). Head size = log(CS) whereas subspecific identification = Subspecies. *P*-values (= *P*) were computed from 10,000 random permutations of the randomization procedure. Z-values (= *Z*) are standard deviates of observed *SS* from sampling distributions. *MS* = Mean-Squares; *R*^2^ = coefficient of determination; *F* = F-statistic.

Source	*df*	*SS*	*MS*	*R*^2^	*F*	*Z*	*P*
log(CS)	1	0.4294	0.4294	0.0557	173.4930	83.0930	0.0001
Subspecies	8	0.3386	0.0423	0.0439	17.1020	16.0700	0.0001
Residuals	2803	6.9367	0.0025				
Total	2812	7.7046					

**Table 2:** Pairwise head shape comparisons among subspecies within the Western Rattlesnake (*Crotalus viridis*) complex. Pairwise subspecific shape differences (i.e., Procrustes distances) are arrayed below the diagonal, whereas *P*-values that stem from 10,000 random permutations of data are above the diagonal. The single non-significant Procrustes distance (i.e., *cerberus* versus *abyssus*) is depicted in bold, as is its corresponding *P*-value. Values in bold italics represent Procrustes distance comparisons among the three sister-taxa (i.e., lutosus/ abyssus; helleri/ calignis; viridis/ nuntius), with corresponding P-values in bold blue as well.

	abyssus	caliginis	cerberus	concolor	helleri	lutosus	nuntius	oreganus	viridis
abyssus		0.0001	**0.0642**	0.0001	0.0001	***0*.*0002***	0.0001	0.0001	0.0001
caliginis	0.0239		0.0001	0.0001	***0*.*0002***	0.0001	0.0002	0.0001	0.0001
cerberus	**0.0124**	0.0210		0.0001	0.0001	0.0001	0.0001	0.0001	0.0001
concolor	0.0276	0.0271	0.0249		0.0001	0.0001	0.0001	0.0001	0.0001
helleri	0.0177	***0*.*0181***	0.0134	0.0234		0.0001	0.0001	0.0001	0.0001
lutosus	***0*.*0170***	0.0220	0.0117	0.0211	0.0144		0.0001	0.0001	0.0001
nuntius	0.0226	0.0192	0.0170	0.0222	0.0117	0.0176		0.0001	***0*.*0001***
oreganus	0.0230	0.0213	0.0162	0.0235	0.0106	0.0134	0.0125		0.0001
viridis	0.0278	0.0263	0.0217	0.0241	0.0173	0.0219	***0*.*0119***	0.0150	

**Table 3:** Pairwise head shape comparisons among ancestral character states within the Western Rattlesnake (*Crotalus viridis*) complex. Distances compiled among ancestral character states are provided for comparisons. Numbers refer to ancestral nodes in the phylogeny (Fig 4)

	10	11	12	13	14	15	16	17
10								
11	0.0058							
12	0.0057	0.0115						
13	0.0048	0.0104	0.0024					
14	0.0036	0.0086	0.0043	0.0030				
15	0.0059	0.0086	0.0081	0.0072	0.0045			
16	0.0035	0.0087	0.0040	0.0025	0.0020	0.0062		
17	0.0037	0.0070	0.0069	0.0051	0.0039	0.0063	0.0035	

The first PC of among-subspecies shape variation (representing 32.6% of the variation) was associated with a general trend in head elongation. *Crotalus v*. *concolor* and *C*. *v*. *viridis* reflected elongated snouts and compressed head shapes compared to the shorter snout and less-compressed shape of *C*. *v*. *abyssus*. The second PC (15.3% of the variation) was associated with snout depression, as indicated by the wider but more flattened snout of *C*. *v*. *caliginis* in relation to other subspecies. The third PC (10.0% of variation) was associated with lower jaw morphology, with higher scores corresponding to shorter and more tapered lower jaws, as exhibited by *C*. *v*. *caliginis* and *C*. *v*. *abyssus*, versus the longer and more robust lower jaws of *C*.*v concolor*, *helleri*, and *lutosus*.

The dispersion of means supported the two-clade phylogeny as revealed by genetic data, with inter-clade distances greater than those recorded intra-clade. Superimposing the phylogeny onto the morphospace also elucidated head shape divergence in terminal taxa and suggested the potential for character displacement among sister taxa, consistent with the large significant differences between sister-pairs (Table 2). At the individual level, all subspecies save *C*. *v*. *abyssus* were supported by high posterior classification probabilities based on equal and phylogentically informed prior probabilities. When using equal prior probabilities, all subspecies were again distinct save for *C*. *v*. *abyssus*, which exhibited a higher posterior probability of being assigned to either *C*. *v*. *cerberus* or *C*. *v*. *lutosus* (S1 Fig).

**Fig 6:**
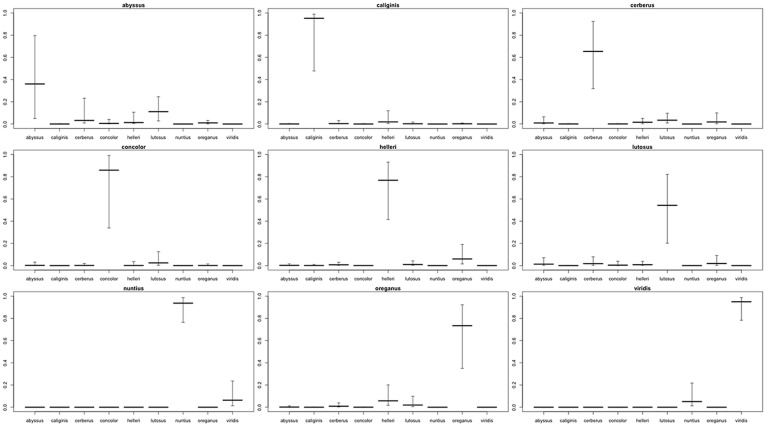
Plots depicting Bayesian posterior probabilities of assigning specimens to subspecies with phylogenetically informed prior probabilities in the Western Rattlesnake (*Crotalus viridis*) complex. Interquartile ranges are shown as error bars, with median values denoted by bolder notches. Each plot indicates the actual subspecific designation in the title.

The distinctiveness of subspecies in posterior probabilities was enhanced with the incorporation of phylogenetic relatedness (Fig 6). Again, *C*. *v*. *abyssus* could be misclassified as either *C*. *v*. *cerberus* or *C*. *v*. *lutosus*, but the third quartile posterior probabilities of assignment were quite low (*Q*_3_ = 0.2315, 0.2456, respectively), even compared to the median posterior probability of correct classification (*Q*_3_ = 0.3608). As expected, the IQR for *C*. *v*. *abyssus* assigned to *C*. *v*. *lutosus* was slightly higher than *C*. *v*. *abyssus* assigned to *C*. *v*. *cerberus*. However, as noted, the IQRs corresponded to low probabilities and neither alternative subspecies could be reciprocally classified as *C*. *v*. *abyssus*.

## Discussion

### Evolutionary history of the Western Rattlesnake

Despite two centuries of accumulating evidence, a comprehensive and formal reevaluation of Western Rattlesnake taxonomy has been slow to emerge. This reticence has been driven largely by three distinct, interrelated factors: (1) a long-standing disagreement over species concepts; (2) the convoluted history of this particular complex; and (3) an “essential tension” separating taxonomy and systematics. The first two are most relevant to our discussion and are parsed below.

By consensus, ‘species concepts’ represent the assumptions necessary for the recognition of morphological and genetic gaps among species [56, p. 447–472]. While these concepts do not delineate the gaps themselves, they provide a framework to interpret observed patterns [57–58], with the underlying premise that most, but not all, represent our attempts to explain or decipher how species came to be. This situation has been a source of confusion in that it conflates pattern with process. Our tact herein is to delineate gaps that correctly identify species rather than ponder mechanisms underlying the diversification.

The convoluted taxonomic history of the Western Rattlesnake complex is epitomized by the fact that no fewer than seven junior synonyms, 11 synonymizations, and 11 subspecific epithets have been applied to encompass its diversity [16, 59]. Fossil evidence for the complex is first recorded from the Late Pliocene [60] and extends through Late Pleistocene [61]. Molecular genetic data suggested a mid-Cenozoic origin, concomitant with the Neogene uplift of the North American Cordillera [62] presumably promoting the separation of eastern and western lineages, followed by constrictions and isolations within refugia during Pleistocene [16, 63] that drove additional differentiation within each. Shallow genetic divergence and relatively low diversity were manifested in subsequent radiations [16], resulting in species boundaries being obscured by incomplete lineage sorting [64] and ancestral polymorphism [65]. Both are artifacts prevalent in geologically recent and rapid diversifications, and they promote incongruent phylogenies and misinterpreted diversities when molecular data are used to derive taxonomic hypotheses.

### Evolutionary divergence in the Western Rattlesnake

Our GM analyses employed dorsal and lateral configurations of the head, as well as semi-landmarks that capture shape via the use of outlines. Our results indicated that both size and subspecies designation are significant sources of head shape variation in the Western Rattlesnake complex. This, in turn, suggests that head shape has both ontogenetic and phylogenetic components, with the former being intuitively expected [66–67], whereas the latter was somewhat surprising, particularly given that head shape in snakes has been recorded as diagnostic only among genders [68–69], or within broadly based trophic guilds [70], some of which contained several species within a single genus [71].

To further dissect this relationship, we then evaluated all subspecies in pairwise comparisons (N = 36), and found significantly different head shapes for each, save the *C*. *v*. *abyssus/ C*. *v*. *cerberus* evaluation (*P* = 0.064; bold red, Table 2). We note that head shape variation has previously been recorded as diagnostic among species of a genus [72], yet without statistical significance. Such a diagnosis among individual subspecies, as recorded herein, is thus of considerable interest, and may stem from the combination of dorsal/ lateral/ and sliding landmarks, as employed in our GM analyses [72].

We also extended our shape analyses by applying a Bayesian Discriminant Function Analysis with uninform priors, and found that all subspecies reflected high posterior classification probabilities, with the exception of *C*. *v*. *abyssus* and its higher posterior probability of being assigned to either *C*. *v*. *cerberus* or *C*. *v*. *lutosus*. This is not surprising as they are sister taxa in the phylogeny (Fig 4). However, their head shapes are dissimilar with low posterior probabilities for the assignment of *C*. *v*. *lutosus* to *C*. *v*. *abyssus* (Q1 = 0.0015, Q3 = 0.0610). We interpret this as a reflection of the large 95% confidence limits that bracket *C*. *v*. *abyssus*, an aspect that adds an element of imprecision to individual assignments. In contrast, the head shapes of *C*. *v*. *abyssus* and *C*. *v*. *cerberus* are similar, and one might suggest homoplasy as a potential factor. However, the posterior probabilities for assigning *C*. *v*. *cerberus* to *C*. *v*. *abyssus* are again quite small (Q1 = 0.0025, Q3 = 0.0943), and this lack of reciprocity thus fails to support the assertion of homoplasy.

We also employed phylogenetically informed prior probabilities based upon an mtDNA phylogeny so as to improve our Bayesian posterior classification (Fig 6), and again noted a tendency for *C*. *v*. *abyssus* to assign with *C*. *v*. *cerberus*/*C*. *v*. *lutosus*. As before, posterior probabilities for the assignment of *C*. *v*. *cerberus* or *C*. *v*. *lutosus* to *C*. *v*. *abyssus* were again reduced, and neither could be correctly assigned to *abyssus*. We interpret this as suggesting (but not confirming) a distinct head shape in *C*. *v*. *abyssus*. While we recognize this may reflect instead its small sample size in our analyses, we also reiterate that its head shape is not homoplasious with either *C*. *v*. *cerberus* or *C*. *v*. *lutosus*.

### Historic head shape and its contemporary divergence

Our depiction of evolutionary history in the *C*. *viridis* complex has focused rather intently on the contemporary morphospace of a mosaic structure, the head. Given this, we broadened and extended our analyses by (essentially) reconstructing an ancestral phenotype [as in 73] that potentially would have existed at each bifurcation of the tree (represented as numbered nodes in Fig 4). We accomplished this by employing squared change parsimony (a process that parallels maximum-likelihood but with a model of character evolution that invokes Brownian motion [74]) as a means to visualize an ancestral head shape). The process does not reconstruct outgroups or terminal taxa; those data were previously used in the estimation of ancestral character states. Rather, it acquires these comparisons by minimizing the sum of squared shape-changes along the branches of the phylogeny. This process assesses phenotypic divergences among terminal taxa as well as their estimated ancestral states. The assumptions and/or limitations in this process are several, but they are also tractable, such as: A constant rate of change (and a Brownian model of evolution); a correlation between neutral genetic and shape variation; and a deterioration in the reconstruction of character states as evolutionary time increases.

The results of these analyses indicated that pairwise shape distances among extant OTUs were significantly greater than those derived for ancestral character states, to include the ancestral node separating eastern and western lineages (Fig 4). In short, the closer the relationship between subspecies-pairs, the greater their differences in head shape. There are two mechanisms that can promote such a divergence among conspecifics: (a) competition, such that trophic character displacement is induced; or (b) a variance in foraging strategy that, in turn, promotes a shift in trophic ecology and, potentially, a dimorphism in size. Evidence from comparative analyses supports the latter [75], although its context relates to sexual dimorphism within and among snakes (and ramifications thereof), rather than to morphological divergence among groups in the broader sense (as herein). We explore this topic further in the section below.

### Molecular/ morphological disparity and hypotheses

Evolutionary theory predicts a tight coupling between molecular divergence and morphological disparity, such that clades with large numbers of species (and thus elevated molecular variability) exhibit an equally broad morphological disparity [76]. Yet empirical studies do not bear this out [32,73], and in this sense, the Western Rattlesnake complex is another example of morphological disparity surpassing molecular genetic variation.

Specifically, we observed greater shape disparity between sister taxa (e.g. *helleri* + *caliginis*; *lutosus* + *abyssus*; *viridis* + *nuntius*) than we did for genetically less proximate pairings. Distances were significantly greater than expected under a Brownian model of evolution (Fig 5), and in turn, suggest an evolutionary force other than drift that underpins shape divergence. Interestingly, sister-pairs in the *C*. *viridis* complex are generally composed of a larger form and one more diminutive or stunted [77]. Thus, *caliginis* is recognized as a diminutive insular form of *helleri*, while *abyssus* is a stunted *lutosus*, and *nuntius* a dwarfed *viridis*. In each case, the geographic range of the stunted form is greatly reduced when compared to the larger sister taxon. Their trophic differences are also recognized [78] and provide empirical (but anecdotal) evidence in support of the argument that ecological competition rather than dispersal or environmental constraints is the primary factor that limits species distributions across large spatial and temporal scales [79].

Indeed, separation according to overall body size is readily achieved [80] in that size is minimally impacted by evolutionary stasis [81], and hence more easily malleable than overall shape, or shape-specific morphological structures. Body size drives the separation of sister-pairs over evolutionary time (as above), and diversifies species as they adaptively radiate [82]. However, rapid evolution is seldom observable and thus rarely detectable in fossil lineages [83], hence its recognition is always “after the fact.” We suggest that morphological displacement along a particular trajectory represents the residual of past competitive interactions and these are more easily detectable using GM approaches (as herein, [83–84]).

Our results demonstrate that morphological divergence between sister-pairs is greater in all cases than that derived overall. The head shapes of each OTU is distinct (save for *abyssus*), with the caveat that reciprocal assignment of one sister taxon to the other is not supported. We thus hypothesize that the significant disparity between sister-pairs in the *C*. *viridis* complex is both driven by body size and is resource-based. As such, divergence among OTUs represents the product of historical interactions, as recoverable in their morphologies.

### Integrative taxonomy and the Western Rattlesnake

The *C*. *viridis* complex clearly requires taxonomic revision, yet this process has been problematic, with numerous attempts yielding either negative or incomplete results ([85] p. 569–570, [86]). Herein, we address this issue with a combined molecular and GM approach, as couched within a genotypic cluster criterion. And while we recognize the prescience of Klauber [87] in diagnosing nine OTUs to subspecies, we deem his approach as conservative in that it is based more on inferred intergradation than on quantitative differences. Klauber was trained as an electrical engineer and (we suggest) he refrained from diagnosing OTUs to species because he did not wish to counter the prevailing interpretation of a species-complex as being a ‘Rassenkreis’ (i.e., a ring of species, concomitant with expectations of intergradation among forms) [2,16]. Yet, at the same time, Klauber recognized ([18] p.164) that “…some of the newer methods of blood and venom studies may eventually indicate that the forms which we now consider *viridis* subspecies may really belong to two or more different species.” He seemingly realized the considerable weight of evidence differentiating these organisms, and understood that future studies might result in their re-classification as species.

We also recognize that a juxtaposition of legacy-based characters with more contemporary mtDNA, distributional, and GM data argues for a taxonomic reevaluation of the complex. Given this, we employed divergent data and methodologies so as to hypothesize species boundaries within a comprehensive and evolving framework termed ‘integrative taxonomy’ [88]. A review of integrative taxonomy identified 494 published studies over a seven-year period, with 233 of these (47%) using two different character types for discrimination [89]. Of these, 210 (90%) utilized DNA and morphology (as herein). Additionally, some 143 studies utilized vertebrates as study organisms, with 30% evaluating lepidosaurians (i.e., snakes, lizards, amphisbaenians, *Tuatara*), whereas another 30% assessed amphibians.

While an integrative approach has been influential in deriving species-boundaries in reptiles, its application has been multifaceted [88]. Some researchers suggest that concordance among unlinked molecular and morphological data is a requisite for species-delineation, in that it promotes taxonomic stability. Yet, one potential limitation is the risk of underestimating species numbers, with an assumption that characters evolve asynchronously within the speciation continuum. Others argue instead that integrative taxonomy should employ in its evaluation any available biological attribute, as long as those data provide evidence for the existence of a species. This, in turn, allows characters deemed most appropriate for a particular group to be utilized. Here, limitations stem from the application of a single line of discriminatory evidence (such as mtDNA), with an overestimation of species-numbers as a potential result. We elected to follow the first approach, with lineage divergence gauged across multiple characteristics and with the functional relevance of these data underscored in the speciation process [88].

Regardless, the application of integrative taxonomy necessitates a three-step protocol, as follows: (a) the accumulation of various data across numerous specimens; (b) the demarcation of groups using concepts that properly delimit species; and (c) the designation of nomenclature so as to recognize the new entities. However, one salient issue that emerged [90] was that a formal nomenclatural designation of a species failed to follow its character diagnosis. This discrepancy is rather disturbing for conservation, and thus we address this issue for the *C*. *viridis* complex in the following sections.

### Taxonomic conclusions and recommendations

The integrative taxonomy that emerged from our comparison of mtDNA and geometric morphometric data was placed within the context of previous evaluations [16]. In so doing, we recognize six (of nine) *C*. *viridis* OTUs as phylogenetic species. We also supply a formal taxonomic nomenclature (Table 4) that defines their separate evolutionary trajectories, with additional amplification (below) regarding those OTUs subsumed into one of the six designated species:

**Table 4:** A revised taxonomy for six species of the *Crotalus viridis* complex based upon molecular and morphological data and an integrated taxonomy approach. Standard English names follow [1,7].

Taxon (binomial name)	Standard English name
*Crotalus viridis* (Rafinesque, 1818)	Prairie Rattlesnake
*Crotalus oreganus* Holbrook, 1840	Northern Pacific Rattlesnake
*Crotalus cerberus* (Coues, 1875)	Arizona Black Rattlesnake
*Crotalus helleri* Meek, 1905	Southern Pacific Rattlesnake
*Crotalus concolor* Woodbury, 1929	Midget Faded Rattlesnake
*Crotalus lutosus* Klauber, 1930	Great Basin Rattlesnake

Our combined data and that of others validate two highly divergent lineages of *C*. *viridis* (eastern and western, respectively). The eastern group is recognized as *C*. *viridis*, but with *C*. *v*. *nuntius* subsumed within *C*. *viridis* (as before). However, we note that multiple lineages within the western clade reflect high mtDNA sequence divergence [16] as well as significant shape divergence (our data). Furthermore, these OTUs occur in relatively discrete geographic regions and seemingly reflect little intergradation or hybridization. Given this, and following a contemporary and theoretically well-grounded approach to species delineation (as above), we recommend the elevation of *cerberus*, *concolor*, *helleri*, *lutosus*, and *oreganus* to full species status, as based upon their reciprocal distinctiveness across both mtDNA and GM data.

The insular distribution of *C*. *v*. *caliginis* has previously been suggested as a condition sufficient for specific status [91], and our GM data support this as well. However, a close association between *calignis* and *helleri*, at the mtDNA level [14–16] suggests instead *calignis* is not sufficiently divergent at the molecular level and should instead be designated as an insular population of *C*. *helleri*. The situation is similar with *C*. *v*. *abyssus*, but with discriminating data in the integrative taxonomy essentially reversed. In this sense, *abyssus* was previously suggested as worthy of species-recognition based upon mtDNA data [16], yet the GM data from the current analysis (S1 Fig, Fig 6) fail to provide a statistically significant (and thus corroborative) level of support. Given our combined and integrative approach fails to differentiate *abyssus*, we recommend it should instead be subsumed within *C*. *lutosus*.

### Implications for conservation and management

Species, subspecies, and ‘distinct population segments’ of vertebrates can be listed under the ESA [2,92], albeit with clear requisites. For example, the extent of scientific uncertainty associated with the listing must be explicitly recognized, such that the conservation decisions that ensue can be more readily advocated and accepted [93]. These issues have proven problematic for polytypic species complexes whose taxonomies have most often been qualitatively described from reduced numbers of individuals sampled ambiguously across broad geographic continua. These derivations conflicted with evolutionary and statistical methodologies and were thus inappropriate for conservation planning and the phylogenetic perspectives it required. Their recognition consequently deteriorated under more contemporary scrutiny, as did the potential listing proposals based on their older taxonomies [89]. This has been especially true for herpetological subspecies that are numerically rich but with numbers that have decreased ~50% over the last decade [89].

It is important to recognize the numerous benefits to habitat conservation that have accrued from such a reorientation, with management units (MUs) [89,95], evolutionarily significant units (ESUs) [92,94,95], or even cryptic but distinct species [2, herein] being defined by application of more contemporary methodologies. As a result, connectivity can be promoted, fragmentation minimized, and the conservation value of the habitat subsequently elevated if indeed such patterns represent composites across multiple species [63]. In this sense, the adaptive management of multiple taxa should promote, or at least sustain, the geographic integrity of a region [2].

Our considerations underscore the influence of taxonomy on conservation, but with several recognized imperatives: the necessity of a regional- rather than taxon-centric view of management, and the promotion of a taxonomy that is contemporary, well defined, and represents the best science available.

## Supporting Information

S1 Fig Plots depicting Bayesian posterior probabilities that stem from assigning specimens to subspecies under equal prior probabilities in the Western Rattlesnake (*Crotalus viridis*) complex. Interquartile ranges are shown as error bars, with median values denoted by bolder notches. Each plot indicates the actual subspecies in the title.
(TIFF)

S1 Table A listing of museums that provided specimens of Western Rattlesnake employed in this study, to include catalog number. Dorsal, lateral, and ventral head images were obtained from a total of 3,170 unique specimens of Western Rattlesnake from 10 institutions, including: School of Life Sciences, Herpetology Collection, Arizona State University (ASU); Monte L. Bean Life Science Museum, Brigham Young University (BYU); California Academy of Sciences, Department of Herpetology (CAS); Carnegie Museum, Department of Herpetology (CM); Illinois Natural History Survey (INHS); Museum of Comparative Zoology, Harvard University (MCZ); Museum of Northern Arizona (MNA); San Diego Society of Natural History (SDSNH); Amphibian and Reptile Collection, Department of Ecology and Evolutionary Biology, University of Arizona (UAZ); and Utah Museum of Natural History (UMNH). Species and subspecies designations were obtained via the respective museum’s catalogs. Individuals without full taxonomic information (i.e. genus, species, or subspecies) were excluded from subsequent analyses.
(DOCX)

S2 Table Mitochondrial DNA accession numbers for the *Crotalus viridis* complex. List of mitochondrial (mt) DNA sequences accessed in this study. A total of 117 Western Rattlesnake sequences, representing six mtDNA regions, were accessed and subsequently concatenated by subspecies, with these data yielding a Bayesian Inference phylogenetic hypothesis.
(DOCX)

S1 Text The concatenation and analysis of mitochondrial DNA for the *Crotalus viridis* complex, with best-supported models of sequence evolution provided. We created a phylogenetic hypothesis based on 6 concatenated mtDNA regions acquired from GenBank (S2 Table: ATPase 8&6 (ATP8&6: TN+G); cytochrome B (Cyt-b: HKY + G); Displacement Loop (D-loop: HKY+G+I); NADH dehydrogenase subunit 2 (ND2: TIM (Transition Model)+G); and NADH dehydrogenase subunit 4L (ND4L: TN93+G+I). We merged sequences using the concatenation function in *Geneious* R8 [37] and arrayed them according to their presence in the mitochondrial genome, moving clockwise from D-loop (at 12 o’clock). The final arrangement was: D-loop—Cyt-b—ND4L—ATP8—ATP6—ND2, with sequences aligned using default settings in *MUSCLE* v 3.8.31 [38] (e.g. Gap Opening Cost = 15, Gap Extending Cost = 7). The model of nucleotide substitution for each mtDNA region was determined using *ModelTest* v 3.7 [39], with the concatenated sequences imported and partitioned into *Mr*. *Bayes* 3.2.5 [40–42], and the respective model of evolution applied to each partition. The Western Rattlesnake phylogeny was then estimated using the following conditions: heated chains = 4 (temp = 0.2); run simultaneously for 5,100,00 iterations; random seed = 16,288; burn-in = 1,000,000 iterations; subsampling frequency = 5,000 iterations; results = 4,000 trees, [i.e., ((5,100,000–1,000,000) x 4 / 5,000)]; branch lengths = unconstrained. Convergence was achieved within the first 250,000 iterations, autocorrelation was negligible within 1,024 samples, and log-likelihood plot indicated sufficient mixing. The resulting phylogeny was used for analyses requiring a phylogenetic comparative approach.
(DOCX)
