# Correction: Deconstructing a Species-Complex: Geometric Morphometric and Molecular Analyses Define Species in the Western Rattlesnake (*Crotalus viridis*)

**DOI:** 10.1371/journal.pone.0149712

**Published:** 2016-02-12

**Authors:** Mark A. Davis, Marlis R. Douglas, Michael L. Collyer, Michael E. Douglas

S1 Text is an earlier version of that document. Please view the correct S1 Text below.

## Supporting Information

S1 Text The concatenation and analysis of mitochondrial DNA for the Crotalus viridis complex, with best-supported models of sequence evolution provided. We created a phylogenetic hypothesis based on 6 concatenated mtDNA regions acquired from GenBank (S2 Table: ATPase 8&6 (ATP8&6: TN+G); cytochrome B (Cyt-b: HKY + G); Displacement Loop (D-loop: HKY+G+I); NADH dehydrogenase subunit 2 (ND2: TIM (Transition Model)+G); and NADH dehydrogenase subunit 4L (ND4L: TN93+G+I). We merged sequences using the concatenation function in Geneious R8 [37] and arrayed them according to their presence in the mitochondrial genome, moving clockwise from D-loop (at 12 o’clock). The final arrangement was: D-loop—Cyt-b—ND4L—ATP8—ATP6—ND2, with sequences aligned using default settings in MUSCLE v 3.8.31 [38] (e.g. Gap Opening Cost = 15, Gap Extending Cost = 7). The model of nucleotide substitution for each mtDNA region was determined using ModelTest v 3.7 [39], with the concatenated sequences imported and partitioned into Mr. Bayes 3.2.5 [40–42], and the respective model of evolution applied to each partition. The Western Rattlesnake phylogeny was then estimated using the following conditions: heated chains = 4 (temp = 0.2); run simultaneously for 5,100,00 iterations; random seed = 16,288; burn-in = 1,000,000 iterations; subsampling frequency = 5,000 iterations; results = 4,000 trees, [i.e., ((5,100,000–1,000,000) x 4 / 5,000)]; branch lengths = unconstrained. Convergence was achieved within the first 250,000 iterations, autocorrelation was negligible within 1,024 samples, and log-likelihood plot indicated sufficient mixing. The resulting phylogeny was used for analyses requiring a phylogenetic comparative approach.
(DOCX)

## References

[1] Davis MA, Douglas MR, Collyer ML, Douglas ME (2016) Deconstructing a Species-Complex: Geometric Morphometric and Molecular Analyses Define Species in the Western Rattlesnake (*Crotalus viridis*). PLoS ONE 11(1): e0146166 doi:10.1371/journal.pone.0146166 26816132 10.1371/journal.pone.0146166 PMC4731396

