# Correction: Deconstructing a Species-Complex: Geometric Morphometric and Molecular Analyses Define Species in the Western Rattlesnake (*Crotalus viridis*)

**DOI:** 10.1371/journal.pone.0211753

**Published:** 2019-01-30

**Authors:** Mark A. Davis, Marlis R. Douglas, Michael L. Collyer, Michael E. Douglas

S3 Table and S3 File are omitted from the list of Supporting Information and can be viewed below. With this Correction, the authors provide additional data that support the article's results. The S3 File contains the aligned coordinates, i.e. the raw landmark data subjected to Generalized Procrustes Analysis, which are needed to replicate the study's findings. The S3 Table provides the metadata describing the contents of the data file.

## Supporting information

S3 Table Supplementary metadata. Metadata defining column headers for the data file that includes the aligned coordinates and centroid sizes for both dorsal and lateral landmark configurations for all specimens used in this study.
(CSV)

S3 File Supplementary dataset. The data file that includes the aligned coordinates and centroid sizes for both dorsal and lateral landmark configurations for all specimens used in this study.
(TXT)
