# Supplementary figures and images for: Deconstructing a Species-Complex: Geometric Morphometric and Molecular Analyses Define Species in the Western Rattlesnake (Crotalus viridis)
Source: PLoS One. 2016 Jan 27;11(1):e0146166. doi: 10.1371/journal.pone.0146166 (PMC4731396; doi:10.1371/journal.pone.0146166)

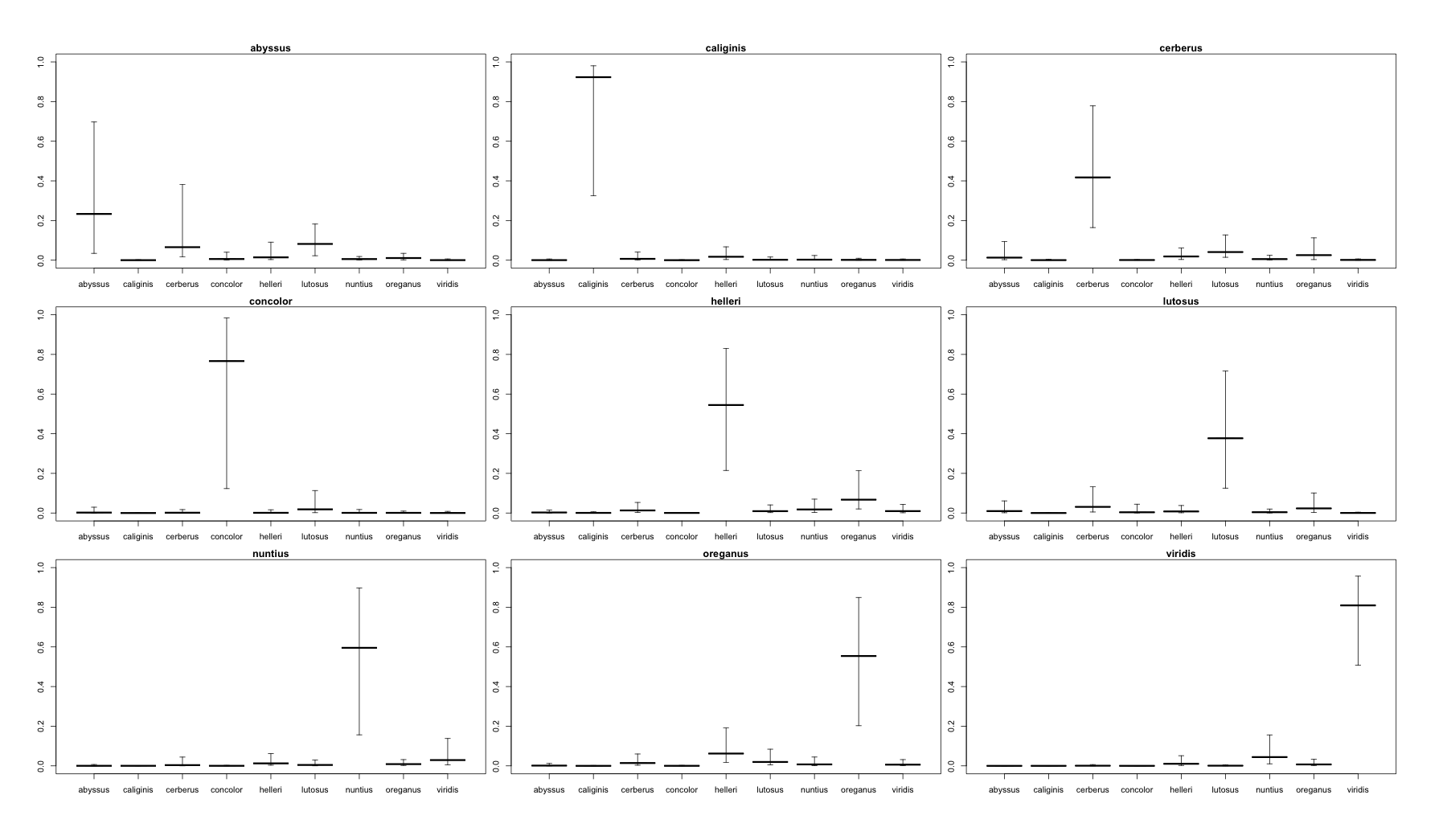

Supplement: S1 Fig — Interquartile ranges are shown as error bars, with median values denoted by bolder notches. Each plot indicates the actual subspecies in the title. (TIFF) [file pone.0146166.s001.tiff]
