# Supplementary material for: Deconstructing a Species-Complex: Geometric Morphometric and Molecular Analyses Define Species in the Western Rattlesnake (Crotalus viridis)
Source: PLoS One. 2016 Jan 27;11(1):e0146166. doi: 10.1371/journal.pone.0146166 (PMC4731396; doi:10.1371/journal.pone.0146166)
Supplement: S1 Table — Dorsal, lateral, and ventral head images were obtained from a total of 3,170 unique specimens of Western Rattlesnake from 10 institutions, including: School of Life Sciences, Herpetology Collection, Arizona State University (ASU); Monte L. Bean Life Science Museum, Brigham Young University (BYU); California Academy of Sciences, Department of Herpetology (CAS); Carnegie Museum, Department of Herpetology (CM); Illinois Natural History Survey (INHS); Museum of Comparative Zoology, Harvard University (MCZ); Museum of Northern Arizona (MNA); San Diego Society of Natural History (SDSNH); Amphibian and Reptile Collection, Department of Ecology and Evolutionary Biology, University of Arizona (UAZ); and Utah Museum of Natural History (UMNH). Species and subspecies designations were obtained via the respective museum’s catalogs. Individuals without full taxonomic information (i.e. genus, species, or subspecies) were excluded from subsequent analyses. (DOCX) [file pone.0146166.s002.docx]

**S1. List of Specimens, their museum of origin, and catalog number**

Dorsal, lateral, and ventral head images were obtained from a total of 3,170 unique specimens of Western Rattlesnake from 10 institutions, including School of Life Sciences, Herpetology Collection, Arizona State University (ASU); Monte L. Bean Life Science Museum, Brigham Young University (BYU), California Academy of Sciences, Department of Herpetology (CAS), Carnegie Museum, Department of Herpetology (CM), Illinois Natural History Survey (INHS); Museum of Comparative Zoology, Harvard University (MCZ); Museum of Northern Arizona (MNA), San Diego Society of Natural History (SDSNH); Amphibian and Reptile Collection, Department of Ecology and Evolutionary Biology, University of Arizona (UAZ), and Utah Museum of Natural History (UMNH). Species and subspecies designations were obtained via the respective museum’s catalogs. Individuals without full taxonomic information (i.e. genus, species, or subspecies) were excluded from subsequent analyses.

| **Institution** | **Catalog Number** | **Genus** | **species** | **subspecies** | **Country** | **State** | **County** |
| --- | --- | --- | --- | --- | --- | --- | --- |
| ASU | 4 | Crotalus | oreganus | cerberus | USA | Arizona | Gila |
| ASU | 121 | Crotalus | oreganus | helleri | USA | California |  |
| ASU | 490 | Crotalus | oreganus | oreganus | USA | Oregon | Benton |
| ASU | 539 | Crotalus | oreganus | oreganus | USA | Oregon | Benton |
| ASU | 539 | Crotalus | oreganus | oreganus | USA | Oregon | Benton |
| ASU | 540 | Crotalus | oreganus | oreganus | USA | Oregon | Benton |
| ASU | 785 | Crotalus | oreganus | cerberus | USA | Arizona |  |
| ASU | 787 | Crotalus | oreganus | cerberus | USA | Arizona |  |
| ASU | 986 | Crotalus | oreganus | cerberus | USA | Arizona |  |
| ASU | 1293 | Crotalus | oreganus | oreganus | USA | Oregon |  |
| ASU | 1294 | Crotalus | oreganus | oreganus | USA | Oregon |  |
| ASU | 1457 | Crotalus | oreganus | cerberus | USA | Arizona | Pinal |
| ASU | 1469 | Crotalus | oreganus | cerberus | USA | Arizona | Yavapai |
| ASU | 1865 | Crotalus | oreganus | cerberus | USA | Arizona | Yavapai |
| ASU | 1866 | Crotalus | oreganus | cerberus | USA | Arizona | Navajo |
| ASU | 1928 | Crotalus | oreganus | oreganus | USA | Oregon |  |
| ASU | 1942 | Crotalus | oreganus | lutosus | USA | Nevada | White Pine |
| ASU | 1943 | Crotalus | oreganus | lutosus | USA | Nevada | White Pine |
| ASU | 1944 | Crotalus | oreganus | lutosus | USA | Nevada | White Pine |
| ASU | 1945 | Crotalus | oreganus | lutosus | USA | Nevada | White Pine |
| ASU | 1946 | Crotalus | oreganus | lutosus | USA | Nevada | White Pine |
| ASU | 1947 | Crotalus | oreganus | lutosus | USA | Nevada | White Pine |
| ASU | 1948 | Crotalus | oreganus | lutosus | USA | Nevada | White Pine |
| ASU | 1949 | Crotalus | oreganus | lutosus | USA | Nevada | White Pine |
| ASU | 1950 | Crotalus | oreganus | lutosus | USA | Nevada | White Pine |
| ASU | 1951 | Crotalus | oreganus | lutosus | USA | Nevada | White Pine |
| ASU | 1952 | Crotalus | oreganus | lutosus | USA | Nevada | White Pine |
| ASU | 1953 | Crotalus | oreganus | lutosus | USA | Nevada | White Pine |
| ASU | 1954 | Crotalus | oreganus | lutosus | USA | Nevada | White Pine |
| ASU | 1955 | Crotalus | oreganus | lutosus | USA | Nevada | White Pine |
| ASU | 1981 | Crotalus | oreganus | cerberus | USA | Arizona | Gila |
| ASU | 1991 | Crotalus | oreganus | oreganus | USA | Oregon |  |
| ASU | 1991 | Crotalus | oreganus | oreganus | USA | Oregon |  |
| ASU | 2150 | Crotalus | oreganus | oreganus | USA | Oregon |  |
| ASU | 2181 | Crotalus | oreganus | cerberus | USA | Arizona | Yavapai |
| ASU | 2238 | Crotalus | oreganus | cerberus | USA | Arizona | Yavapai |
| ASU | 2238 | Crotalus | oreganus | cerberus | USA | Arizona | Yavapai |
| ASU | 2344 | Crotalus | oreganus | cerberus | USA | Arizona | Yavapai |
| ASU | 2360 | Crotalus | oreganus | cerberus | USA | Arizona | Yavapai |
| ASU | 2360 | Crotalus | oreganus | cerberus | USA | Arizona | Yavapai |
| ASU | 2393 | Crotalus | oreganus | cerberus | USA | Arizona | Yavapai |
| ASU | 2419 | Crotalus | viridis | nuntius | USA | Arizona | Coconino |
| ASU | 2632 | Crotalus | oreganus | cerberus | USA | Arizona | Yavapai |
| ASU | 2727 | Crotalus | viridis | viridis | USA | Montana | Cascade |
| ASU | 2728 | Crotalus | oreganus | helleri | USA | California | San Diego |
| ASU | 2728 | Crotalus | oreganus | helleri | USA | California | San Diego |
| ASU | 2775 | Crotalus | viridis | viridis | USA | Montana | Cascade |
| ASU | 2806 | Crotalus | viridis | viridis | USA | Montana | Cascade |
| ASU | 2870 | Crotalus | viridis | viridis | USA | Montana | Cascade |
| ASU | 3590 | Crotalus | oreganus | cerberus | USA | Arizona | Coconino |
| ASU | 3634 | Crotalus | oreganus | cerberus | USA | Arizona |  |
| ASU | 3635 | Crotalus | oreganus | cerberus | USA | Arizona |  |
| ASU | 3636 | Crotalus | oreganus | cerberus | USA | Arizona |  |
| ASU | 3653 | Crotalus | oreganus | cerberus | USA | Arizona | Apache |
| ASU | 3688 | Crotalus | oreganus | lutosus | USA | California | Modoc |
| ASU | 3690 | Crotalus | viridis | nuntius | USA | Arizona | Coconino |
| ASU | 3692 | Crotalus | oreganus | oreganus | USA | Washington | Okanogan |
| ASU | 3692 | Crotalus | oreganus | oreganus | USA | Washington | Okanogan |
| ASU | 3693 | Crotalus | oreganus | oreganus | USA | Washington | Okanogan |
| ASU | 3695 | Crotalus | oreganus | oreganus | USA | Washington | Okanogan |
| ASU | 3696 | Crotalus | oreganus | oreganus | USA | Washington | Okanogan |
| ASU | 3697 | Crotalus | oreganus | oreganus | USA | Washington | Okanogan |
| ASU | 3698 | Crotalus | oreganus | cerberus | USA | Arizona | Coconino |
| ASU | 3698 | Crotalus | oreganus | oreganus | USA | Washington | Okanogan |
| ASU | 4136 | Crotalus | viridis | nuntius | USA | Arizona | Apache |
| ASU | 4235 | Crotalus | oreganus | cerberus | USA | Arizona | Gila |
| ASU | 4238 | Crotalus | oreganus | cerberus | USA | Arizona | Gila |
| ASU | 4319 | Crotalus | oreganus | oreganus | USA | California | Yolo |
| ASU | 4384 | Crotalus | oreganus | cerberus | USA | Arizona | Gila |
| ASU | 4494 | Crotalus | oreganus | cerberus | USA | Arizona | Gila |
| ASU | 4494 | Crotalus | oreganus | cerberus | USA | Arizona | Gila |
| ASU | 4993 | Crotalus | oreganus | cerberus | USA | Arizona | Maricopa |
| ASU | 5048 | Crotalus | viridis | nuntius | USA | Arizona | Apache |
| ASU | 5050 | Crotalus | viridis | nuntius | USA | Arizona | Apache |
| ASU | 5051 | Crotalus | viridis | nuntius | USA | Arizona | Apache |
| ASU | 7038 | Crotalus | oreganus | cerberus | USA | Arizona | Graham |
| ASU | 7401 | Crotalus | oreganus | cerberus | USA | Arizona | Graham |
| ASU | 8874 | Crotalus | viridis | viridis | USA | Colorado | Huerfano |
| ASU | 9277 | Crotalus | oreganus | cerberus | USA | Arizona | Maricopa |
| ASU | 10500 | Crotalus | viridis | viridis | USA | Montana | Cascade |
| ASU | 10501 | Crotalus | viridis | viridis | USA | Montana | Cascade |
| ASU | 10502 | Crotalus | viridis | viridis | USA | Montana | Cascade |
| ASU | 10503 | Crotalus | oreganus | lutosus | USA | Nevada | White Pine |
| ASU | 10504 | Crotalus | oreganus | cerberus | USA | Arizona | Yavapai |
| ASU | 10505 | Crotalus | viridis | viridis | USA | Montana | Cascade |
| ASU | 10506 | Crotalus | viridis | viridis | USA | Montana | Cascade |
| ASU | 10507 | Crotalus | viridis | viridis | USA | Montana | Cascade |
| ASU | 10508 | Crotalus | viridis | viridis | USA | Montana | Cascade |
| ASU | 10508 | Crotalus | viridis | viridis | USA | Montana | Cascade |
| ASU | 10768 | Crotalus | oreganus | cerberus | USA | Arizona | Gila |
| ASU | 10770 | Crotalus | oreganus | cerberus | USA | Arizona | Gila |
| ASU | 10771 | Crotalus | oreganus | cerberus | USA | Arizona | Gila |
| ASU | 10772 | Crotalus | oreganus | cerberus | USA | Arizona | Gila |
| ASU | 13615 | Crotalus | scutulatus | | USA | New Mexico |  |
| ASU | 13972 | Crotalus | viridis | nuntius | USA | Arizona | Apache |
| ASU | 17337 | Crotalus | scutulatus | | USA | Arizona | Mojave |
| ASU | 17749 | Crotalus | oreganus | cerberus | USA | Arizona | Yavapai |
| ASU | 21136 | Crotalus | oreganus | lutosus | USA | Utah | Washington |
| ASU | 21141 | Crotalus | oreganus | lutosus | USA | Utah | Tron |
| ASU | 21311 | Crotalus | oreganus | lutosus | USA | Nevada | Lincoln |
| ASU | 21312 | Crotalus | oreganus | lutosus | USA | Utah | Tron |
| ASU | 21313 | Crotalus | viridis | viridis | USA | Utah | Garfield |
| ASU | 21326 | Crotalus | viridis | viridis | USA | New Mexico |  |
| ASU | 21413 | Crotalus | oreganus | oreganus | USA | Arizona | Maricopa |
| ASU | 22117 | Crotalus | viridis | viridis | USA | Texas | Patter |
| ASU | 22179 | Crotalus | oreganus | cerberus | USA | Arizona | Navajo |
| ASU | 22179 | Crotalus | oreganus | cerberus | USA | Arizona | Navajo |
| ASU | 22180 | Crotalus | oreganus | cerberus | USA | Arizona | Navajo |
| ASU | 22180 | Crotalus | oreganus | cerberus | USA | Arizona | Navajo |
| ASU | 22790 | Crotalus | viridis | nuntius | USA | Arizona | Coconino |
| ASU | 22791 | Crotalus | oreganus |  | USA | Arizona | Coconino |
| ASU | 22797 | Crotalus | oreganus | cerberus | USA | Arizona | Yavapai |
| ASU | 24314 | Crotalus | oreganus |  | USA | Arizona | Coconino |
| ASU | 27848 | Crotalus | oreganus | abyssus | USA | Arizona | Coconino |
| ASU | 28047 | Crotalus | oreganus | lutosus | USA | Arizona | Mohave |
| ASU | 28073 | Crotalus | oreganus | abyssus | USA | Arizona | Coconino |
| ASU | 30984 | Crotalus | oreganus | helleri | USA | California | Los Angeles |
| ASU | 31500 | Crotalus | viridis | viridis | USA | New Mexico |  |
| ASU | 33148 | Crotalus | oreganus | abyssus | USA | Arizona | Coconino |
| ASU | 33149 | Crotalus | viridis | nuntius | USA | Arizona | Coconino |
| ASU | 33223 | Crotalus | viridis | nuntius | USA | Arizona | Apache |
| ASU | 33291 | Crotalus | oreganus | cerberus | USA | Arizona | Yavapai |
| ASU | 33292 | Crotalus | oreganus | cerberus | USA | Arizona | Gila |
| ASU | 33358 | Crotalus | viridis | nuntius | USA | Arizona | Coconino |
| ASU | 34813 | Crotalus | viridis | nuntius | USA | Arizona | Apache |
| ASU | 34815 | Crotalus | viridis | nuntius | USA | Arizona | Navajo |
| ASU | 34893 | Crotalus | oreganus | lutosus | USA | Arizona | Coconino |
| ASU | 39830 | Crotalus | oreganus | abyssus | USA | Arizona | Coconino |
| ASU | 75814 | Crotalus | unknown | unknown |  |  |  |
| ASU | 427w | Crotalus | scutulatus | | USA | New Mexico |  |
| BYU | 120 | Crotalus | oreganus | concolor | USA | Utah | Kane |
| BYU | 340 | Crotalus | oreganus | lutosus | USA | Utah | Cache |
| BYU | 349 | Crotalus | oreganus | lutosus | USA | Arizona |  |
| BYU | 350 | Crotalus | oreganus | lutosus | USA | Utah | Garfield |
| BYU | 352 | Crotalus | oreganus | lutosus | USA | Idaho | Ada |
| BYU | 355 | Crotalus | oreganus | lutosus | USA | Utah | Beaver |
| BYU | 364 | Crotalus | oreganus | concolor | USA | Utah | Kane |
| BYU | 364 | Crotalus | oreganus | concolor | USA | Utah | Kane |
| BYU | 575 | Crotalus | oreganus | concolor | USA | Utah | Garfield |
| BYU | 686 | Crotalus | oreganus | concolor | USA | Utah | Kane |
| BYU | 1284 | Crotalus | oreganus | lutosus | USA | Utah | Cache |
| BYU | 1290 | Crotalus | oreganus | lutosus | USA | Arizona |  |
| BYU | 1298 | Crotalus | oreganus | lutosus | USA | Idaho | Ada |
| BYU | 1299 | Crotalus | oreganus | lutosus | USA | Idaho | Ada |
| BYU | 1636 | Crotalus | oreganus | concolor | USA | Utah | Garfield |
| BYU | 1947 | Crotalus | oreganus | lutosus | USA | Utah | Kane |
| BYU | 1949 | Crotalus | oreganus | lutosus | USA | Utah | Kane |
| BYU | 2759 | Crotalus | oreganus | lutosus | USA | Idaho | Camas |
| BYU | 2760 | Crotalus | oreganus | concolor | USA | Utah | Kane |
| BYU | 4793 | Crotalus | oreganus | lutosus | USA |  |  |
| BYU | 4908 | Crotalus | oreganus | lutosus | USA |  |  |
| BYU | 4909 | Crotalus | oreganus | lutosus | USA |  |  |
| BYU | 4960 | Crotalus | oreganus | concolor | USA |  |  |
| BYU | 4974 | Crotalus | oreganus | lutosus | USA | Utah | San Juan |
| BYU | 5432 | Crotalus | unknown | unknown |  |  |  |
| BYU | 8276 | Crotalus | oreganus | lutosus | USA | Utah | Cache |
| BYU | 8277 | Crotalus | oreganus | lutosus | USA | Utah | Cache |
| BYU | 8278 | Crotalus | oreganus | lutosus | USA | Utah | Cache |
| BYU | 9070 | Crotalus | oreganus | lutosus | USA | Utah | Juab |
| BYU | 10849 | Crotalus | oreganus | lutosus | USA |  |  |
| BYU | 11260 | Crotalus | oreganus | concolor | USA | Utah | Kane |
| BYU | 12525 | Crotalus | oreganus | concolor | USA | Utah | Garfield |
| BYU | 13015 | Crotalus | oreganus | concolor | USA | Utah | Duchense |
| BYU | 13017 | Crotalus | oreganus | lutosus | USA | Utah | Box Elder |
| BYU | 14645 | Crotalus | oreganus | lutosus | USA | Nevada | Nye |
| BYU | 14699 | Crotalus | oreganus | concolor | USA | Utah | Grand |
| BYU | 14923 | Crotalus | oreganus | concolor | USA | Utah | Kane |
| BYU | 14962 | Crotalus | oreganus | lutosus | USA | Utah | Millard |
| BYU | 16534 | Crotalus | oreganus | concolor | USA | Utah | Emery |
| BYU | 16535 | Crotalus | oreganus | concolor | USA | Utah | Emery |
| BYU | 16536 | Crotalus | oreganus | concolor | USA | Utah | Emery |
| BYU | 16537 | Crotalus | oreganus | concolor | USA | Utah | Emery |
| BYU | 16747 | Crotalus | oreganus | concolor | USA | Utah | San Juan |
| BYU | 16804 | Crotalus | oreganus | lutosus | USA | Utah | Sanpete |
| BYU | 17981 | Crotalus | oreganus | lutosus |  |  |  |
| BYU | 17987 | Crotalus | oreganus | lutosus |  |  |  |
| BYU | 17987 | Crotalus | unknown | unknown |  |  |  |
| BYU | 18576 | Crotalus | viridis | nuntius | USA | Utah | San Juan |
| BYU | 18966 | Crotalus | oreganus | lutosus | USA | Nevada | Nye |
| BYU | 20744 | Crotalus | oreganus | lutosus | USA | Utah | Kane |
| BYU | 20745 | Crotalus | oreganus | lutosus | USA | Utah | Kane |
| BYU | 20747 | Crotalus | oreganus | lutosus | USA | Utah | Kane |
| BYU | 20748 | Crotalus | oreganus | lutosus | USA | Utah | Kane |
| BYU | 20749 | Crotalus | oreganus | lutosus | USA | Utah | Kane |
| BYU | 20751 | Crotalus | oreganus | concolor | USA | Utah | Emery |
| BYU | 21266 | Crotalus | viridis | viridis | USA | Colorado | Montezuma |
| BYU | 21392 | Crotalus | oreganus | concolor | USA | Utah | Grand |
| BYU | 21489 | Crotalus | oreganus | concolor | USA | Utah | Grand |
| BYU | 21985 | Crotalus | viridis | viridis | USA | Colorado | Montezuma |
| BYU | 23802 | Crotalus | oreganus | concolor | USA | Utah | Grand |
| BYU | 30779 | Crotalus | oreganus | lutosus | USA | Idaho | Butte |
| BYU | 30780 | Crotalus | oreganus | lutosus | USA | Idaho | Butte |
| BYU | 30781 | Crotalus | oreganus | lutosus | USA | Idaho | Butte |
| BYU | 30782 | Crotalus | oreganus | lutosus | USA | Idaho | Butte |
| BYU | 30783 | Crotalus | oreganus | lutosus | USA | Idaho | Butte |
| BYU | 30784 | Crotalus | oreganus | lutosus | USA | Idaho | Butte |
| BYU | 30785 | Crotalus | oreganus | lutosus | USA | Idaho | Butte |
| BYU | 30786 | Crotalus | oreganus | lutosus | USA | Idaho | Butte |
| BYU | 30787 | Crotalus | oreganus | lutosus | USA | Idaho | Butte |
| BYU | 30788 | Crotalus | oreganus | lutosus | USA | Idaho | Butte |
| BYU | 30788 | Crotalus | oreganus | lutosus | USA | Idaho | Butte |
| BYU | 30789 | Crotalus | oreganus | lutosus | USA | Idaho | Butte |
| BYU | 31187 | Crotalus | oreganus | concolor | USA | Utah | Grand |
| BYU | 32227 | Crotalus | oreganus | lutosus | USA | Utah | Beaver |
| BYU | 34601 | Crotalus | oreganus | lutosus | USA | California | Kings |
| BYU | 34661 | Crotalus | oreganus | concolor | USA | Utah | Garfield |
| BYU | 34662 | Crotalus | oreganus | concolor | USA | Utah | Garfield |
| BYU | 34699 | Crotalus | viridis | nuntius | USA | Utah | San Juan |
| BYU | 34700 | Crotalus | oreganus | lutosus | USA | Arizona | Mohave |
| BYU | 34700 | Crotalus | oreganus | lutosus | USA | Arizona | Mohave |
| BYU | 34749 | Crotalus | viridis | nuntius | USA | Utah | San Jaun |
| BYU | 34750 | Crotalus | viridis | nuntius | USA | Utah | San Jaun |
| BYU | 34751 | Crotalus | viridis | nuntius | USA | Utah | San Jaun |
| BYU | 34752 | Crotalus | viridis | nuntius | USA | Utah | San Jaun |
| BYU | 34753 | Crotalus | viridis | nuntius | USA | Utah | San Jaun |
| BYU | 34754 | Crotalus | viridis | nuntius | USA | Utah | San Jaun |
| BYU | 34755 | Crotalus | viridis | nuntius | USA | Utah | San Jaun |
| BYU | 34756 | Crotalus | viridis | nuntius | USA | Utah | San Jaun |
| BYU | 36928 | Crotalus | oreganus | concolor | USA | Utah | Carbon |
| BYU | 36929 | Crotalus | oreganus | lutosus | USA | Utah | Beaver |
| BYU | 36930 | Crotalus | oreganus | lutosus | USA | Utah | Beaver |
| BYU | 36931 | Crotalus | oreganus | lutosus | USA | Utah | Beaver |
| BYU | 36932 | Crotalus | oreganus | lutosus | USA | Utah | Beaver |
| BYU | 36945 | Crotalus | oreganus | lutosus | USA | Utah | Utah |
| BYU | 37097 | Crotalus | oreganus | lutosus | USA | Utah | Salt Lake |
| BYU | 37100 | Crotalus | oreganus | concolor | USA | Wyoming |  |
| BYU | 37104 | Crotalus | oreganus | lutosus | USA | Utah | Kane |
| BYU | 37109 | Crotalus | oreganus | lutosus | USA | Utah | Utah |
| BYU | 37678 | Crotalus | oreganus | concolor | USA | Wyoming | Sweetwater |
| BYU | 37679 | Crotalus | oreganus | concolor | USA | Wyoming | Sweetwater |
| BYU | 38372 | Crotalus | oreganus | lutosus | USA | Utah | Kane |
| BYU | 38375 | Crotalus | oreganus | concolor | USA |  |  |
| BYU | 38379 | Crotalus | viridis | nuntius | USA | Utah | San Juan |
| BYU | 39687 | Crotalus | oreganus | concolor | USA | Wyoming | Sweetwater |
| BYU | 39690 | Crotalus | oreganus | concolor | USA | Colorado | Rio Blanco |
| BYU | 39691 | Crotalus | oreganus | concolor | USA | Colorado | Rio Blanco |
| BYU | 41111 | Crotalus | oreganus | abyssus | USA | Arizona | Grand Canyon |
| BYU | 41386 | Crotalus | oreganus | concolor | USA | Wyoming | Sweetwater |
| BYU | 41387 | Crotalus | oreganus | concolor | USA | Wyoming | Sweetwater |
| BYU | 41388 | Crotalus | oreganus | concolor | USA | Wyoming | Sweetwater |
| BYU | 41389 | Crotalus | oreganus | concolor | USA | Wyoming | Sweetwater |
| BYU | 41390 | Crotalus | oreganus | concolor | USA | Wyoming | Sweetwater |
| BYU | 41391 | Crotalus | oreganus | concolor | USA | Wyoming | Sweetwater |
| BYU | 41398 | Crotalus | viridis | nuntius | USA | Utah | Garfield |
| BYU | 41399 | Crotalus | viridis | nuntius | USA | Utah | Garfield |
| BYU | 41400 | Crotalus | oreganus | concolor | USA | Utah | San Juan |
| BYU | 41400 | Crotalus | oreganus | concolor | USA | Utah | San Juan |
| BYU | 41401 | Crotalus | oreganus | concolor | USA | Wyoming | Sweetwater |
| BYU | 41402 | Crotalus | oreganus | concolor | USA | Wyoming | Sweetwater |
| BYU | 41403 | Crotalus | oreganus | concolor | USA | Wyoming | Sweetwater |
| BYU | 41405 | Crotalus | oreganus | concolor | USA | Wyoming | Sweetwater |
| BYU | 41406 | Crotalus | oreganus | concolor | USA | Wyoming | Sweetwater |
| BYU | 41407 | Crotalus | oreganus | concolor | USA | Wyoming | Sweetwater |
| BYU | 41653 | Crotalus | oreganus | concolor | USA | Utah | Grand |
| BYU | 41698 | Crotalus | viridis | nuntius | USA | Utah | San Juan |
| BYU | 41699 | Crotalus | viridis | nuntius | USA | Utah | San Juan |
| BYU | 41702 | Crotalus | oreganus | caliginis | Mexico | N. Baja California |  |
| BYU | 41746 | Crotalus | oreganus | concolor | USA | Utah | Kane |
| BYU | 42090 | Crotalus | oreganus | lutosus | USA | Utah | Kane |
| BYU | 42091 | Crotalus | oreganus | lutosus | USA | Utah | Kane |
| BYU | 42398 | Crotalus | oreganus | lutosus | USA | Utah | Utah |
| BYU | 42673 | Crotalus | oreganus | lutosus | USA | Utah | Utah |
| BYU | 42695 | Crotalus | oreganus | concolor | USA | Utah | Kane |
| BYU | 42702 | Crotalus | oreganus | concolor | USA | Utah |  |
| BYU | 42709 | Crotalus | oreganus | lutosus | USA | Utah | Washington |
| BYU | 42710 | Crotalus | oreganus | lutosus | USA | Arizona | Coconino |
| BYU | 42713 | Crotalus | viridis | nuntius | USA | Utah | San Juan |
| BYU | 42724 | Crotalus | oreganus | concolor | USA | Wyoming | Sweetwater |
| BYU | 42725 | Crotalus | oreganus | lutosus | USA | Utah | Cache |
| BYU | 42729 | Crotalus | oreganus | concolor | USA | Wyoming | Sweetwater |
| BYU | 42730 | Crotalus | unknown | unknown |  |  |  |
| BYU | 42732 | Crotalus | oreganus | lutosus | USA | Utah | Cache |
| BYU | 42841 | Crotalus | oreganus | concolor | USA | Wyoming | Sweetwater |
| BYU | 42842 | Crotalus | oreganus | concolor | USA | Wyoming | Sweetwater |
| BYU | 42844 | Crotalus | viridis | viridis | USA | Nebraska | Morrill |
| BYU | 42848 | Crotalus | oreganus | concolor | USA | Utah |  |
| BYU | 42851 | Crotalus | oreganus | concolor | USA | Utah |  |
| BYU | 42856 | Crotalus | oreganus | concolor | USA | Utah |  |
| BYU | 42857 | Crotalus | oreganus | lutosus | USA | Utah | Rich |
| BYU | 42860 | Crotalus | oreganus | concolor | USA | Utah |  |
| BYU | 42962 | Crotalus | oreganus | lutosus | USA | Arizona | Coconino |
| BYU | 43147 | Crotalus | oreganus | lutosus | USA | Utah | Utah |
| BYU | 43149 | Crotalus | oreganus | lutosus | USA | Utah | Washington |
| BYU | 43157 | Crotalus | oreganus | concolor | USA | Utah | Duchense |
| BYU | 43506 | Crotalus | oreganus | lutosus | USA | Utah | Iron |
| BYU | 43510 | Crotalus | oreganus | lutosus | USA | Utah | Box Elder |
| BYU | 43735 | Crotalus | oreganus | lutosus | USA | Utah |  |
| BYU | 45616 | Crotalus | unknown | unknown |  |  |  |
| BYU | 45617 | Crotalus | oreganus | concolor | USA | Utah | Uintah |
| BYU | 45618 | Crotalus | oreganus | concolor | USA | Utah | Uintah |
| BYU | 45619 | Crotalus | oreganus | concolor | USA | Utah | Uintah |
| BYU | 45620 | Crotalus | oreganus | concolor | USA | Utah | Uintah |
| BYU | 45621 | Crotalus | oreganus | concolor | USA | Utah | Uintah |
| BYU | 45626 | Crotalus | oreganus | concolor | USA | Utah | Uintah |
| BYU | 45627 | Crotalus | oreganus | concolor | USA | Utah | Uintah |
| BYU | 45628 | Crotalus | oreganus | concolor | USA | Utah | Uintah |
| BYU | 45926 | Crotalus | oreganus | concolor | USA | Utah | Uintah |
| BYU | 45927 | Crotalus | oreganus | concolor | USA | Utah | Uintah |
| BYU | 45928 | Crotalus | oreganus | concolor | USA | Utah | Uintah |
| BYU | 46087 | Crotalus | oreganus | concolor | USA | Utah | Uintah |
| BYU | 46117 | Crotalus | oreganus | lutosus | USA | Utah | Kane |
| BYU | 46118 | Crotalus | oreganus | lutosus | USA | Utah | Utah |
| BYU | 46158 | Crotalus | unknown | unknown |  |  |  |
| BYU | 46503 | Crotalus | unknown | unknown |  |  |  |
| BYU | 46504 | Crotalus | unknown | unknown |  |  |  |
| BYU | 46505 | Crotalus | unknown | unknown |  |  |  |
| BYU | 46511 | Crotalus | unknown | unknown |  |  |  |
| BYU | 46515 | Crotalus | oreganus | cerberus | USA | Arizona |  |
| BYU | 46517 | Crotalus | unknown | unknown |  |  |  |
| BYU | 46518 | Crotalus | unknown | unknown |  |  |  |
| BYU | 46552 | Crotalus | unknown | unknown |  |  |  |
| BYU | 46553 | Crotalus | unknown | unknown |  |  |  |
| BYU | 46640 | Crotalus | unknown | unknown |  |  |  |
| BYU | 46858 | Crotalus | oreganus | lutosus | USA | Utah | Millard |
| CAS | 1206 | Crotalus | oreganus | oreganus | USA | California | San Diego |
| CAS | 1207 | Crotalus | oreganus | oreganus | USA | California | San Diego |
| CAS | 1662 | Crotalus | oreganus | lutosus | USA | Idaho | Jerome |
| CAS | 1667 | Crotalus | oreganus | oreganus | USA | California | Tulare |
| CAS | 1670 | Crotalus | oreganus | oreganus | USA | California | Tulare |
| CAS | 1804 | Crotalus | oreganus | oreganus | USA | California | Riverside |
| CAS | 4027 | Crotalus | viridis | viridis | USA | Colorado |  |
| CAS | 4052 | Crotalus | oreganus | oreganus | USA | Idaho | Jerome |
| CAS | 4095 | Crotalus | oreganus | oreganus | USA | California | Monterey |
| CAS | 4158 | Crotalus | oreganus | oreganus | USA | California | Lake |
| CAS | 4405 | Crotalus | oreganus | oreganus | USA | California | San Bernardino |
| CAS | 5233 | Crotalus | oreganus | oreganus | USA | California | Tulare |
| CAS | 5280 | Crotalus | oreganus | oreganus | USA | California | Santa Clara |
| CAS | 6415 | Crotalus | viridis | viridis | USA | New Mexico | Bernalillo |
| CAS | 6419 | Crotalus | viridis | viridis | USA | New Mexico | Bernalillo |
| CAS | 6521 | Crotalus | oreganus | oreganus | USA | California | Santa Clara |
| CAS | 6542 | Crotalus | oreganus | lutosus | USA | Nevada | Washoe |
| CAS | 6681 | Crotalus | oreganus | caliginis | Mexico | Baja California Norte |  |
| CAS | 7234 | Crotalus | oreganus | lutosus | USA | Nevada | Washoe |
| CAS | 7690 | Crotalus | oreganus | oreganus | USA | California | Siskiyou |
| CAS | 7691 | Crotalus | oreganus | oreganus | USA | California |  |
| CAS | 8099 | Crotalus | oreganus | oreganus | USA | California | San Mateo |
| CAS | 8889 | Crotalus | oreganus | oreganus | USA | California | Tulare |
| CAS | 8890 | Crotalus | oreganus | oreganus | USA | California | Tulare |
| CAS | 8924 | Crotalus | oreganus | oreganus | USA | California | Tulare |
| CAS | 9781 | Crotalus | viridis | viridis | USA | Kansas | Hamilton |
| CAS | 9782 | Crotalus | viridis | viridis | USA | Kansas | Hamilton |
| CAS | 9783 | Crotalus | viridis | viridis | USA | Kansas | Logan |
| CAS | 9907 | Crotalus | viridis | viridis | USA | Kansas | Meade |
| CAS | 10419 | Crotalus | viridis | viridis | USA | Kansas | Ness |
| CAS | 10420 | Crotalus | viridis | viridis | USA | Kansas | Ness |
| CAS | 10421 | Crotalus | viridis | viridis | USA | Oklahoma | Beaver |
| CAS | 10422 | Crotalus | viridis | viridis | USA | Texas | Riverside |
| CAS | 10749 | Crotalus | oreganus | helleri | USA | California | San Diego |
| CAS | 10754 | Crotalus | oreganus | oreganus | USA | California | San Mateo |
| CAS | 10757 | Crotalus | oreganus | oreganus | USA | California | San Mateo |
| CAS | 10758 | Crotalus | oreganus | oreganus | USA | California | San Mateo |
| CAS | 10759 | Crotalus | oreganus | oreganus | USA | California | San Mateo |
| CAS | 10761 | Crotalus | oreganus | oreganus | USA | California | Humboldt |
| CAS | 10762 | Crotalus | oreganus | oreganus | USA | California | Humboldt |
| CAS | 10763 | Crotalus | oreganus | oreganus | USA | California | Humboldt |
| CAS | 10764 | Crotalus | oreganus | oreganus | USA | California | Humboldt |
| CAS | 10820 | Crotalus | viridis | nuntius | USA | Arizona | Apache |
| CAS | 10820 | Crotalus | viridis | nuntius | USA | Arizona |  |
| CAS | 10821 | Crotalus | oreganus | oreganus | USA | California | San Benito |
| CAS | 10822 | Crotalus | oreganus | oreganus | USA | California | Tuolumne |
| CAS | 10886 | Crotalus | viridis | viridis | USA | New Mexico | Catron |
| CAS | 10901 | Crotalus | oreganus | oreganus | USA | California | Humboldt |
| CAS | 10902 | Crotalus | oreganus | oreganus | USA | California | Tuolumne |
| CAS | 10940 | Crotalus | oreganus | oreganus | USA | California | Santa Clara |
| CAS | 11447 | Crotalus | oreganus | oreganus | USA | California | San Mateo |
| CAS | 11574 | Crotalus | oreganus | helleri | Mexico | Baja California Norte |  |
| CAS | 11613 | Crotalus | viridis | viridis | USA | Wyoming | Park |
| CAS | 11919 | Crotalus | oreganus | oreganus | USA | California | Tuolumne |
| CAS | 12141 | Crotalus | oreganus | helleri | Mexico | Baja California Norte |  |
| CAS | 12142 | Crotalus | oreganus | helleri | Mexico | Baja California Norte |  |
| CAS | 13452 | Crotalus | oreganus | helleri | USA | California | Los Angeles |
| CAS | 13476 | Crotalus | oreganus | caliginis | Mexico | Baja California Norte |  |
| CAS | 13583 | Crotalus | oreganus | caliginis | Mexico | Baja California Norte |  |
| CAS | 13584 | Crotalus | oreganus | caliginis | Mexico | Baja California Norte |  |
| CAS | 13585 | Crotalus | oreganus | caliginis | Mexico | Baja California Norte |  |
| CAS | 13586 | Crotalus | oreganus | caliginis | Mexico | Baja California Norte |  |
| CAS | 13587 | Crotalus | oreganus | caliginis | Mexico | Baja California Norte |  |
| CAS | 13603 | Crotalus | oreganus | caliginis | Mexico | Baja California Norte |  |
| CAS | 13639 | Crotalus | oreganus | oreganus | USA | California |  |
| CAS | 14361 | Crotalus | viridis | viridis | Mexico | Chihuahua |  |
| CAS | 14367 | Crotalus | oreganus | lutosus | USA | Nevada | White Pine |
| CAS | 15119 | Crotalus | oreganus | lutosus | USA | Nevada | Mineral |
| CAS | 17531 | Crotalus | oreganus | cerberus | USA | Arizona | Maricopa |
| CAS | 17539 | Crotalus | oreganus | cerberus | USA | Arizona | Maricopa |
| CAS | 17915 | Crotalus | oreganus | oreganus | USA | California | San Mateo |
| CAS | 19095 | Crotalus | oreganus | oreganus | USA | California | Tuolumne |
| CAS | 19096 | Crotalus | oreganus | oreganus | USA | California | Tuolumne |
| CAS | 19827 | Crotalus | oreganus | helleri | USA | California | Los Angeles |
| CAS | 19871 | Crotalus | oreganus | oreganus | USA | California | San Mateo |
| CAS | 19872 | Crotalus | oreganus | oreganus | USA | California | San Mateo |
| CAS | 19914 | Crotalus | oreganus | lutosus | USA | Nevada | Washoe |
| CAS | 19915 | Crotalus | oreganus | lutosus | USA | Nevada | Washoe |
| CAS | 19916 | Crotalus | oreganus | lutosus | USA | Nevada | Carson City |
| CAS | 21619 | Crotalus | oreganus | lutosus | USA | Nevada | Washoe |
| CAS | 21620 | Crotalus | oreganus | lutosus | USA | Nevada | Washoe |
| CAS | 21622 | Crotalus | oreganus | lutosus | USA | Nevada |  |
| CAS | 21623 | Crotalus | oreganus | lutosus | USA | Nevada |  |
| CAS | 21624 | Crotalus | oreganus | lutosus | USA | Nevada |  |
| CAS | 21625 | Crotalus | oreganus | lutosus | USA | Nevada |  |
| CAS | 21626 | Crotalus | oreganus | lutosus | USA | Nevada |  |
| CAS | 21627 | Crotalus | oreganus | lutosus | USA | Nevada |  |
| CAS | 21628 | Crotalus | oreganus | lutosus | USA | Nevada |  |
| CAS | 21629 | Crotalus | oreganus | lutosus | USA | Nevada |  |
| CAS | 21630 | Crotalus | oreganus | lutosus | USA | Nevada |  |
| CAS | 21631 | Crotalus | oreganus | lutosus | USA | Nevada | Washoe |
| CAS | 21632 | Crotalus | oreganus | lutosus | USA | Nevada | Washoe |
| CAS | 21633 | Crotalus | oreganus | lutosus | USA | Nevada | Washoe |
| CAS | 21634 | Crotalus | oreganus | lutosus | USA | Nevada | Washoe |
| CAS | 21635 | Crotalus | oreganus | lutosus | USA | Nevada | Washoe |
| CAS | 21636 | Crotalus | oreganus | lutosus | USA | Nevada | Washoe |
| CAS | 21637 | Crotalus | oreganus | lutosus | USA | Nevada | Washoe |
| CAS | 21638 | Crotalus | oreganus | lutosus | USA | Nevada | Washoe |
| CAS | 21639 | Crotalus | oreganus | lutosus | USA | Nevada | Washoe |
| CAS | 21641 | Crotalus | oreganus | lutosus | USA | Nevada | Washoe |
| CAS | 21642 | Crotalus | oreganus | lutosus | USA | Nevada | Washoe |
| CAS | 21643 | Crotalus | oreganus | lutosus | USA | Nevada | Washoe |
| CAS | 21645 | Crotalus | oreganus | lutosus | USA | Nevada | Washoe |
| CAS | 21646 | Crotalus | oreganus | lutosus | USA | Nevada | Washoe |
| CAS | 21661 | Crotalus | oreganus | lutosus | USA | Utah | Utah |
| CAS | 21766 | Crotalus | oreganus | oreganus | USA |  |  |
| CAS | 22969 | Crotalus | oreganus | oreganus | USA | California | San Mateo |
| CAS | 22971 | Crotalus | oreganus | oreganus | USA | California | San Luis Obispo |
| CAS | 22974 | Crotalus | oreganus | oreganus | USA | California | Santa Clara |
| CAS | 22975 | Crotalus | oreganus | oreganus | USA | California | San Mateo |
| CAS | 22976 | Crotalus | oreganus | oreganus | USA | California | San Mateo |
| CAS | 23087 | Crotalus | oreganus | oreganus |  |  |  |
| CAS | 23215 | Crotalus | oreganus | oreganus | USA | California | Fresno |
| CAS | 23216 | Crotalus | oreganus | oreganus | USA | California | San Benito |
| CAS | 27196 | Crotalus | oreganus | lutosus | USA | Utah | Salt Lake |
| CAS | 27277 | Crotalus | oreganus | helleri | USA | California | San Diego |
| CAS | 27278 | Crotalus | oreganus | helleri | USA | California | San Diego |
| CAS | 30921 | Crotalus | oreganus | lutosus | USA | Utah | Salt Lake |
| CAS | 30922 | Crotalus | oreganus | lutosus | USA | Utah | Salt Lake |
| CAS | 31621 | Crotalus | oreganus | lutosus |  |  |  |
| CAS | 31640 | Crotalus | oreganus | lutosus |  |  |  |
| CAS | 34683 | Crotalus | oreganus | cerberus | USA | Arizona | Pima |
| CAS | 35237 | Crotalus | oreganus | cerberus | USA | Arizona | Coconino |
| CAS | 36064 | Crotalus | oreganus | oreganus | USA | California | Siskiyou |
| CAS | 37997 | Crotalus | oreganus | lutosus | USA | Nevada | Elko |
| CAS | 38098 | Crotalus | oreganus | concolor | USA | Utah | Grand |
| CAS | 38439 | Crotalus | oreganus | lutosus | USA | Utah | Wasatch |
| CAS | 38954 | Crotalus | oreganus | oreganus | USA | California | Kern |
| CAS | 38955 | Crotalus | oreganus | oreganus | USA | California | Kern |
| CAS | 38956 | Crotalus | oreganus | oreganus | USA | California | Kern |
| CAS | 38956 | Crotalus | oreganus | oreganus | USA | California | Kern |
| CAS | 38957 | Crotalus | oreganus | oreganus | USA | California | Kern |
| CAS | 39645 | Crotalus | oreganus | oreganus | USA | California | El Dorado |
| CAS | 40051 | Crotalus | oreganus | helleri | USA | California | San Diego |
| CAS | 40052 | Crotalus | oreganus | helleri | USA | California | San Diego |
| CAS | 40053 | Crotalus | oreganus | helleri | USA | California | San Diego |
| CAS | 40055 | Crotalus | oreganus | helleri | USA | California | San Diego |
| CAS | 40056 | Crotalus | oreganus | helleri | USA | California | San Diego |
| CAS | 40083 | Crotalus | oreganus | helleri | USA | California | San Diego |
| CAS | 40084 | Crotalus | oreganus | helleri | USA | California | San Diego |
| CAS | 40086 | Crotalus | oreganus | helleri | USA | California | San Diego |
| CAS | 40087 | Crotalus | oreganus | helleri | USA | California | San Diego |
| CAS | 40088 | Crotalus | oreganus | helleri | USA | California | San Diego |
| CAS | 40089 | Crotalus | oreganus | helleri | USA | California | San Diego |
| CAS | 40960 | Crotalus | oreganus | concolor | USA | Utah | Grand |
| CAS | 43420 | Crotalus | oreganus | oreganus | USA | California | Grand |
| CAS | 43426 | Crotalus | oreganus | oreganus | USA | California | San Luis Obispo |
| CAS | 43529 | Crotalus | oreganus | oreganus | USA | California | Kern |
| CAS | 43559 | Crotalus | oreganus | oreganus | USA | California | Contra Costa |
| CAS | 44220 | Crotalus | oreganus | lutosus | USA | California | Lassen |
| CAS | 44221 | Crotalus | oreganus | lutosus | USA | California | Lassen |
| CAS | 47751 | Crotalus | oreganus | lutosus | USA | Utah | Millard |
| CAS | 47752 | Crotalus | oreganus | lutosus | USA | Utah | Washington |
| CAS | 49232 | Crotalus | oreganus | oreganus | USA | California | Santa Clara |
| CAS | 50054 | Crotalus | oreganus | helleri | USA | California | San Diego |
| CAS | 50171 | Crotalus | oreganus | helleri | USA | California | Los Angeles |
| CAS | 50171 | Crotalus | oreganus | helleri | USA | California | Los Angeles |
| CAS | 50346 | Crotalus | oreganus | helleri | USA | California | Ventura |
| CAS | 50354 | Crotalus | oreganus | helleri | USA | California | Ventura |
| CAS | 50355 | Crotalus | oreganus | helleri | USA | California | Ventura |
| CAS | 54011 | Crotalus | oreganus | lutosus | USA | Utah | Beaver |
| CAS | 55223 | Crotalus | oreganus | lutosus | USA | Idaho |  |
| CAS | 55224 | Crotalus | oreganus | lutosus | USA | Idaho |  |
| CAS | 55225 | Crotalus | oreganus | lutosus | USA | Idaho |  |
| CAS | 55226 | Crotalus | oreganus | lutosus | USA | Idaho |  |
| CAS | 55227 | Crotalus | oreganus | lutosus | USA | Idaho |  |
| CAS | 55233 | Crotalus | oreganus | lutosus | USA | Idaho |  |
| CAS | 56864 | Crotalus | oreganus | helleri | Mexico | Baja California Norte |  |
| CAS | 56883 | Crotalus | oreganus | helleri | Mexico | Baja California Norte |  |
| CAS | 56883 | Crotalus | oreganus | helleri | Mexico | Baja California Norte |  |
| CAS | 57555 | Crotalus | oreganus | helleri | Mexico | Baja California Norte |  |
| CAS | 58119 | Crotalus | oreganus | helleri | USA | California | San Diego |
| CAS | 58142 | Crotalus | oreganus | helleri | USA | California | San Diego |
| CAS | 62649 | Crotalus | viridis | viridis | USA | Kansas | Ness |
| CAS | 62985 | Crotalus | oreganus | helleri | USA | California | San Diego |
| CAS | 62986 | Crotalus | oreganus | helleri | USA | California | San Diego |
| CAS | 62987 | Crotalus | oreganus | helleri | USA | California | San Diego |
| CAS | 63897 | Crotalus | viridis | nuntius | USA | Arizona | Coconino |
| CAS | 63898 | Crotalus | viridis | nuntius | USA | Arizona | Coconino |
| CAS | 63899 | Crotalus | viridis | nuntius | USA | Arizona | Apache |
| CAS | 63900 | Crotalus | viridis | nuntius | USA | Arizona | Apache |
| CAS | 63902 | Crotalus | viridis | nuntius | USA | Arizona | Coconino |
| CAS | 63903 | Crotalus | viridis | nuntius | USA | Arizona | Coconino |
| CAS | 63904 | Crotalus | viridis | nuntius | USA | Arizona | Coconino |
| CAS | 63905 | Crotalus | viridis | nuntius | USA | Arizona | Coconino |
| CAS | 63906 | Crotalus | oreganus | abyssus | USA | Arizona | Coconino |
| CAS | 63907 | Crotalus | viridis | nuntius | USA | Arizona | Navajo |
| CAS | 63908 | Crotalus | oreganus | lutosus | USA | Idaho | Ada |
| CAS | 63909 | Crotalus | oreganus | lutosus | USA | Idaho | Ada |
| CAS | 64120 | Crotalus | oreganus | lutosus | USA | Idaho | Ada |
| CAS | 64121 | Crotalus | oreganus | lutosus | USA | Idaho | Ada |
| CAS | 64122 | Crotalus | oreganus | lutosus | USA | Idaho | Ada |
| CAS | 64127 | Crotalus | oreganus | lutosus | USA | Idaho | Ada |
| CAS | 64191 | Crotalus | oreganus | lutosus | USA | Idaho | Ada |
| CAS | 64192 | Crotalus | oreganus | lutosus | USA | Idaho | Ada |
| CAS | 64193 | Crotalus | oreganus | lutosus | USA | Idaho | Ada |
| CAS | 64194 | Crotalus | oreganus | lutosus | USA | Idaho | Ada |
| CAS | 64195 | Crotalus | oreganus | lutosus | USA | Idaho | Ada |
| CAS | 64196 | Crotalus | oreganus | lutosus | USA | Idaho | Ada |
| CAS | 64208 | Crotalus | oreganus | lutosus | USA | Idaho | Elmore |
| CAS | 64209 | Crotalus | oreganus | lutosus | USA | Nevada | Washoe |
| CAS | 64210 | Crotalus | oreganus | lutosus | USA | Nevada | Washoe |
| CAS | 64580 | Crotalus | oreganus | helleri | USA | California | San Diego |
| CAS | 64581 | Crotalus | oreganus | helleri | USA | California | San Diego |
| CAS | 64582 | Crotalus | oreganus | helleri | USA | California | San Diego |
| CAS | 64583 | Crotalus | oreganus | helleri | USA | California | San Diego |
| CAS | 64584 | Crotalus | oreganus | helleri | USA | California | San Diego |
| CAS | 64585 | Crotalus | oreganus | helleri | USA | California | San Diego |
| CAS | 64586 | Crotalus | oreganus | helleri | USA | California | San Diego |
| CAS | 65093 | Crotalus | viridis | viridis | USA | Colorado | Weld |
| CAS | 65094 | Crotalus | viridis | viridis | USA | Colorado | Weld |
| CAS | 65095 | Crotalus | viridis | viridis | USA | Colorado | Weld |
| CAS | 65096 | Crotalus | viridis | viridis | USA | Colorado | Weld |
| CAS | 65097 | Crotalus | viridis | viridis | USA | Colorado | Weld |
| CAS | 65098 | Crotalus | viridis | viridis | USA | Colorado | Weld |
| CAS | 65099 | Crotalus | viridis | viridis | USA | Colorado | Weld |
| CAS | 65100 | Crotalus | viridis | viridis | USA | Colorado | Weld |
| CAS | 65101 | Crotalus | viridis | viridis | USA | Colorado | Weld |
| CAS | 65102 | Crotalus | viridis | viridis | USA | Colorado | Weld |
| CAS | 65103 | Crotalus | viridis | viridis | USA | Colorado | Weld |
| CAS | 65104 | Crotalus | viridis | viridis | USA | Colorado | Weld |
| CAS | 65105 | Crotalus | viridis | viridis | USA | Colorado | Weld |
| CAS | 65106 | Crotalus | viridis | viridis | USA | Colorado | Weld |
| CAS | 65107 | Crotalus | viridis | viridis | USA | Colorado | Weld |
| CAS | 65108 | Crotalus | viridis | viridis | USA | Colorado | Weld |
| CAS | 65109 | Crotalus | viridis | viridis | USA | Colorado | Weld |
| CAS | 65724 | Crotalus | viridis | viridis | USA | Colorado | Weld |
| CAS | 65725 | Crotalus | viridis | viridis | USA | Colorado | Weld |
| CAS | 65726 | Crotalus | viridis | viridis | USA | Colorado | Weld |
| CAS | 65727 | Crotalus | viridis | viridis | USA | Colorado | Weld |
| CAS | 65728 | Crotalus | viridis | viridis | USA | Colorado | Weld |
| CAS | 65912 | Crotalus | oreganus | lutosus | USA | Utah | Millard |
| CAS | 65923 | Crotalus | viridis | viridis | USA | Colorado | Weld |
| CAS | 66389 | Crotalus | viridis | viridis | USA | New Mexico | Santa Fe |
| CAS | 71332 | Crotalus | oreganus | oreganus | USA | California | Santa Cruz |
| CAS | 78687 | Crotalus | oreganus | oreganus | USA | California | Sacramento |
| CAS | 78696 | Crotalus | oreganus | oreganus | USA | California | Trinity |
| CAS | 78697 | Crotalus | oreganus | oreganus | USA | California | Santa Clara |
| CAS | 78701 | Crotalus | oreganus | oreganus | USA | California | Sonoma |
| CAS | 78705 | Crotalus | oreganus | oreganus | USA | California | Sonoma |
| CAS | 78706 | Crotalus | oreganus | oreganus | USA | California | Sonoma |
| CAS | 80919 | Crotalus | oreganus | oreganus | USA | California | Sonoma |
| CAS | 81530 | Crotalus | oreganus | oreganus | USA | California | Santa Cruz |
| CAS | 81530 | Crotalus | oreganus | oreganus | USA | California | Santa Cruz |
| CAS | 84148 | Crotalus | oreganus | oreganus | USA | California | Tuolumne |
| CAS | 85130 | Crotalus | oreganus | helleri | Mexico | Baja California Norte |  |
| CAS | 91623 | Crotalus | oreganus | lutosus | USA | Nevada | Washoe |
| CAS | 91624 | Crotalus | viridis | lutosus | USA | Nevada | Washoe |
| CAS | 91630 | Crotalus | oreganus | oreganus | USA | California | San Benito |
| CAS | 92263 | Crotalus | oreganus | lutosus | USA | Nevada | Washoe |
| CAS | 93785 | Crotalus | oreganus | lutosus | USA | Nevada | Washoe |
| CAS | 93787 | Crotalus | oreganus | lutosus | USA | California | Santa Cruz |
| CAS | 93788 | Crotalus | oreganus | lutosus | USA | Nevada | Washoe |
| CAS | 93790 | Crotalus | oreganus | lutosus | USA | Nevada | Washoe |
| CAS | 93791 | Crotalus | oreganus | lutosus | USA | Nevada | Washoe |
| CAS | 93792 | Crotalus | oreganus | lutosus | USA | Nevada | Washoe |
| CAS | 93794 | Crotalus | viridis | viridis | USA | Colorado | El Paso |
| CAS | 93795 | Crotalus | viridis | viridis | USA | Colorado | El Paso |
| CAS | 93796 | Crotalus | viridis | viridis | USA | Colorado | El Paso |
| CAS | 93797 | Crotalus | viridis | viridis | USA | Colorado | El Paso |
| CAS | 93798 | Crotalus | viridis | viridis | USA | Colorado | El Paso |
| CAS | 93798 | Crotalus | viridis | viridis | USA | Colorado | El Paso |
| CAS | 93799 | Crotalus | viridis | viridis | USA | Colorado | El Paso |
| CAS | 93800 | Crotalus | viridis | viridis | USA | Colorado | El Paso |
| CAS | 93801 | Crotalus | viridis | viridis | USA | Colorado | El Paso |
| CAS | 93802 | Crotalus | viridis | viridis | USA | Colorado | El Paso |
| CAS | 93803 | Crotalus | viridis | viridis | USA | Colorado | El Paso |
| CAS | 98551 | Crotalus | oreganus | lutosus | USA | Nevada | Washoe |
| CAS | 100831 | Crotalus | viridis | viridis | USA | Colorado | El Paso |
| CAS | 100832 | Crotalus | viridis | viridis | USA | Colorado | El Paso |
| CAS | 100833 | Crotalus | viridis | viridis | USA | Colorado | El Paso |
| CAS | 100834 | Crotalus | viridis | viridis | USA | Colorado | El Paso |
| CAS | 100835 | Crotalus | viridis | viridis | USA | Colorado | El Paso |
| CAS | 100836 | Crotalus | viridis | viridis | USA | Colorado | El Paso |
| CAS | 100837 | Crotalus | viridis | viridis | USA | Colorado | El Paso |
| CAS | 100838 | Crotalus | viridis | viridis | USA | Colorado | El Paso |
| CAS | 100839 | Crotalus | viridis | viridis | USA | Colorado | El Paso |
| CAS | 100840 | Crotalus | viridis | viridis | USA | Colorado | El Paso |
| CAS | 100841 | Crotalus | viridis | viridis | USA | Colorado | El Paso |
| CAS | 100842 | Crotalus | viridis | viridis | USA | Colorado | El Paso |
| CAS | 100843 | Crotalus | viridis | viridis | USA | Colorado | El Paso |
| CAS | 102311 | Crotalus | viridis | viridis | USA | Colorado | El Paso |
| CAS | 102312 | Crotalus | viridis | viridis | USA | Colorado | El Paso |
| CAS | 102313 | Crotalus | viridis | viridis | USA | Colorado | El Paso |
| CAS | 102521 | Crotalus | unknown | unknown |  |  |  |
| CAS | 102915 | Crotalus | oreganus | lutosus | USA | Nevada | Mineral |
| CAS | 103050 | Crotalus | oreganus | concolor | USA | Utah | Grand |
| CAS | 107454 | Crotalus | viridis | viridis | USA | Colorado | Moffat |
| CAS | 125419 | Crotalus | oreganus | lutosus | USA | Nevada | Washoe |
| CAS | 148648 | Crotalus | oreganus | concolor | USA | Utah | Butte |
| CAS | 152497 | Crotalus | oreganus | oreganus | USA | California | Butte |
| CAS | 152498 | Crotalus | oreganus | oreganus | USA | California | Siskiyou |
| CAS | 152510 | Crotalus | oreganus | oreganus | USA | California | Yolo |
| CAS | 156187 | Crotalus | viridis | nuntius | USA | Arizona | Coconino |
| CAS | 160112 | Crotalus | oreganus | oreganus | USA | California | Alameda |
| CAS | 160113 | Crotalus | oreganus | oreganus | USA | California | Alameda |
| CAS | 161912 | Crotalus | oreganus | oreganus | USA | California | Alameda |
| CAS | 161979 | Crotalus | oreganus | oreganus | USA | California | Stanislaus |
| CAS | 162689 | Crotalus | oreganus | oreganus | USA | California | San Joaquin |
| CAS | 165194 | Crotalus | oreganus | oreganus | USA | California | Alameda |
| CAS | 169497 | Crotalus | oreganus | oreganus | USA | California | Sonoma |
| CAS | 170409 | Crotalus | oreganus | concolor | USA | Utah | San Juan |
| CAS | 170410 | Crotalus | oreganus | concolor | USA | Utah | San Juan |
| CAS | 170411 | Crotalus | oreganus | concolor | USA | Utah | San Juan |
| CAS | 170412 | Crotalus | oreganus | concolor | USA | Utah | San Juan |
| CAS | 170413 | Crotalus | oreganus | helleri | USA | California | San Diego |
| CAS | 170414 | Crotalus | oreganus | helleri | USA | California | San Diego |
| CAS | 170415 | Crotalus | oreganus | lutosus | USA | Utah | Salt Lake |
| CAS | 170416 | Crotalus | oreganus | concolor | USA | Utah | San Juan |
| CAS | 170418 | Crotalus | oreganus | helleri | USA | California | San Diego |
| CAS | 170419 | Crotalus | viridis | viridis | USA | New Mexico | Bernalillo |
| CAS | 170420 | Crotalus | viridis | viridis | USA | Colorado | Baca |
| CAS | 170420 | Crotalus | viridis | viridis | USA | Colorado | Baca |
| CAS | 170421 | Crotalus | oreganus | cerberus | USA | Arizona | Pima |
| CAS | 170422 | Crotalus | oreganus | helleri | USA | California | San Diego |
| CAS | 170423 | Crotalus | oreganus | helleri | USA | California | San Diego |
| CAS | 170424 | Crotalus | oreganus | helleri | USA | California | San Diego |
| CAS | 170425 | Crotalus | oreganus | helleri | USA | California | San Diego |
| CAS | 170426 | Crotalus | oreganus | concolor | USA | Utah | San Juan |
| CAS | 170427 | Crotalus | oreganus | lutosus | USA | Utah | Utah |
| CAS | 170428 | Crotalus | oreganus | lutosus | USA | Utah | Utah |
| CAS | 170429 | Crotalus | oreganus | lutosus | USA | Utah | Utah |
| CAS | 170430 | Crotalus | oreganus | oreganus | USA | Washington | Kittitas |
| CAS | 170431 | Crotalus | oreganus | oreganus | USA | Washington | Kittitas |
| CAS | 170432 | Crotalus | oreganus | oreganus | USA | Washington | Kittitas |
| CAS | 170433 | Crotalus | viridis | viridis | USA | New Mexico | Bernalillo |
| CAS | 170434 | Crotalus | oreganus | concolor | USA | Utah | San Juan |
| CAS | 170436 | Crotalus | oreganus | oreganus | USA | Washington | Kittitas |
| CAS | 170437 | Crotalus | oreganus | oreganus | USA | Washington | Kittitas |
| CAS | 170438 | Crotalus | viridis | viridis | USA | Texas | Randall |
| CAS | 170439 | Crotalus | oreganus | oreganus | USA | Washington | Kittitas |
| CAS | 170440 | Crotalus | viridis | viridis | USA | New Mexico | Valencia |
| CAS | 170441 | Crotalus | viridis | viridis | USA | New Mexico | Santa Fe |
| CAS | 170443 | Crotalus | viridis | viridis | USA | Colorado | Weld |
| CAS | 170444 | Crotalus | viridis | nuntius | USA | Arizona | Navajo |
| CAS | 170445 | Crotalus | viridis | viridis | USA | Colorado | Weld |
| CAS | 170446 | Crotalus | viridis | viridis | USA | Colorado | Weld |
| CAS | 170447 | Crotalus | viridis | viridis | USA | New Mexico | Hidalgo |
| CAS | 170448 | Crotalus | viridis | viridis | USA | New Mexico | Sandoval |
| CAS | 170448 | Crotalus | viridis | viridis | USA | New Mexico | Sandoval |
| CAS | 170449 | Crotalus | viridis | viridis | USA | New Mexico | Bernillilo |
| CAS | 170453 | Crotalus | viridis | viridis | USA | Colorado | Moffat |
| CAS | 170455 | Crotalus | viridis | viridis | USA | Colorado | Moffat |
| CAS | 170456 | Crotalus | viridis | viridis | USA | Colorado | Moffat |
| CAS | 170457 | Crotalus | viridis | viridis | USA | Colorado | Moffat |
| CAS | 170458 | Crotalus | viridis | viridis | USA | Colorado | Moffat |
| CAS | 170459 | Crotalus | oreganus | concolor | USA | Wyoming | Sweetwater |
| CAS | 170460 | Crotalus | oreganus | concolor | USA | Wyoming | Sweetwater |
| CAS | 170461 | Crotalus | oreganus | concolor | USA | Wyoming | Sweetwater |
| CAS | 170462 | Crotalus | oreganus | concolor | USA | Utah | Utah |
| CAS | 170463 | Crotalus | oreganus | concolor | USA | Wyoming | Sweetwater |
| CAS | 170464 | Crotalus | oreganus | concolor | USA | Wyoming | Sweetwater |
| CAS | 170465 | Crotalus | oreganus | concolor | USA | Wyoming | Sweetwater |
| CAS | 170466 | Crotalus | viridis | viridis | USA | New Mexico | McKinley |
| CAS | 170467 | Crotalus | viridis | viridis | USA | New Mexico | McKinley |
| CAS | 170469 | Crotalus | viridis | viridis | USA | New Mexico | McKinley |
| CAS | 170469 | Crotalus | viridis | viridis | USA | New Mexico | McKinley |
| CAS | 170469 | Crotalus | viridis | viridis | USA | New Mexico | McKinley |
| CAS | 170470 | Crotalus | viridis | viridis | USA | Wyoming | Fremont |
| CAS | 170471 | Crotalus | oreganus | oreganus | USA | Idaho | Nez Perce |
| CAS | 170471 | Crotalus | oreganus | oreganus | USA | Idaho | Nez Perce |
| CAS | 170472 | Crotalus | viridis | viridis | USA | Montana | Wheatland |
| CAS | 170473 | Crotalus | viridis | viridis | USA | Colorado | Baca |
| CAS | 170474 | Crotalus | viridis | viridis | USA | Colorado | Costilla |
| CAS | 170475 | Crotalus | viridis | viridis | USA | Colorado | Costilla |
| CAS | 170476 | Crotalus | viridis | viridis | USA | Colorado | Costilla |
| CAS | 170477 | Crotalus | viridis | viridis | USA | Wyoming | Park |
| CAS | 170478 | Crotalus | oreganus | lutosus | USA | Utah | Salt Lake |
| CAS | 170479 | Crotalus | oreganus | lutosus | USA | Utah | Salt Lake |
| CAS | 170480 | Crotalus | oreganus | lutosus | USA | Utah | Salt Lake |
| CAS | 170481 | Crotalus | oreganus | lutosus | USA | Utah | Salt Lake |
| CAS | 170482 | Crotalus | viridis | viridis | USA | Montana | Wheatland |
| CAS | 170483 | Crotalus | viridis | viridis | USA | Montana | Wheatland |
| CAS | 170484 | Crotalus | viridis | viridis | USA | New Mexico | McKinley |
| CAS | 170485 | Crotalus | viridis | viridis | USA | New Mexico | McKinley |
| CAS | 170486 | Crotalus | viridis | viridis | USA | New Mexico | McKinley |
| CAS | 170487 | Crotalus | viridis | viridis | USA | Colorado | Baca |
| CAS | 170489 | Crotalus | viridis | viridis | USA | Colorado | Baca |
| CAS | 170490 | Crotalus | oreganus | oreganus | USA | Idaho | Nez Perce |
| CAS | 170491 | Crotalus | oreganus | oreganus | USA | Idaho | Nez Perce |
| CAS | 170492 | Crotalus | oreganus | oreganus | USA | Idaho | Nez Perce |
| CAS | 170493 | Crotalus | oreganus | oreganus | USA | Idaho | Nez Perce |
| CAS | 170494 | Crotalus | viridis | viridis | USA | Montana | Wheatland |
| CAS | 170495 | Crotalus | viridis | viridis | USA | New Mexico | McKinley |
| CAS | 170497 | Crotalus | viridis | viridis | USA | Colorado | Costilla |
| CAS | 170498 | Crotalus | oreganus | helleri | USA | California | San Bernardino |
| CAS | 170499 | Crotalus | oreganus | helleri | USA | California | San Bernardino |
| CAS | 170500 | Crotalus | oreganus | concolor | USA | Wyoming | Sweetwater |
| CAS | 170501 | Crotalus | viridis | viridis | USA | New Mexico | McKinley |
| CAS | 170503 | Crotalus | viridis | nuntius | USA | Arizona | Coconino |
| CAS | 170504 | Crotalus | oreganus | cerberus | USA | Arizona | Pima |
| CAS | 170507 | Crotalus | oreganus | cerberus | USA | Arizona | Mohave |
| CAS | 170508 | Crotalus | oreganus | cerberus | USA | Arizona | Graham |
| CAS | 170509 | Crotalus | viridis | nuntius | USA | Arizona | Coconino |
| CAS | 170510 | Crotalus | oreganus | cerberus | USA | Arizona | Pima |
| CAS | 170511 | Crotalus | viridis | nuntius | USA | Arizona | Apache |
| CAS | 170512 | Crotalus | oreganus | lutosus | USA | Utah | Salt Lake |
| CAS | 170513 | Crotalus | oreganus | lutosus | USA | Utah | Salt Lake |
| CAS | 170514 | Crotalus | viridis | viridis | USA | New Mexico | Socorro |
| CAS | 170515 | Crotalus | viridis | viridis | USA | New Mexico | Socorro |
| CAS | 170516 | Crotalus | viridis | viridis | USA | New Mexico | Socorro |
| CAS | 170517 | Crotalus | oreganus | cerberus | USA | Arizona | Mohave |
| CAS | 170518 | Crotalus | oreganus | cerberus | USA | Arizona | Graham |
| CAS | 170522 | Crotalus | oreganus | oreganus | USA | California | Santa Clara |
| CAS | 170524 | Crotalus | viridis | viridis | USA | South Dakota |  |
| CAS | 172070 | Crotalus | viridis | viridis | USA | Colorado |  |
| CAS | 172071 | Crotalus | oreganus | oreganus | USA | Idaho | Nez Perce |
| CAS | 172072 | Crotalus | viridis | viridis | USA | Colorado |  |
| CAS | 172073 | Crotalus | viridis | viridis | USA | Colorado |  |
| CAS | 172077 | Crotalus | viridis | viridis | USA | Colorado |  |
| CAS | 173568 | Crotalus | oreganus | oreganus | USA | California | San Joaquin |
| CAS | 176612 | Crotalus | oreganus | helleri | USA | California | San Diego |
| CAS | 178649 | Crotalus | oreganus | oreganus | USA | California | El Dorado |
| CAS | 178901 | Crotalus | oreganus | helleri | USA | California | San Diego |
| CAS | 178942 | Crotalus | oreganus | helleri | USA | California | San Diego |
| CAS | 178943 | Crotalus | oreganus | helleri | USA | California | San Diego |
| CAS | 178944 | Crotalus | oreganus | helleri | USA | California | San Diego |
| CAS | 180329 | Crotalus | oreganus | oreganus | USA | California | Kern |
| CAS | 182512 | Crotalus | oreganus | helleri | Mexico | Baja California Norte |  |
| CAS | 182512 | Crotalus | oreganus | helleri | Mexico | Baja California Norte |  |
| CAS | 182514 | Crotalus | oreganus | helleri | USA | California | San Diego |
| CAS | 182515 | Crotalus | oreganus | helleri | USA | California | San Diego |
| CAS | 182516 | Crotalus | oreganus | helleri | USA | California | San Diego |
| CAS | 182517 | Crotalus | oreganus | helleri | USA | California | San Diego |
| CAS | 182518 | Crotalus | oreganus | helleri | USA | California | San Diego |
| CAS | 182519 | Crotalus | oreganus | helleri | USA | California | San Diego |
| CAS | 182520 | Crotalus | oreganus | helleri | USA | California | San Diego |
| CAS | 182522 | Crotalus | oreganus | helleri | USA | California | San Diego |
| CAS | 182523 | Crotalus | oreganus | helleri | USA | California | San Diego |
| CAS | 182524 | Crotalus | oreganus | helleri | USA | California | San Diego |
| CAS | 182525 | Crotalus | oreganus | helleri | USA | California | San Diego |
| CAS | 182526 | Crotalus | oreganus | helleri | USA | California | San Diego |
| CAS | 182527 | Crotalus | oreganus | helleri | USA | California | San Diego |
| CAS | 182528 | Crotalus | oreganus | helleri | USA | California | San Diego |
| CAS | 182529 | Crotalus | oreganus | helleri | USA | California | San Diego |
| CAS | 182530 | Crotalus | oreganus | helleri | USA | California | San Diego |
| CAS | 182534 | Crotalus | oreganus | unknown | USA | California | Santa Barbara |
| CAS | 182537 | Crotalus | oreganus | unknown | USA | California | Santa Barbara |
| CAS | 182539 | Crotalus | oreganus | unknown | USA | California | Santa Barbara |
| CAS | 182540 | Crotalus | oreganus | unknown | USA | California | Santa Barbara |
| CAS | 182545 | Crotalus | oreganus | oreganus | USA | California | Santa Barbara |
| CAS | 182545 | Crotalus | oreganus | unknown | USA | California | Santa Barbara |
| CAS | 182584 | Crotalus | oreganus | unknown | USA | California | Ventura |
| CAS | 191129 | Crotalus | oreganus | oreganus | USA | California | Fresno |
| CAS | 192617 | Crotalus | oreganus | oreganus | USA | California | San Benito |
| CAS | 192619 | Crotalus | oreganus | oreganus | USA | California | San Benito |
| CAS | 192625 | Crotalus | oreganus | oreganus | USA | California | San Benito |
| CAS | 192629 | Crotalus | oreganus | oreganus | USA | California | Madera |
| CAS | 192630 | Crotalus | oreganus | oreganus | USA | California | Madera |
| CAS | 192633 | Crotalus | oreganus | oreganus | USA | California | Santa Clara |
| CAS | 192635 | Crotalus | oreganus | oreganus | USA | California | Santa Clara |
| CAS | 192637 | Crotalus | oreganus | lutosus | USA | California | Modoc |
| CAS | 192638 | Crotalus | oreganus | helleri | USA | California | San Diego |
| CAS | 192640 | Crotalus | oreganus | helleri | USA | California | San Diego |
| CAS | 192646 | Crotalus | oreganus | oreganus | USA | California | Kern |
| CAS | 192647 | Crotalus | oreganus | lutosus | USA | Nevada | Washoe |
| CAS | 192648 | Crotalus | oreganus | lutosus | USA | Nevada | Washoe |
| CAS | 192661 | Crotalus | oreganus | oreganus | USA | California | Santa Clara |
| CAS | 192663 | Crotalus | oreganus | oreganus | USA | California | Santa Clara |
| CAS | 192665 | Crotalus | oreganus | oreganus | USA | California | Santa Clara |
| CAS | 192669 | Crotalus | oreganus | oreganus | USA | California | Santa Clara |
| CAS | 192670 | Crotalus | oreganus | oreganus | USA | California | Santa Clara |
| CAS | 192672 | Crotalus | oreganus | oreganus | USA | California | Santa Clara |
| CAS | 192674 | Crotalus | oreganus | oreganus | USA | California | Santa Clara |
| CAS | 192675 | Crotalus | oreganus | oreganus | USA | California | Kern |
| CAS | 192676 | Crotalus | oreganus | oreganus | USA | California | El Dorado |
| CAS | 192683 | Crotalus | oreganus | oreganus | USA | California | San Luis Obispo |
| CAS | 192684 | Crotalus | oreganus | caliginis | Mexico | Baja California Norte |  |
| CAS | 192686 | Crotalus | oreganus | caliginis | Mexico | Baja California Norte |  |
| CAS | 192704 | Crotalus | oreganus | oreganus | USA | California | Tehema |
| CAS | 192707 | Crotalus | oreganus | oreganus | USA | California | Alameda |
| CAS | 192795 | Crotalus | viridis | viridis | USA | Arizona | Apache |
| CAS | 192796 | Crotalus | viridis | viridis | USA | Arizona | Apache |
| CAS | 192798 | Crotalus | viridis | viridis | USA | Arizona | Navajo |
| CAS | 192799 | Crotalus | viridis | viridis | USA | Arizona | Apache |
| CAS | 192800 | Crotalus | viridis | viridis | USA | Arizona | Apache |
| CAS | 192801 | Crotalus | viridis | viridis | USA | Arizona | Apache |
| CAS | 192802 | Crotalus | viridis | nuntius | USA | Arizona | Gila |
| CAS | 192803 | Crotalus | viridis | viridis | USA | Arizona | Apache |
| CAS | 192804 | Crotalus | viridis | viridis | USA | Arizona | Apache |
| CAS | 192805 | Crotalus | viridis | viridis | USA | Arizona | Apache |
| CAS | 192807 | Crotalus | viridis | viridis | USA | Arizona | Apache |
| CAS | 192808 | Crotalus | viridis | viridis | USA | Arizona | Apache |
| CAS | 192809 | Crotalus | viridis | nuntius | USA | Arizona | Coconino |
| CAS | 195041 | Crotalus | oreganus | lutosus | USA | Nevada | Nye |
| CAS | 196056 | Crotalus | oreganus | helleri | USA | California | Los Angeles |
| CAS | 196342 | Crotalus | oreganus | oreganus | USA | California | Mendocino |
| CAS | 197570 | Crotalus | oreganus | helleri | USA | California | San Luis Obispo |
| CAS | 197587 | Crotalus | oreganus | helleri | USA | California | Los Angeles |
| CAS | 198624 | Crotalus | oreganus | oreganus | USA | California | San Joaquin |
| CAS | 200712 | Crotalus | oreganus | helleri | USA | California | Riverside |
| CAS | 200713 | Crotalus | oreganus | helleri | USA | California | Riverside |
| CAS | 201282 | Crotalus | oreganus | lutosus | USA | Nevada | Washoe |
| CAS | 201506 | Crotalus | oreganus | helleri | USA | California | San Diego |
| CAS | 202876 | Crotalus | oreganus | oreganus | USA | California | Sierra |
| CAS | 202955 | Crotalus | oreganus | lutosus | USA | Nevada | Washoe |
| CAS | 202964 | Crotalus | oreganus | lutosus | USA | Nevada | Washoe |
| CAS | 202965 | Crotalus | oreganus | lutosus | USA | Nevada | Washoe |
| CAS | 202966 | Crotalus | oreganus | lutosus | USA | Nevada | Washoe |
| CAS | 202967 | Crotalus | oreganus | lutosus | USA | Nevada | Washoe |
| CAS | 202974 | Crotalus | oreganus | lutosus | USA | Nevada | Washoe |
| CAS | 202979 | Crotalus | oreganus | lutosus | USA | Nevada | Washoe |
| CAS | 203605 | Crotalus | oreganus | oreganus | USA | California | Lake |
| CAS | 203648 | Crotalus | oreganus | oreganus | USA | California | Lake |
| CAS | 204039 | Crotalus | viridis | viridis | USA | Utah | San Juan |
| CAS | 204070 | Crotalus | viridis | viridis | USA | Utah | San Juan |
| CAS | 205205 | Crotalus | oreganus | oreganus | USA | California | Butte |
| CAS | 205756 | Crotalus | oreganus | oreganus | USA | California | Alameda |
| CAS | 205793 | Crotalus | oreganus | oreganus | USA | California | Monterey |
| CAS | 205951 | Crotalus | oreganus | oreganus | USA | California | Sierra |
| CAS | 205965 | Crotalus | oreganus | oreganus | USA | California | Plumas |
| CAS | 206022 | Crotalus | oreganus | oreganus | USA | California | Plumas |
| CAS | 206136 | Crotalus | oreganus | oreganus | USA | California | Plumas |
| CAS | 206278 | Crotalus | oreganus | oreganus | USA | California | Plumas |
| CAS | 206279 | Crotalus | oreganus | oreganus | USA | California | Plumas |
| CAS | 206280 | Crotalus | oreganus | oreganus | USA | California | Plumas |
| CAS | 207163 | Crotalus | oreganus | oreganus | USA | California | Colusa |
| CAS | 207165 | Crotalus | oreganus | oreganus | USA | California | Colusa |
| CAS | 208764 | Crotalus | oreganus | oreganus | USA | California | Fresno |
| CAS | 208784 | Crotalus | oreganus | oreganus | USA | California | Fresno |
| CAS | 208785 | Crotalus | oreganus | oreganus | USA | California | Fresno |
| CAS | 208817 | Crotalus | oreganus | oreganus | USA | California | Fresno |
| CAS | 208915 | Crotalus | oreganus | oreganus | USA | California | Mariposa |
| CAS | 209076 | Crotalus | oreganus | oreganus | USA | California | Mendocino |
| CAS | 209200 | Crotalus | oreganus | oreganus | USA | California | Mariposa |
| CAS | 209238 | Crotalus | oreganus | oreganus | USA | California | Madera |
| CAS | 209459 | Crotalus | oreganus | oreganus | USA | California | Plumas |
| CAS | 209705 | Crotalus | oreganus | oreganus | USA | California | Yuba |
| CAS | 210387 | Crotalus | oreganus | oreganus | USA | California | Monterey |
| CAS | 210388 | Crotalus | oreganus | oreganus | USA | California | Monterey |
| CAS | 210427 | Crotalus | oreganus | oreganus | USA | California | Santa Cruz |
| CAS | 210462 | Crotalus | oreganus | oreganus | USA | California | Colusa |
| CAS | 210464 | Crotalus | oreganus | oreganus | USA | California | Alameda |
| CAS | 212273 | Crotalus | oreganus | oreganus | USA | California | Fresno |
| CAS | 212754 | Crotalus | oreganus | oreganus | USA | California | Colusa |
| CAS | 212761 | Crotalus | oreganus | oreganus | USA | California | Lake |
| CAS | 212781 | Crotalus | oreganus | oreganus | USA | California | Tulare |
| CAS | 212819 | Crotalus | oreganus | oreganus | USA | California | Tulare |
| CAS | 212859 | Crotalus | oreganus | oreganus | USA | California | Tulare |
| CAS | 212897 | Crotalus | oreganus | oreganus | USA | California | Tulare |
| CAS | 212985 | Crotalus | oreganus | oreganus | USA | California | Madera |
| CAS | 213058 | Crotalus | oreganus | oreganus | USA | California | Fresno |
| CAS | 213160 | Crotalus | oreganus | oreganus | USA | California | Tulare |
| CAS | 214824 | Crotalus | oreganus | oreganus | USA | California | Contra Costa |
| CAS | 219444 | Crotalus | oreganus | oreganus | USA | California | Lake |
| CAS | 219472 | Crotalus | oreganus | oreganus | USA | California | Lake |
| CAS | 219609 | Crotalus | oreganus | oreganus | USA | California | Tulare |
| CAS | 220853 | Crotalus | oreganus | oreganus | USA | California | Tulare |
| CAS | 220949 | Crotalus | oreganus | oreganus | USA | California | Lake |
| CAS | 223383 | Crotalus | oreganus | lutosus | USA | Nevada | White Pine |
| CAS | 223384 | Crotalus | oreganus | lutosus | USA | Nevada | White Pine |
| CAS | 223404 | Crotalus | oreganus | lutosus | USA | Nevada | White Pine |
| CAS | 223410 | Crotalus | oreganus | lutosus | USA | Nevada | White Pine |
| CAS | 223558 | Crotalus | oreganus | lutosus | USA | Nevada | Lander |
| CAS | 223564 | Crotalus | viridis | viridis | USA | Colorado | Las Animas |
| CAS | 223599 | Crotalus | oreganus | helleri | USA | California | San Diego |
| CAS | 223729 | Crotalus | oreganus | lutosus | USA | California | Kern |
| CAS | 223730 | Crotalus | oreganus | helleri | USA | California | San Luis Obispo |
| CAS | 223730 | Crotalus | oreganus | helleri | USA | California | San Luis Obispo |
| CAS | 223732 | Crotalus | oreganus | oreganus | USA | California | Kern |
| CAS | 223735 | Crotalus | oreganus | oreganus | USA | California | Kern |
| CAS | 224198 | Crotalus | oreganus | oreganus | USA | California | Kern |
| CAS | 224199 | Crotalus | oreganus | oreganus | USA | California | Tulare |
| CAS | 224764 | Crotalus | oreganus | oreganus | USA | California | Kern |
| CAS | 224859 | Crotalus | oreganus | oreganus | USA | California | Fresno |
| CAS | 224926 | Crotalus | oreganus | oreganus | USA | California | Tulare |
| CAS | 225292 | Crotalus | oreganus | oreganus | USA | California | San Joaquin |
| CAS | 226092 | Crotalus | oreganus | helleri | USA | California | Los Angeles |
| CAS | 227908 | Crotalus | oreganus | oreganus | USA | California | Modoc |
| CAS | 227921 | Crotalus | oreganus | lutosus | USA | Nevada | Washoe |
| CAS | 227957 | Crotalus | oreganus | lutosus | USA | Nevada | White Pine |
| CAS | 228044 | Crotalus | oreganus | helleri | USA | California | San Diego |
| CAS | 228048 | Crotalus | oreganus | helleri | USA | California | San Diego |
| CAS | 228052 | Crotalus | oreganus | helleri | USA | California | San Diego |
| CAS | 228193 | Crotalus | oreganus | oreganus | USA | California | Santa Clara |
| CAS | 228214 | Crotalus | oreganus | oreganus | USA | California | Kern |
| CAS | 229227 | Crotalus | oreganus | unknown | USA | Utah | Garfield |
| CAS | 229236 | Crotalus | viridis | viridis | USA | New Mexico | Grant |
| CAS | 229242 | Crotalus | oreganus | concolor | USA | Utah | Carbon |
| CAS | 229243 | Crotalus | viridis | nuntius | USA | Arizona | Navajo |
| CAS | 232565 | Crotalus | unknown | unknown |  |  |  |
| CAS | 234600 | Crotalus | oreganus | unknown | USA | California | Plumas |
| CAS | 234623 | Crotalus | oreganus | oreganus | USA | California | Butte |
| CAS | 234673 | Crotalus | oreganus | oreganus | USA | California | Plumas |
| CAS | 234674 | Crotalus | oreganus | oreganus | USA | California | Plumas |
| CAS | 235854 | Crotalus | oreganus | oreganus | USA | California | Alameda |
| CAS | 235855 | Crotalus | oreganus | oreganus | USA | California | Alameda |
| CAS | 236038 | Crotalus | oreganus | oreganus | USA | California | Alameda |
| CAS | 236216 | Crotalus | oreganus | oreganus | USA | California | Kern |
| CAS | 238534 | Crotalus | oreganus | oreganus | USA | California | San Joaquin |
| CAS | 238943 | Crotalus | oreganus | oreganus | USA | California | Contra Costa |
| CAS | 241772 | Crotalus | oreganus | oreganus | USA | California | Alameda |
| CM | 4500 | Crotalus | oreganus | lutosus | USA | Idaho | Elmore |
| CM | 4500 | Crotalus | oreganus | lutosus | USA | Idaho | Elmore |
| CM | 4748 | Crotalus | oreganus | lutosus | USA | Idaho | Boise |
| CM | 4748 | Crotalus | oreganus | lutosus | USA | Idaho | Boise |
| CM | 6162 | Crotalus | oreganus | concolor | USA | Utah | Uintah |
| CM | 6162 | Crotalus | oreganus | concolor | USA | Utah | Uintah |
| CM | 12342 | Crotalus | oreganus | concolor | USA | Utah | Uintah |
| CM | 12342 | Crotalus | oreganus | concolor | USA | Utah | Uintah |
| CM | 12355 | Crotalus | oreganus | concolor | USA | Colorado | Moffat |
| CM | 12355 | Crotalus | oreganus | concolor | USA | Colorado | Moffat |
| CM | 12368 | Crotalus | oreganus | concolor | USA | Utah | Uintah |
| CM | 12368 | Crotalus | oreganus | concolor | USA | Utah | Uintah |
| CM | 12426 | Crotalus | oreganus | concolor | USA | Utah | Uintah |
| CM | 12426 | Crotalus | oreganus | concolor | USA | Utah | Uintah |
| CM | 12427 | Crotalus | oreganus | concolor | USA | Utah | Uintah |
| CM | 12427 | Crotalus | oreganus | concolor | USA | Utah | Uintah |
| CM | 12451 | Crotalus | oreganus | concolor | USA | Utah | Uintah |
| CM | 12451 | Crotalus | oreganus | concolor | USA | Utah | Uintah |
| CM | 12491 | Crotalus | oreganus | lutosus | USA | Utah | Washington |
| CM | 12491 | Crotalus | oreganus | lutosus | USA | Utah | Washington |
| CM | 12496 | Crotalus | oreganus | lutosus | USA | Utah | Washington |
| CM | 12496 | Crotalus | oreganus | lutosus | USA | Utah | Washington |
| CM | 16749 | Crotalus | oreganus | lutosus | USA | Utah | Box Elder |
| CM | 16749 | Crotalus | oreganus | lutosus | USA | Utah | Box Elder |
| CM | 18306 | Crotalus | oreganus | cerberus | USA | New Mexico | Eddy |
| CM | 18306 | Crotalus | oreganus | cerberus | USA | New Mexico | Eddy |
| CM | 18476 | Crotalus | oreganus | cerberus | USA | Arizona | Coconino |
| CM | 18745 | Crotalus | oreganus | cerberus | USA | Arizona | Coconino |
| CM | 18745 | Crotalus | oreganus | cerberus | USA | Arizona | Coconino |
| CM | 18746 | Crotalus | oreganus | cerberus | USA | Arizona | Coconino |
| CM | 20294 | Crotalus | oreganus | abyssus | USA | Arizona | Coconino |
| CM | 20294 | Crotalus | oreganus | abyssus | USA | Arizona | Coconino |
| CM | 26674 | Crotalus | oreganus | cerberus | USA | Arizona |  |
| CM | 26674 | Crotalus | oreganus | cerberus | USA | Arizona |  |
| CM | 26708 | Crotalus | oreganus | cerberus | USA | Arizona |  |
| CM | 26708 | Crotalus | oreganus | cerberus | USA | Arizona |  |
| CM | 42850 | Crotalus | oreganus | concolor | USA | Colorado | Mesa |
| CM | 42850 | Crotalus | oreganus | concolor | USA | Colorado | Mesa |
| CM | 42852 | Crotalus | oreganus | concolor | USA | Colorado | Mesa |
| CM | 42852 | Crotalus | oreganus | concolor | USA | Colorado | Mesa |
| CM | 42853 | Crotalus | oreganus | concolor | USA | Colorado | Mesa |
| CM | 42853 | Crotalus | oreganus | concolor | USA | Colorado | Mesa |
| CM | 42854 | Crotalus | oreganus | concolor | USA | Colorado | Mesa |
| CM | 42854 | Crotalus | oreganus | concolor | USA | Colorado | Mesa |
| CM | 47741 | Crotalus | oreganus | cerberus | USA | Arizona | Maricopa |
| CM | 47741 | Crotalus | oreganus | cerberus | USA | Arizona | Maricopa |
| CM | 48740 | Crotalus | oreganus | cerberus | USA | Arizona | Graham |
| CM | 48740 | Crotalus | oreganus | cerberus | USA | Arizona | Graham |
| CM | 51382 | Crotalus | oreganus | cerberus | USA | Arizona | Gila |
| CM | 51382 | Crotalus | oreganus | cerberus | USA | Arizona | Gila |
| CM | 51521 | Crotalus | oreganus | cerberus | USA | Arizona | Gila |
| CM | 51521 | Crotalus | oreganus | cerberus | USA | Arizona | Gila |
| CM | 51833 | Crotalus | oreganus | cerberus | USA | Arizona | Gila |
| CM | 51833 | Crotalus | oreganus | cerberus | USA | Arizona | Gila |
| CM | 51834 | Crotalus | oreganus | cerberus | USA | Arizona | Gila |
| CM | 51834 | Crotalus | oreganus | cerberus | USA | Arizona | Gila |
| CM | 51835 | Crotalus | oreganus | cerberus | USA | Arizona | Gila |
| CM | 51835 | Crotalus | oreganus | cerberus | USA | Arizona | Gila |
| CM | 51914 | Crotalus | oreganus | cerberus | USA | Arizona | Gila |
| CM | 51914 | Crotalus | oreganus | cerberus | USA | Arizona | Gila |
| CM | 51915 | Crotalus | oreganus | cerberus | USA | Arizona | Gila |
| CM | 51915 | Crotalus | oreganus | cerberus | USA | Arizona | Gila |
| CM | 67068 | Crotalus | oreganus | cerberus | USA | Arizona | Yavapai |
| CM | 67068 | Crotalus | oreganus | cerberus | USA | Arizona | Yavapai |
| CM | 67068 | Crotalus | oreganus | cerberus | USA | Arizona | Yavapai |
| CM | 67068 | Crotalus | oreganus | cerberus | USA | Arizona | Yavapai |
| CM | 70922 | Crotalus | oreganus | cerberus | USA | Arizona | Graham |
| CM | 70922 | Crotalus | oreganus | cerberus | USA | Arizona | Graham |
| CM | 70929 | Crotalus | oreganus | cerberus | USA | Arizona | Graham |
| CM | 70929 | Crotalus | oreganus | cerberus | USA | Arizona | Graham |
| CM | 70951 | Crotalus | oreganus | cerberus | USA | Arizona | Graham |
| CM | 70951 | Crotalus | oreganus | cerberus | USA | Arizona | Graham |
| CM | 70975 | Crotalus | oreganus | cerberus | USA | Arizona | Graham |
| CM | 70975 | Crotalus | oreganus | cerberus | USA | Arizona | Graham |
| CM | 71095 | Crotalus | oreganus | cerberus | USA | Arizona | Greenlee |
| CM | 71095 | Crotalus | oreganus | cerberus | USA | Arizona | Greenlee |
| INHS | 173 | Crotalus | oreganus | helleri | USA | California | Riverside |
| INHS | 1721 | Crotalus | oreganus | helleri | USA | California | Los Angeles |
| INHS | 2214 | Crotalus | oreganus | lutosus | USA | Utah | Millard |
| INHS | 2215 | Crotalus | oreganus | lutosus | USA | Utah | Millard |
| INHS | 2216 | Crotalus | oreganus | lutosus | USA | Utah | Millard |
| INHS | 2636 | Crotalus | viridis | nuntius | USA | Arizona | Coconino |
| INHS | 5127 | Crotalus | oreganus | helleri | USA | California | Ventura |
| INHS | 6000 | Crotalus | oreganus | oreganus | USA | California | Santa Cruz |
| INHS | 6001 | Crotalus | oreganus | oreganus | USA | California | Monterey |
| INHS | 6002 | Crotalus | oreganus | helleri | USA | California | San Diego |
| INHS | 6003 | Crotalus | oreganus | lutosus | USA | Nevada | Washoe |
| INHS | 6004 | Crotalus | viridis | viridis | USA | New Mexico | Dona Ana |
| INHS | 6006 | Crotalus | viridis | viridis | USA | New Mexico | Torrance |
| INHS | 6385 | Crotalus | viridis | viridis | USA | Texas | El Paso |
| INHS | 17480 | Crotalus | oreganus | oreganus | USA | California | Santa Barbara |
| INHS | 24542 | Crotalus | oreganus | cerberus | USA | Arizona | Graham |
| INHS | 24543 | Crotalus | oreganus | cerberus | USA | Arizona | Graham |
| INHS | 27756 | Crotalus | oreganus | lutosus | USA | Utah | Washington |
| INHS | 31351 | Crotalus | oreganus | cerberus | USA | Arizona | Graham |
| INHS | 39639 | Crotalus | viridis | viridis | USA | Colorado | Lincoln |
| INHS | 39639 | Crotalus | viridis | viridis | USA | Colorado | Lincoln |
| INHS | 39641 | Crotalus | viridis | viridis | USA | New Mexico | Chaves |
| INHS | 50258 | Crotalus | viridis | viridis | USA | Wyoming | Niobrara |
| INHS | 50286 | Crotalus | viridis | viridis | USA | Colorado | Weld |
| INHS | 50287 | Crotalus | viridis | viridis | USA | Colorado | Weld |
| INHS | 50288 | Crotalus | viridis | viridis | USA | Colorado | Elbert |
| INHS | 50289 | Crotalus | viridis | viridis | USA | Colorado | Elbert |
| INHS | 50290 | Crotalus | viridis | viridis | USA | Colorado | Baca |
| INHS | 50291 | Crotalus | viridis | viridis | USA | Colorado | Prowers |
| INHS | 50292 | Crotalus | viridis | viridis | USA | Colorado | Lincoln |
| INHS | 51291 | Crotalus | oreganus | lutosus | USA | Utah | Cache |
| INHS | 52079 | Crotalus | viridis | viridis | USA | Wyoming | Platte |
| INHS | 52100 | Crotalus | oreganus | oreganus | USA | Washington | Franklin |
| INHS | 52101 | Crotalus | viridis | viridis | USA | Montana | Rosebud |
| INHS | 52102 | Crotalus | viridis | nuntius | USA | Arizona | Cocorro |
| INHS | 53165 | Crotalus | viridis | viridis | USA | Colorado | Boulder |
| INHS | 53166 | Crotalus | viridis | viridis | USA | Colorado | Weld |
| INHS | 53167 | Crotalus | viridis | viridis | USA | Colorado | Weld |
| INHS | 53169 | Crotalus | viridis | viridis | USA | Colorado | Weld |
| INHS | 53171 | Crotalus | viridis | viridis | USA | Colorado | Jefferson |
| INHS | 53172 | Crotalus | viridis | viridis | USA | Colorado | Cheyenne |
| INHS | 53173 | Crotalus | viridis | viridis | USA | Colorado | Cheyenne |
| INHS | 53174 | Crotalus | viridis | viridis | USA | Colorado | Fremont |
| INHS | 53175 | Crotalus | viridis | viridis | USA | Wyoming | Campbell |
| INHS | 53176 | Crotalus | viridis | viridis | USA | Colorado | Baca |
| INHS | 53177 | Crotalus | viridis | viridis | USA | Wyoming | Campbell |
| INHS | 53178 | Crotalus | viridis | viridis | USA | Wyoming | Albany |
| INHS | 56736 | Crotalus | viridis | viridis | USA | Colorado | Baca |
| INHS | 61420 | Crotalus | viridis | nuntius | USA | Arizona | Coconino |
| INHS | 61421 | Crotalus | viridis | nuntius | USA | Arizona | Navajo |
| INHS | 62440 | Crotalus | viridis | viridis | USA | South Dakota | Pennington |
| INHS | 62440 | Crotalus | viridis | viridis | USA | South Dakota | Pennington |
| INHS | 64677 | Crotalus | viridis | viridis | USA | Montana | Big Horn |
| INHS | 64678 | Crotalus | viridis | viridis | USA | Montana | Big Horn |
| INHS | 65680 | Crotalus | viridis | viridis | USA | Colorado | Grande |
| INHS | 66467 | Crotalus | viridis | viridis | USA | Colorado | Cheyenne |
| INHS | 78029 | Crotalus | viridis | viridis | USA | North Dakota |  |
| INHS | 78791 | Crotalus | viridis | viridis | USA | Wyoming | Albany |
| INHS | 80488 | Crotalus | viridis | viridis | USA | Colorado | Cheyenne |
| INHS | 80489 | Crotalus | viridis | viridis | USA | Colorado | Cheyenne |
| INHS | 80490 | Crotalus | viridis | viridis | USA | Colorado | Cheyenne |
| INHS | 80491 | Crotalus | viridis | viridis | USA | Colorado | Cheyenne |
| INHS | 80492 | Crotalus | viridis | viridis | USA | Colorado | Cheyenne |
| INHS | 80493 | Crotalus | viridis | viridis | USA | Colorado | Cheyenne |
| INHS | 84854 | Crotalus | oreganus | cerberus | USA | Arizona | Gila |
| INHS | 84855 | Crotalus | oreganus | cerberus | USA | Arizona | Yavapai |
| INHS | 84856 | Crotalus | oreganus | oreganus | USA | California | Butte |
| INHS | 84857 | Crotalus | oreganus | helleri | USA | California | San Diego |
| INHS | 84858 | Crotalus | oreganus | helleri | USA | California | San Diego |
| INHS | 84859 | Crotalus | oreganus | helleri | USA | California | San Diego |
| INHS | 84860 | Crotalus | oreganus | helleri | USA | California | San Diego |
| INHS | 84861 | Crotalus | viridis | nuntius | USA | Arizona | Coconino |
| INHS | 84862 | Crotalus | viridis | viridis | USA | Montana | Jefferson |
| INHS | 85861 | Crotalus | oreganus | oreganus | USA | Washington | Shannon |
| INHS | 91019 | Crotalus | viridis | viridis | USA | New Mexico | Dona Ana |
| INHS | 93456 | Crotalus | oreganus | oreganus | USA | Oregon | Lane |
| INHS | 93541 | Crotalus | oreganus | cerberus | USA | Arizona | Pima |
| MCZ | 43392 | Crotalus | oreganus | concolor | USA | Utah | Jensen |
| MCZ | 43392 | Crotalus | oreganus | concolor | USA | Utah | Jensen |
| MCZ | 43394 | Crotalus | oreganus | concolor | USA | Utah | Jensen |
| MCZ | 43394 | Crotalus | oreganus | concolor | USA | Utah | Jensen |
| MCZ | 43395 | Crotalus | oreganus | concolor | USA | Utah | Jensen |
| MCZ | 43395 | Crotalus | oreganus | concolor | USA | Utah | Jensen |
| MCZ | 44351 | Crotalus | oreganus | concolor | USA | Utah | Jensen |
| MCZ | 44351 | Crotalus | oreganus | concolor | USA | Utah | Jensen |
| MCZ | 44352 | Crotalus | oreganus | concolor | USA | Utah | Jensen |
| MCZ | 44352 | Crotalus | oreganus | concolor | USA | Utah | Jensen |
| MCZ | 44354 | Crotalus | oreganus | concolor | USA | Utah | Jensen |
| MCZ | 44354 | Crotalus | oreganus | concolor | USA | Utah | Jensen |
| MCZ | 44355 | Crotalus | oreganus | concolor | USA | Utah | Jensen |
| MCZ | 44355 | Crotalus | oreganus | concolor | USA | Utah | Jensen |
| MCZ | 44815 | Crotalus | oreganus | lutosus | USA | Idaho | Ada |
| MCZ | 44815 | Crotalus | oreganus | lutosus | USA | Idaho |  |
| MCZ | 44816 | Crotalus | oreganus | lutosus | USA | Idaho | Ada |
| MCZ | 44816 | Crotalus | oreganus | lutosus | USA | Idaho |  |
| MCZ | 44817 | Crotalus | oreganus | lutosus | USA | Idaho | Ada |
| MCZ | 44817 | Crotalus | oreganus | lutosus | USA | Idaho |  |
| MCZ | 44818 | Crotalus | oreganus | lutosus | USA | Idaho | Ada |
| MCZ | 44818 | Crotalus | oreganus | lutosus | USA | Idaho |  |
| MCZ | 45225 | Crotalus | oreganus | abyssus | USA | Arizona | Coconino |
| MCZ | 45225 | Crotalus | oreganus | abyssus | USA | Arizona |  |
| MCZ | 65450 | Crotalus | oreganus | cerberus | USA | Arizona | Pima |
| MCZ | 65450 | Crotalus | oreganus | cerberus | USA | Arizona |  |
| MCZ | 101216 | Crotalus | viridis | nuntius | USA | Arizona | Coconino |
| MCZ | 101216 | Crotalus | viridis | nuntius | USA | Arizona | Coconino |
| MCZ | 132750 | Crotalus | oreganus | cerberus | USA | Arizona | Navajo |
| MCZ | 132750 | Crotalus | oreganus | cerberus | USA | Arizona | Navajo |
| MNA | Z7.13 | Crotalus | viridis | nuntius | USA | Arizona | Coconino |
| MNA | Z7.14 | Crotalus | viridis | nuntius | USA | Arizona | Coconino |
| MNA | Z7.141 | Crotalus | oreganus | abyssus | USA | Arizona | Coconino |
| MNA | Z7.142 | Crotalus | oreganus | cerberus | USA | Arizona | Coconino |
| MNA | Z7.143 | Crotalus | viridis | nuntius | USA | Arizona |  |
| MNA | Z7.148 | Crotalus | viridis | nuntius | USA | Arizona | Coconino |
| MNA | Z7.186 | Crotalus | viridis | viridis | USA | Utah |  |
| MNA | Z7.186 | Crotalus | viridis | viridis | USA | Utah |  |
| MNA | Z7.209 | Crotalus | viridis | nuntius | USA | Arizona | Navajo |
| MNA | Z7.212 | Crotalus | oreganus | cerberus | USA | Arizona | Yavapai |
| MNA | Z7.2224 | Crotalus | viridis | nuntius | USA | Arizona | Coconino |
| MNA | Z7.231 | Crotalus | viridis | nuntius | USA | Arizona | Coconino |
| MNA | Z7.2318 | Crotalus | oreganus | abyssus | USA | Arizona | Coconino |
| MNA | Z7.234 | Crotalus | viridis | nuntius | USA | Arizona |  |
| MNA | Z7.2361 | Crotalus | oreganus | abyssus | USA | Arizona | Coconino |
| MNA | Z7.2389 | Crotalus | oreganus | cerberus | USA | Arizona | Yavapai |
| MNA | Z7.241 | Crotalus | viridis | nuntius | USA | Utah | Kane |
| MNA | Z7.2481 | Crotalus | oreganus | abyssus | USA | Arizona | Coconino |
| MNA | Z7.255 | Crotalus | oreganus | lutosus | USA | Arizona | Coconino |
| MNA | Z7.2566 | Crotalus | oreganus | abyssus | USA | Arizona | Coconino |
| MNA | Z7.257 | Crotalus | oreganus | cerberus | USA | Arizona | Yavapai |
| MNA | Z7.2628 | Crotalus | oreganus | abyssus | USA | Arizona | Coconino |
| MNA | Z7.2798 | Crotalus | oreganus | lutosus | USA | Arizona | Mojave |
| MNA | Z7.2832 | Crotalus | oreganus | abyssus | USA | Arizona | Coconino |
| MNA | Z7.3 | Crotalus | viridis | nuntius | USA | Arizona | Coconino |
| MNA | Z7.43 | Crotalus | viridis | nuntius | USA | Arizona | Coconino |
| MNA | Z7.44 | Crotalus | viridis | nuntius | USA | Arizona | Coconino |
| MNA | Z7.472 | Crotalus | viridis | nuntius | USA | Arizona | Coconino |
| MNA | Z7.474 | Crotalus | viridis | nuntius | USA | Arizona | Coconino |
| MNA | Z7.617 | Crotalus | oreganus | abyssus | USA | Arizona | Coconino |
| MNA | Z7.740 | Crotalus | oreganus | abyssus | USA | Arizona | Coconino |
| MNA | Z7.76 | Crotalus | viridis | nuntius | USA | Arizona | Coconino |
| MNA | Z7.76 | Crotalus | viridis | nuntius | USA | Arizona | Coconino |
| MNA | Z7.79 | Crotalus | oreganus | cerberus | USA | Arizona | Coconino |
| MNA | Z7.80 | Crotalus | oreganus | cerberus | USA | Arizona | Coconino |
| MNA | Z7.88 | Crotalus | viridis | nuntius | USA | Arizona | Coconino |
| MNA | Z7.92 | Crotalus | viridis | nuntius | USA | Arizona | Coconino |
| SDSNH | 192 | Crotalus | viridis | nuntius | USA | Arizona | Navajo |
| SDSNH | 196 | Crotalus | viridis | nuntius | USA | Arizona | Navajo |
| SDSNH | 197 | Crotalus | viridis | nuntius | USA | Arizona | Navajo |
| SDSNH | 210 | Crotalus | oreganus | helleri | USA | California | San Diego |
| SDSNH | 288 | Crotalus | viridis | viridis | USA | Kansas | Trego |
| SDSNH | 289 | Crotalus | viridis | viridis | USA | Kansas | Trego |
| SDSNH | 537 | Crotalus | oreganus | helleri | USA | California | San Diego |
| SDSNH | 653 | Crotalus | oreganus | lutosus | USA | Utah | Millard |
| SDSNH | 656 | Crotalus | oreganus | helleri | USA | California | San Diego |
| SDSNH | 740 | Crotalus | oreganus | lutosus | USA | Idaho | Ada |
| SDSNH | 741 | Crotalus | oreganus | helleri | USA | California | Riverside |
| SDSNH | 768 | Crotalus | viridis | nuntius | USA | Arizona | Coconino |
| SDSNH | 769 | Crotalus | viridis | nuntius | USA | Arizona | Coconino |
| SDSNH | 772 | Crotalus | oreganus | oreganus | USA | Washington | Klickitat |
| SDSNH | 775 | Crotalus | viridis | nuntius | USA | Arizona | Navajo |
| SDSNH | 776 | Crotalus | viridis | nuntius | USA | Arizona | Navajo |
| SDSNH | 777 | Crotalus | viridis | nuntius | USA | Arizona | Navajo |
| SDSNH | 778 | Crotalus | viridis | nuntius | USA | Arizona | Navajo |
| SDSNH | 779 | Crotalus | viridis | nuntius | USA | Arizona | Navajo |
| SDSNH | 780 | Crotalus | viridis | nuntius | USA | Arizona | Navajo |
| SDSNH | 781 | Crotalus | viridis | nuntius | USA | Arizona | Navajo |
| SDSNH | 789 | Crotalus | viridis | nuntius | USA | Arizona | Navajo |
| SDSNH | 834 | Crotalus | oreganus | helleri | USA | California | San Diego |
| SDSNH | 835 | Crotalus | oreganus | oreganus | USA | Idaho | Elmore |
| SDSNH | 862 | Crotalus | oreganus | oreganus | USA | Idaho | Owyhee |
| SDSNH | 863 | Crotalus | oreganus | oreganus | USA | Idaho | Elmore |
| SDSNH | 868 | Crotalus | viridis | nuntius | USA | Arizona | Navajo |
| SDSNH | 967 | Crotalus | oreganus | helleri | USA | California | San Diego |
| SDSNH | 1118 | Crotalus | viridis | nuntius | USA | Arizona | Navajo |
| SDSNH | 1144 | Crotalus | viridis | nuntius | USA | Arizona | Coconino |
| SDSNH | 1171 | Crotalus | viridis | nuntius | USA | Arizona | Coconino |
| SDSNH | 1179 | Crotalus | viridis | nuntius | USA | Arizona | Navajo |
| SDSNH | 1180 | Crotalus | viridis | nuntius | USA | Arizona | Coconino |
| SDSNH | 1193 | Crotalus | viridis | nuntius | USA | Arizona | Coconino |
| SDSNH | 1195 | Crotalus | viridis | nuntius | USA | Arizona | Coconino |
| SDSNH | 1196 | Crotalus | viridis | nuntius | USA | Arizona | Coconino |
| SDSNH | 1229 | Crotalus | oreganus | helleri | USA | California | San Diego |
| SDSNH | 1285 | Crotalus | oreganus | helleri | USA | California | San Diego |
| SDSNH | 1285 | Crotalus | oreganus | helleri | USA | California | San Diego |
| SDSNH | 1287 | Crotalus | oreganus | lutosus | USA | Utah | Millard |
| SDSNH | 1288 | Crotalus | oreganus | lutosus | USA | Utah | Millard |
| SDSNH | 1289 | Crotalus | oreganus | lutosus | USA | Utah | Millard |
| SDSNH | 1290 | Crotalus | viridis | nuntius | USA | Arizona | Navajo |
| SDSNH | 1304 | Crotalus | oreganus | lutosus | USA | Utah | Millard |
| SDSNH | 1305 | Crotalus | oreganus | lutosus | USA | Utah | Millard |
| SDSNH | 1425 | Crotalus | oreganus | lutosus | USA | Idaho | Ada |
| SDSNH | 1426 | Crotalus | oreganus | lutosus | USA | Idaho | Elmore |
| SDSNH | 1427 | Crotalus | oreganus | lutosus | USA | Idaho | Ada |
| SDSNH | 1428 | Crotalus | oreganus | lutosus | USA | Idaho | Elmore |
| SDSNH | 1429 | Crotalus | oreganus | lutosus | USA | Idaho | Ada |
| SDSNH | 1430 | Crotalus | oreganus | lutosus | USA | Idaho | Ada |
| SDSNH | 1495 | Crotalus | viridis | nuntius | USA | Arizona | Navajo |
| SDSNH | 1496 | Crotalus | viridis | nuntius | USA | Arizona | Navajo |
| SDSNH | 1550 | Crotalus | oreganus | lutosus | USA | Utah | Millard |
| SDSNH | 1551 | Crotalus | oreganus | lutosus | USA | Utah | Millard |
| SDSNH | 1552 | Crotalus | oreganus | helleri | USA | California | San Diego |
| SDSNH | 1576 | Crotalus | oreganus | helleri | USA | California | San Bernadino |
| SDSNH | 1617 | Crotalus | oreganus | lutosus | USA | Utah | Millard |
| SDSNH | 1618 | Crotalus | oreganus | lutosus | USA | Utah | Millard |
| SDSNH | 1647 | Crotalus | oreganus | cerberus | USA | Arizona | Pinal |
| SDSNH | 1677 | Crotalus | oreganus | helleri | USA | California | San Diego |
| SDSNH | 1725 | Crotalus | oreganus | helleri | USA | California | San Diego |
| SDSNH | 1725 | Crotalus | oreganus | helleri | USA | California | San Diego |
| SDSNH | 1733 | Crotalus | oreganus | helleri | USA | California | San Diego |
| SDSNH | 1738 | Crotalus | oreganus | helleri | USA | California | San Diego |
| SDSNH | 1800 | Crotalus | oreganus | lutosus | USA | Utah | Millard |
| SDSNH | 1801 | Crotalus | oreganus | lutosus | USA | Utah | Millard |
| SDSNH | 1802 | Crotalus | oreganus | lutosus | USA | Utah | Millard |
| SDSNH | 1805 | Crotalus | oreganus | lutosus | USA | Utah | Millard |
| SDSNH | 1806 | Crotalus | oreganus | lutosus | USA | Utah | Millard |
| SDSNH | 1807 | Crotalus | oreganus | lutosus | USA | Utah | Millard |
| SDSNH | 1808 | Crotalus | oreganus | lutosus | USA | Utah | Millard |
| SDSNH | 1810 | Crotalus | oreganus | lutosus | USA | Utah | Millard |
| SDSNH | 1811 | Crotalus | oreganus | lutosus | USA | Utah | Millard |
| SDSNH | 1812 | Crotalus | oreganus | lutosus | USA | Utah | Millard |
| SDSNH | 1813 | Crotalus | oreganus | lutosus | USA | Utah | Millard |
| SDSNH | 1814 | Crotalus | oreganus | lutosus | USA | Utah | Millard |
| SDSNH | 1815 | Crotalus | oreganus | lutosus | USA | Utah | Millard |
| SDSNH | 1816 | Crotalus | oreganus | lutosus | USA | Utah | Millard |
| SDSNH | 1853 | Crotalus | oreganus | lutosus | USA | Utah | Millard |
| SDSNH | 1854 | Crotalus | oreganus | lutosus | USA | Utah | Millard |
| SDSNH | 1855 | Crotalus | oreganus | lutosus | USA | Utah | Millard |
| SDSNH | 1867 | Crotalus | oreganus | lutosus | USA | Utah | Millard |
| SDSNH | 1868 | Crotalus | oreganus | lutosus | USA | Utah | Millard |
| SDSNH | 1869 | Crotalus | oreganus | lutosus | USA | Utah | Millard |
| SDSNH | 1880 | Crotalus | oreganus | oreganus | USA | Washington | Chelan |
| SDSNH | 1881 | Crotalus | oreganus | helleri | USA | California | Los Angeles |
| SDSNH | 1883 | Crotalus | oreganus | helleri | USA | California | Kern |
| SDSNH | 1887 | Crotalus | oreganus | oreganus | USA | Washington | Chelan |
| SDSNH | 1890 | Crotalus | oreganus | helleri | USA | California | San Diego |
| SDSNH | 1896 | Crotalus | oreganus | oreganus | USA | Washington | Chelan |
| SDSNH | 1897 | Crotalus | oreganus | oreganus | USA | Washington | Chelan |
| SDSNH | 1898 | Crotalus | oreganus | oreganus | USA | Washington | Chelan |
| SDSNH | 1899 | Crotalus | oreganus | oreganus | USA | Washington | Chelan |
| SDSNH | 1955 | Crotalus | oreganus | helleri | USA | California | San Diego |
| SDSNH | 1990 | Crotalus | oreganus | oreganus | USA | California | Santa Clara |
| SDSNH | 1991 | Crotalus | oreganus | oreganus | USA | California | Santa Clara |
| SDSNH | 1996 | Crotalus | oreganus | helleri | USA | California | San Diego |
| SDSNH | 2007 | Crotalus | oreganus | oreganus | USA | California | Mono |
| SDSNH | 2041 | Crotalus | viridis | nuntius | USA | Arizona | Navajo |
| SDSNH | 2042 | Crotalus | viridis | nuntius | USA | Arizona | Navajo |
| SDSNH | 2043 | Crotalus | viridis | nuntius | USA | Arizona | Navajo |
| SDSNH | 2052 | Crotalus | viridis | nuntius | USA | Arizona | Navajo |
| SDSNH | 2053 | Crotalus | viridis | nuntius | USA | Arizona | Navajo |
| SDSNH | 2054 | Crotalus | viridis | nuntius | USA | Arizona | Navajo |
| SDSNH | 2081 | Crotalus | oreganus | oreganus | USA | California | Mono |
| SDSNH | 2082 | Crotalus | oreganus | cerberus | USA | Arizona | Yavapai |
| SDSNH | 2083 | Crotalus | oreganus | oreganus | USA | California | Santa Clara |
| SDSNH | 2085 | Crotalus | viridis | nuntius | USA | Arizona | Navajo |
| SDSNH | 2086 | Crotalus | oreganus | helleri | USA | California | San Diego |
| SDSNH | 2093 | Crotalus | oreganus | abyssus | USA | Arizona | Coconino |
| SDSNH | 2135 | Crotalus | oreganus | cerberus | USA | Arizona | Yavapai |
| SDSNH | 2146 | Crotalus | viridis | viridis |  |  |  |
| SDSNH | 2154 | Crotalus | oreganus | cerberus | USA | Arizona | Yavapai |
| SDSNH | 2155 | Crotalus | oreganus | cerberus | USA | Arizona | Yavapai |
| SDSNH | 2180 | Crotalus | oreganus | helleri | USA | California | San Diego |
| SDSNH | 2211 | Crotalus | oreganus | helleri | USA | California | San Diego |
| SDSNH | 2214 | Crotalus | viridis | nuntius | USA | Arizona | Navajo |
| SDSNH | 2215 | Crotalus | oreganus | cerberus | USA | Arizona | Coconino |
| SDSNH | 2216 | Crotalus | oreganus | abyssus | USA | Arizona | Coconino |
| SDSNH | 2218 | Crotalus | oreganus | cerberus | USA | Arizona | Yavapai |
| SDSNH | 2262 | Crotalus | oreganus | oreganus | USA | California | Stanislaus |
| SDSNH | 2266 | Crotalus | viridis | viridis | USA | Kansas | Hodgeman |
| SDSNH | 2267 | Crotalus | viridis | nuntius | USA | Arizona | Coconino |
| SDSNH | 2268 | Crotalus | viridis | nuntius | USA | Arizona | Coconino |
| SDSNH | 2269 | Crotalus | viridis | nuntius | USA | Arizona | Coconino |
| SDSNH | 2272 | Crotalus | oreganus | abyssus | USA | Arizona | Coconino |
| SDSNH | 2280 | Crotalus | oreganus | lutosus | USA | Utah | Washington |
| SDSNH | 2281 | Crotalus | oreganus | lutosus | USA | Utah | Washington |
| SDSNH | 2282 | Crotalus | oreganus | lutosus | USA | Utah | Washington |
| SDSNH | 2283 | Crotalus | oreganus | lutosus | USA | Utah | Washington |
| SDSNH | 2292 | Crotalus | oreganus | helleri | USA | California | San Diego |
| SDSNH | 2315 | Crotalus | viridis | viridis |  |  |  |
| SDSNH | 2331 | Crotalus | oreganus | cerberus | Mexico | Sonora |  |
| SDSNH | 2332 | Crotalus | oreganus | cerberus | Mexico | Sonora |  |
| SDSNH | 2333 | Crotalus | oreganus | cerberus | Mexico | Sonora |  |
| SDSNH | 2340 | Crotalus | oreganus | helleri | USA | California | Santa Barbara |
| SDSNH | 2341 | Crotalus | viridis | viridis | USA | Texas | El Paso |
| SDSNH | 2361 | Crotalus | viridis | viridis | USA | Kansas | Hodgeman |
| SDSNH | 2362 | Crotalus | viridis | viridis | USA | Kansas | Hodgeman |
| SDSNH | 2387 | Crotalus | oreganus | helleri | USA | California | San Diego |
| SDSNH | 2396 | Crotalus | oreganus | helleri | USA | California | San Diego |
| SDSNH | 2397 | Crotalus | oreganus | helleri | USA | California | Santa Barbara |
| SDSNH | 2413 | Crotalus | oreganus | helleri | USA | California | San Diego |
| SDSNH | 2525 | Crotalus | oreganus | helleri | USA | California | San Diego |
| SDSNH | 2594 | Crotalus | oreganus | cerberus | USA | Arizona | Yavapai |
| SDSNH | 2595 | Crotalus | oreganus | cerberus | USA | Arizona | Yavapai |
| SDSNH | 2600 | Crotalus | oreganus | abyssus | USA | Arizona |  |
| SDSNH | 2607 | Crotalus | oreganus | helleri | USA | California | San Diego |
| SDSNH | 2657 | Crotalus | oreganus | abyssus | USA | Arizona | Coconino |
| SDSNH | 2694 | Crotalus | oreganus | cerberus | USA | Arizona | Yavapai |
| SDSNH | 2694 | Crotalus | oreganus | cerberus | USA | Arizona | Yavapai |
| SDSNH | 2696 | Crotalus | oreganus | helleri | USA | California | San Luis Obispo |
| SDSNH | 2752 | Crotalus | oreganus | helleri | USA | California |  |
| SDSNH | 2772 | Crotalus | oreganus | cerberus | USA | Arizona | Yavapai |
| SDSNH | 2800 | Crotalus | oreganus | caliginis | Mexico | Baja California Norte |  |
| SDSNH | 2801 | Crotalus | oreganus | caliginis | Mexico | Baja California Norte |  |
| SDSNH | 2801 | Crotalus | oreganus | caliginis | Mexico | Baja California Norte |  |
| SDSNH | 2802 | Crotalus | oreganus | caliginis | Mexico | Baja California Norte |  |
| SDSNH | 2802 | Crotalus | oreganus | caliginis | Mexico | Baja California Norte |  |
| SDSNH | 2803 | Crotalus | oreganus | caliginis | Mexico | Baja California Norte |  |
| SDSNH | 2804 | Crotalus | oreganus | caliginis | Mexico | Baja California Norte |  |
| SDSNH | 2806 | Crotalus | oreganus | helleri | USA | California | San Diego |
| SDSNH | 2808 | Crotalus | oreganus | helleri | USA | California | Los Angeles |
| SDSNH | 2809 | Crotalus | oreganus | oreganus | USA | Idaho | Nez Pierce |
| SDSNH | 2910 | Crotalus | viridis | nuntius | USA | Arizona | Apache |
| SDSNH | 2913 | Crotalus | oreganus | cerberus | USA | Arizona | Yavapai |
| SDSNH | 2913 | Crotalus | oreganus | cerberus | USA | Arizona | Yavapai |
| SDSNH | 2932 | Crotalus | oreganus | helleri | USA | California | San Diego |
| SDSNH | 2933 | Crotalus | viridis | nuntius | USA | Arizona | Apache |
| SDSNH | 2976 | Crotalus | oreganus | cerberus | USA | Arizona | Yavapai |
| SDSNH | 2977 | Crotalus | oreganus | cerberus | USA | Arizona | Yavapai |
| SDSNH | 2978 | Crotalus | oreganus | cerberus | USA | Arizona | Yavapai |
| SDSNH | 2979 | Crotalus | viridis | nuntius | USA | Arizona | Apache |
| SDSNH | 2980 | Crotalus | viridis | nuntius | USA | Arizona | Apache |
| SDSNH | 2981 | Crotalus | viridis | nuntius | USA | Arizona | Navajo |
| SDSNH | 3041 | Crotalus | oreganus | helleri | USA | California | San Diego |
| SDSNH | 3050 | Crotalus | viridis | nuntius | USA | Arizona | Coconino |
| SDSNH | 3051 | Crotalus | viridis | nuntius | USA | Arizona | Coconino |
| SDSNH | 3052 | Crotalus | viridis | nuntius | USA | Arizona | Apache |
| SDSNH | 3053 | Crotalus | viridis | nuntius | USA | Arizona | Coconino |
| SDSNH | 3054 | Crotalus | viridis | nuntius | USA | Arizona | Coconino |
| SDSNH | 3062 | Crotalus | oreganus | cerberus | USA | Arizona | Yavapai |
| SDSNH | 3063 | Crotalus | oreganus | cerberus | USA | Arizona | Yavapai |
| SDSNH | 3064 | Crotalus | oreganus | cerberus | USA | Arizona | Yavapai |
| SDSNH | 3065 | Crotalus | oreganus | cerberus | USA | Arizona | Yavapai |
| SDSNH | 3083 | Crotalus | oreganus | cerberus | USA | Arizona | Yavapai |
| SDSNH | 3084 | Crotalus | oreganus | cerberus | USA | Arizona | Yavapai |
| SDSNH | 3096 | Crotalus | viridis | nuntius | USA | Arizona | Apache |
| SDSNH | 3097 | Crotalus | viridis | nuntius | USA | Arizona | Apache |
| SDSNH | 3098 | Crotalus | viridis | nuntius | USA | Arizona | Coconino |
| SDSNH | 3100 | Crotalus | viridis | nuntius | USA | Arizona | Apache |
| SDSNH | 3100 | Crotalus | viridis | nuntius | USA | Arizona | Apache |
| SDSNH | 3101 | Crotalus | viridis | nuntius | USA | Arizona | Coconino |
| SDSNH | 3102 | Crotalus | viridis | nuntius | USA | Arizona | Navajo |
| SDSNH | 3103 | Crotalus | viridis | nuntius | USA | Arizona | Navajo |
| SDSNH | 3104 | Crotalus | viridis | nuntius | USA | Arizona | Navajo |
| SDSNH | 3105 | Crotalus | viridis | nuntius | USA | Arizona | Navajo |
| SDSNH | 3165 | Crotalus | viridis | nuntius | USA | Arizona | Coconino |
| SDSNH | 3166 | Crotalus | viridis | nuntius | USA | Arizona | Coconino |
| SDSNH | 3167 | Crotalus | viridis | nuntius | USA | Arizona | Navajo |
| SDSNH | 3177 | Crotalus | oreganus | cerberus | USA | Arizona | Yavapai |
| SDSNH | 3180 | Crotalus | viridis | nuntius | USA | Arizona | Coconino |
| SDSNH | 3181 | Crotalus | viridis | nuntius | USA | Arizona | Coconino |
| SDSNH | 3182 | Crotalus | viridis | nuntius | USA | Arizona | Coconino |
| SDSNH | 3183 | Crotalus | viridis | nuntius | USA | Arizona | Coconino |
| SDSNH | 3184 | Crotalus | viridis | nuntius | USA | Arizona | Coconino |
| SDSNH | 3185 | Crotalus | viridis | nuntius | USA | Arizona | Coconino |
| SDSNH | 3186 | Crotalus | viridis | nuntius | USA | Arizona | Coconino |
| SDSNH | 3187 | Crotalus | viridis | nuntius | USA | Arizona | Navajo |
| SDSNH | 3188 | Crotalus | oreganus | helleri | USA | California | San Diego |
| SDSNH | 3189 | Crotalus | oreganus | helleri | USA | California | San Diego |
| SDSNH | 3190 | Crotalus | viridis | nuntius | USA | Arizona | Yavapai |
| SDSNH | 3191 | Crotalus | viridis | nuntius | USA | Arizona | Coconino |
| SDSNH | 3192 | Crotalus | viridis | nuntius | USA | Arizona | Apache |
| SDSNH | 3193 | Crotalus | viridis | nuntius | USA | Arizona | Apache |
| SDSNH | 3194 | Crotalus | viridis | nuntius | USA | Arizona | Apache |
| SDSNH | 3195 | Crotalus | viridis | nuntius | USA | Arizona | Apache |
| SDSNH | 3196 | Crotalus | viridis | nuntius | USA | Arizona | Apache |
| SDSNH | 3248 | Crotalus | viridis | nuntius | USA | Arizona | Coconino |
| SDSNH | 3249 | Crotalus | viridis | nuntius | USA | Arizona | Coconino |
| SDSNH | 3250 | Crotalus | viridis | nuntius | USA | Arizona | Coconino |
| SDSNH | 3251 | Crotalus | viridis | nuntius | USA | Arizona | Apache |
| SDSNH | 3254 | Crotalus | oreganus | abyssus | USA | Arizona | Coconino |
| SDSNH | 3255 | Crotalus | viridis | nuntius | USA | Arizona | Coconino |
| SDSNH | 3256 | Crotalus | viridis | nuntius | USA | Arizona | Coconino |
| SDSNH | 3257 | Crotalus | viridis | nuntius | USA | Arizona | Coconino |
| SDSNH | 3258 | Crotalus | viridis | nuntius | USA | Arizona | Coconino |
| SDSNH | 3259 | Crotalus | viridis | nuntius | USA | Arizona | Coconino |
| SDSNH | 3260 | Crotalus | viridis | nuntius | USA | Arizona | Navajo |
| SDSNH | 3260 | Crotalus | viridis | nuntius | USA | Arizona | Navajo |
| SDSNH | 3261 | Crotalus | viridis | nuntius | USA | Arizona | Coconino |
| SDSNH | 3262 | Crotalus | viridis | nuntius | USA | Arizona | Coconino |
| SDSNH | 3263 | Crotalus | viridis | nuntius | USA | Arizona | Coconino |
| SDSNH | 3264 | Crotalus | viridis | nuntius | USA | Arizona | Apache |
| SDSNH | 3302 | Crotalus | oreganus | cerberus | USA | Arizona | Mohave |
| SDSNH | 3303 | Crotalus | oreganus | cerberus | USA | Arizona | Mohave |
| SDSNH | 3312 | Crotalus | viridis | nuntius | USA | Arizona | Coconino |
| SDSNH | 3313 | Crotalus | viridis | nuntius | USA | Arizona | Coconino |
| SDSNH | 3313 | Crotalus | viridis | nuntius | USA | Arizona | Coconino |
| SDSNH | 3314 | Crotalus | viridis | nuntius | USA | Arizona | Coconino |
| SDSNH | 3315 | Crotalus | viridis | viridis | USA | New Mexico | Valencia |
| SDSNH | 3316 | Crotalus | viridis | viridis | USA | New Mexico | Valencia |
| SDSNH | 3317 | Crotalus | viridis | nuntius | USA | Arizona | Apache |
| SDSNH | 3318 | Crotalus | viridis | nuntius | USA | Arizona | Navajo |
| SDSNH | 3319 | Crotalus | viridis | nuntius | USA | Arizona | Navajo |
| SDSNH | 3407 | Crotalus | oreganus | oreganus | USA | California | Mono |
| SDSNH | 3435 | Crotalus | oreganus | helleri | USA | California | San Diego |
| SDSNH | 3446 | Crotalus | oreganus | lutosus | USA | Utah | Washington |
| SDSNH | 3452 | Crotalus | oreganus | oreganus | USA | California | Mono |
| SDSNH | 3452 | Crotalus | oreganus | oreganus | USA | California | Mono |
| SDSNH | 3453 | Crotalus | oreganus | helleri | USA | California | San Diego |
| SDSNH | 3454 | Crotalus | oreganus | helleri | USA | California | San Diego |
| SDSNH | 3471 | Crotalus | viridis | nuntius | USA | Arizona | Coconino |
| SDSNH | 3480 | Crotalus | oreganus | oreganus | USA | California | Mono |
| SDSNH | 3483 | Crotalus | oreganus | helleri | USA | California | Kern |
| SDSNH | 3485 | Crotalus | viridis | nuntius | USA | Arizona | Apache |
| SDSNH | 3486 | Crotalus | viridis | nuntius | USA | Arizona | Coconino |
| SDSNH | 3487 | Crotalus | viridis | nuntius | USA | Arizona | Coconino |
| SDSNH | 3488 | Crotalus | viridis | nuntius | USA | Arizona | Coconino |
| SDSNH | 3489 | Crotalus | viridis | nuntius | USA | Arizona | Coconino |
| SDSNH | 3490 | Crotalus | viridis | nuntius | USA | Arizona | Coconino |
| SDSNH | 3491 | Crotalus | viridis | nuntius | USA | Arizona | Coconino |
| SDSNH | 3492 | Crotalus | viridis | nuntius | USA | Arizona | Coconino |
| SDSNH | 3503 | Crotalus | viridis | viridis | USA | Wyoming | Indian |
| SDSNH | 3529 | Crotalus | viridis | nuntius | USA | Arizona | Coconino |
| SDSNH | 3547 | Crotalus | oreganus | helleri | USA | California | San Diego |
| SDSNH | 3548 | Crotalus | oreganus | helleri | USA | California | San Diego |
| SDSNH | 3549 | Crotalus | oreganus | helleri | USA | California | San Diego |
| SDSNH | 3550 | Crotalus | oreganus | helleri | USA | California | San Diego |
| SDSNH | 3551 | Crotalus | oreganus | helleri | USA | California | San Diego |
| SDSNH | 3552 | Crotalus | oreganus | helleri | USA | California | San Diego |
| SDSNH | 3553 | Crotalus | oreganus | helleri | USA | California | San Diego |
| SDSNH | 3554 | Crotalus | oreganus | helleri | USA | California | San Diego |
| SDSNH | 3574 | Crotalus | oreganus | helleri | USA | California | San Diego |
| SDSNH | 3737 | Crotalus | viridis | nuntius | USA | Arizona | Coconino |
| SDSNH | 3738 | Crotalus | viridis | nuntius | USA | Arizona | Coconino |
| SDSNH | 3739 | Crotalus | viridis | nuntius | USA | Arizona | Coconino |
| SDSNH | 3741 | Crotalus | oreganus | helleri | USA | California | San Diego |
| SDSNH | 3748 | Crotalus | viridis | viridis | USA | New Mexico | Bernalillo |
| SDSNH | 3749 | Crotalus | viridis | viridis | USA | New Mexico | Valencia |
| SDSNH | 3805 | Crotalus | oreganus | helleri | USA | California | Los Angeles |
| SDSNH | 4055 | Crotalus | oreganus | lutosus | USA |  |  |
| SDSNH | 4086 | Crotalus | viridis | nuntius | USA | Arizona | Apache |
| SDSNH | 4087 | Crotalus | viridis | nuntius | USA | Arizona | Apache |
| SDSNH | 4088 | Crotalus | viridis | nuntius | USA | Arizona | Apache |
| SDSNH | 4090 | Crotalus | viridis | nuntius | USA | Arizona | Apache |
| SDSNH | 4091 | Crotalus | viridis | nuntius | USA | Arizona | Apache |
| SDSNH | 4092 | Crotalus | viridis | nuntius | USA | Arizona | Apache |
| SDSNH | 4093 | Crotalus | viridis | nuntius | USA | Arizona | Apache |
| SDSNH | 4094 | Crotalus | viridis | nuntius | USA | Arizona | Apache |
| SDSNH | 4095 | Crotalus | viridis | nuntius | USA | Arizona | Apache |
| SDSNH | 4122 | Crotalus | oreganus | helleri | Mexico | Baja California Norte |  |
| SDSNH | 4123 | Crotalus | oreganus | helleri | Mexico | Baja California Norte |  |
| SDSNH | 4124 | Crotalus | oreganus | helleri | Mexico | Baja California Norte |  |
| SDSNH | 4125 | Crotalus | oreganus | helleri | Mexico | Baja California Norte |  |
| SDSNH | 4280 | Crotalus | oreganus | helleri | USA | California | San Diego |
| SDSNH | 4333 | Crotalus | oreganus | helleri | USA | California | San Diego |
| SDSNH | 4335 | Crotalus | oreganus | helleri | USA | California | San Diego |
| SDSNH | 4361 | Crotalus | oreganus | helleri | USA | California | San Diego |
| SDSNH | 4363 | Crotalus | oreganus | cerberus | USA | Arizona | Yavapai |
| SDSNH | 4370 | Crotalus | oreganus | helleri | USA | California | San Diego |
| SDSNH | 4383 | Crotalus | oreganus | helleri | USA | California | Riverside |
| SDSNH | 4384 | Crotalus | oreganus | helleri | USA | California | Riverside |
| SDSNH | 4406 | Crotalus | oreganus | lutosus |  |  |  |
| SDSNH | 4441 | Crotalus | oreganus | cerberus | USA | Arizona | Yavapai |
| SDSNH | 4442 | Crotalus | oreganus | helleri | USA | California | San Diego |
| SDSNH | 4443 | Crotalus | oreganus | helleri | USA | California | San Diego |
| SDSNH | 4444 | Crotalus | oreganus | cerberus | USA | Arizona | Yavapai |
| SDSNH | 4467 | Crotalus | oreganus | lutosus | USA | Idaho | Ada |
| SDSNH | 4468 | Crotalus | oreganus | lutosus | USA | Idaho | Ada |
| SDSNH | 4469 | Crotalus | oreganus | lutosus | USA | Idaho | Ada |
| SDSNH | 4470 | Crotalus | oreganus | lutosus | USA | Idaho | Ada |
| SDSNH | 4471 | Crotalus | oreganus | lutosus | USA | Idaho | Ada |
| SDSNH | 4473 | Crotalus | oreganus | lutosus | USA | Idaho | Ada |
| SDSNH | 4474 | Crotalus | oreganus | lutosus | USA | Idaho | Ada |
| SDSNH | 4475 | Crotalus | oreganus | lutosus | USA | Idaho | Ada |
| SDSNH | 4476 | Crotalus | oreganus | lutosus | USA | Idaho | Ada |
| SDSNH | 4477 | Crotalus | oreganus | lutosus | USA | Idaho | Ada |
| SDSNH | 4479 | Crotalus | oreganus | lutosus | USA | Idaho | Ada |
| SDSNH | 4480 | Crotalus | oreganus | lutosus | USA | Idaho | Ada |
| SDSNH | 4481 | Crotalus | oreganus | lutosus | USA | Idaho | Ada |
| SDSNH | 4482 | Crotalus | oreganus | lutosus | USA | Idaho | Owyhee |
| SDSNH | 4483 | Crotalus | oreganus | lutosus | USA | Idaho | Elmore |
| SDSNH | 4484 | Crotalus | oreganus | lutosus | USA | Idaho | Elmore |
| SDSNH | 4485 | Crotalus | oreganus | lutosus | USA | Idaho | Elmore |
| SDSNH | 4499 | Crotalus | oreganus | oreganus | USA | Washington | Okanogan |
| SDSNH | 4500 | Crotalus | oreganus | oreganus | USA | Washington | Okanogan |
| SDSNH | 4501 | Crotalus | oreganus | oreganus | USA | Washington | Okanogan |
| SDSNH | 4502 | Crotalus | oreganus | oreganus | USA | Washington | Okanogan |
| SDSNH | 4503 | Crotalus | oreganus | oreganus | USA | Washington | Okanogan |
| SDSNH | 4504 | Crotalus | oreganus | oreganus | USA | Washington | Okanogan |
| SDSNH | 4505 | Crotalus | oreganus | oreganus | USA | Washington | Okanogan |
| SDSNH | 4506 | Crotalus | oreganus | oreganus | USA | Washington | Okanogan |
| SDSNH | 4507 | Crotalus | oreganus | oreganus | USA | Washington | Okanogan |
| SDSNH | 4508 | Crotalus | oreganus | oreganus | USA | Washington | Okanogan |
| SDSNH | 4509 | Crotalus | oreganus | oreganus | USA | Washington | Okanogan |
| SDSNH | 4510 | Crotalus | oreganus | oreganus | USA | Washington | Okanogan |
| SDSNH | 4548 | Crotalus | oreganus | oreganus | USA | Washington | Okanogan |
| SDSNH | 4549 | Crotalus | oreganus | oreganus | USA | Washington | Okanogan |
| SDSNH | 4550 | Crotalus | oreganus | oreganus | USA | Washington | Okanogan |
| SDSNH | 4550 | Crotalus | oreganus | oreganus | USA | Washington | Okanogan |
| SDSNH | 4550 | Crotalus | oreganus | oreganus | USA | Washington | Okanogan |
| SDSNH | 4551 | Crotalus | oreganus | oreganus | USA | Washington | Okanogan |
| SDSNH | 4552 | Crotalus | oreganus | oreganus | USA | Washington | Okanogan |
| SDSNH | 4553 | Crotalus | oreganus | oreganus | USA | Washington | Okanogan |
| SDSNH | 4554 | Crotalus | oreganus | oreganus | USA | Washington | Okanogan |
| SDSNH | 4555 | Crotalus | oreganus | oreganus | USA | Washington | Okanogan |
| SDSNH | 4555 | Crotalus | oreganus | oreganus | USA | Washington | Okanogan |
| SDSNH | 4556 | Crotalus | oreganus | oreganus | USA | Washington | Okanogan |
| SDSNH | 4557 | Crotalus | oreganus | oreganus | USA | Washington | Okanogan |
| SDSNH | 4558 | Crotalus | oreganus | oreganus | USA | Washington | Okanogan |
| SDSNH | 4559 | Crotalus | oreganus | oreganus | USA | Washington | Okanogan |
| SDSNH | 4561 | Crotalus | oreganus | oreganus | USA | Washington | Okanogan |
| SDSNH | 4561 | Crotalus | oreganus | oreganus | USA | Washington | Okanogan |
| SDSNH | 4562 | Crotalus | viridis | viridis | USA | Colorado | Weld |
| SDSNH | 4563 | Crotalus | viridis | viridis | USA | Colorado | Weld |
| SDSNH | 4564 | Crotalus | viridis | viridis | USA | Colorado | Weld |
| SDSNH | 4568 | Crotalus | viridis | nuntius | USA | Arizona | Coconino |
| SDSNH | 4709 | Crotalus | oreganus | cerberus | USA | Arizona | Yavapai |
| SDSNH | 4710 | Crotalus | viridis | nuntius | USA | Arizona | Coconino |
| SDSNH | 4711 | Crotalus | viridis | viridis | USA | Texas | El Paso |
| SDSNH | 4728 | Crotalus | oreganus | helleri | USA | California | San Bernardino |
| SDSNH | 4729 | Crotalus | oreganus | oreganus | USA | Oregon | Umatilla |
| SDSNH | 4821 | Crotalus | oreganus | oreganus | USA | Washington | Walla Walla |
| SDSNH | 4822 | Crotalus | oreganus | cerberus | USA | Arizona | Yavapai |
| SDSNH | 4832 | Crotalus | viridis | nuntius | USA | Arizona | Coconino |
| SDSNH | 4833 | Crotalus | viridis | nuntius | USA | Arizona | Coconino |
| SDSNH | 4923 | Crotalus | oreganus | cerberus | USA | Arizona | Yavapai |
| SDSNH | 4924 | Crotalus | oreganus | caliginis | Mexico | Baja California Norte |  |
| SDSNH | 4925 | Crotalus | oreganus | caliginis | Mexico | Baja California Norte |  |
| SDSNH | 4926 | Crotalus | oreganus | caliginis | Mexico | Baja California Norte |  |
| SDSNH | 4928 | Crotalus | viridis | nuntius | USA | Arizona | Coconino |
| SDSNH | 4929 | Crotalus | viridis | nuntius | USA | Arizona | Coconino |
| SDSNH | 4932 | Crotalus | oreganus | cerberus | USA | Arizona | Yavapai |
| SDSNH | 4933 | Crotalus | viridis | nuntius | USA | Arizona | Coconino |
| SDSNH | 4943 | Crotalus | viridis | viridis | USA | South Dakota | Lawrence |
| SDSNH | 4945 | Crotalus | viridis | nuntius | USA | Arizona | Coconino |
| SDSNH | 4946 | Crotalus | viridis | nuntius | USA | Arizona | Coconino |
| SDSNH | 4950 | Crotalus | oreganus | helleri | USA | California | San Diego |
| SDSNH | 4955 | Crotalus | oreganus | oreganus | USA | California | Mono |
| SDSNH | 4956 | Crotalus | oreganus | oreganus | USA | Oregon | Baker |
| SDSNH | 4957 | Crotalus | oreganus | cerberus | USA | Arizona | Yavapai |
| SDSNH | 4974 | Crotalus | oreganus | cerberus | USA | Arizona | Gila |
| SDSNH | 4976 | Crotalus | viridis | viridis | USA | Texas | El Paso |
| SDSNH | 5050 | Crotalus | oreganus | lutosus | USA | Nevada | Churchill |
| SDSNH | 5051 | Crotalus | oreganus | oreganus | USA | California | Mono |
| SDSNH | 5059 | Crotalus | viridis | viridis | USA | Colorado | Weld |
| SDSNH | 5060 | Crotalus | viridis | viridis | USA | New Mexico | San Juan |
| SDSNH | 5061 | Crotalus | viridis | viridis | USA | Colorado | Weld |
| SDSNH | 5069 | Crotalus | oreganus | cerberus | USA | Arizona | Yavapai |
| SDSNH | 5070 | Crotalus | oreganus | cerberus | USA | Arizona | Yavapai |
| SDSNH | 5070 | Crotalus | oreganus | cerberus | USA | Arizona | Yavapai |
| SDSNH | 5166 | Crotalus | oreganus | helleri | USA | California | San Diego |
| SDSNH | 5167 | Crotalus | oreganus | cerberus | USA | Arizona | Yavapai |
| SDSNH | 5168 | Crotalus | oreganus | cerberus | USA | Arizona | Yavapai |
| SDSNH | 5168 | Crotalus | oreganus | cerberus | USA | Arizona | Yavapai |
| SDSNH | 5169 | Crotalus | oreganus | oreganus | USA | California | Mono |
| SDSNH | 5170 | Crotalus | oreganus | oreganus | USA | California | Mono |
| SDSNH | 5276 | Crotalus | oreganus | cerberus | USA | Arizona | Yavapai |
| SDSNH | 5278 | Crotalus | viridis | viridis | USA | New Mexico | Valencia |
| SDSNH | 5279 | Crotalus | viridis | viridis | USA | New Mexico | McKinley |
| SDSNH | 5280 | Crotalus | viridis | viridis | USA | New Mexico | Valencia |
| SDSNH | 5281 | Crotalus | viridis | viridis | USA | New Mexico | Valencia |
| SDSNH | 5282 | Crotalus | viridis | viridis | USA | New Mexico | McKinley |
| SDSNH | 5439 | Crotalus | viridis | nuntius | USA | Arizona | Navajo |
| SDSNH | 5440 | Crotalus | viridis | nuntius | USA | Arizona | Coconino |
| SDSNH | 5441 | Crotalus | viridis | nuntius | USA | Arizona | Navajo |
| SDSNH | 5442 | Crotalus | viridis | nuntius | USA | Arizona | Coconino |
| SDSNH | 5571 | Crotalus | viridis | nuntius | USA | Arizona | Coconino |
| SDSNH | 5838 | Crotalus | oreganus | helleri | USA | California | San Diego |
| SDSNH | 5838 | Crotalus | oreganus | helleri | USA | California | San Diego |
| SDSNH | 5841 | Crotalus | oreganus | helleri | USA | California | San Diego |
| SDSNH | 5843 | Crotalus | oreganus | oreganus | USA | California | Kern |
| SDSNH | 6068 | Crotalus | viridis | nuntius | USA | Arizona | Coconino |
| SDSNH | 6122 | Crotalus | oreganus | cerberus | USA | Arizona | Yavapai |
| SDSNH | 6145 | Crotalus | viridis | viridis | USA | Oklahoma | Cimarron |
| SDSNH | 6359 | Crotalus | viridis | viridis | USA | Colorado | Weld |
| SDSNH | 6360 | Crotalus | viridis | viridis | USA | Colorado | Weld |
| SDSNH | 6361 | Crotalus | viridis | viridis | USA | Colorado | Weld |
| SDSNH | 6362 | Crotalus | viridis | viridis | USA | Colorado | Weld |
| SDSNH | 6363 | Crotalus | viridis | viridis | USA | Colorado | Weld |
| SDSNH | 6364 | Crotalus | viridis | viridis | USA | Colorado | Weld |
| SDSNH | 6365 | Crotalus | viridis | viridis | USA | Colorado | Weld |
| SDSNH | 6366 | Crotalus | viridis | viridis | USA | Colorado | Weld |
| SDSNH | 6367 | Crotalus | viridis | viridis | USA | Colorado | Weld |
| SDSNH | 6368 | Crotalus | viridis | viridis | USA | Colorado | Weld |
| SDSNH | 6369 | Crotalus | viridis | viridis | USA | Colorado | Weld |
| SDSNH | 6370 | Crotalus | viridis | viridis | USA | Colorado | Weld |
| SDSNH | 6371 | Crotalus | viridis | viridis | USA | Colorado | Weld |
| SDSNH | 6372 | Crotalus | viridis | viridis | USA | Colorado | Weld |
| SDSNH | 6373 | Crotalus | viridis | viridis | USA | Colorado | Weld |
| SDSNH | 6374 | Crotalus | viridis | viridis | USA | Colorado | Weld |
| SDSNH | 6375 | Crotalus | viridis | viridis | USA | Colorado | Weld |
| SDSNH | 6376 | Crotalus | viridis | viridis | USA | Colorado | Weld |
| SDSNH | 6377 | Crotalus | viridis | viridis | USA | Colorado | Weld |
| SDSNH | 6378 | Crotalus | viridis | viridis | USA | Colorado | Weld |
| SDSNH | 6379 | Crotalus | viridis | viridis | USA | Colorado | Weld |
| SDSNH | 6380 | Crotalus | viridis | viridis | USA | Colorado | Weld |
| SDSNH | 6381 | Crotalus | viridis | viridis | USA | Colorado | Weld |
| SDSNH | 6382 | Crotalus | viridis | viridis | USA | Colorado | Weld |
| SDSNH | 6383 | Crotalus | viridis | viridis | USA | Colorado | Weld |
| SDSNH | 6384 | Crotalus | viridis | viridis | USA | Colorado | Weld |
| SDSNH | 6385 | Crotalus | viridis | viridis | USA | Colorado | Weld |
| SDSNH | 6386 | Crotalus | viridis | viridis | USA | Colorado | Weld |
| SDSNH | 6387 | Crotalus | viridis | viridis | USA | Colorado | Weld |
| SDSNH | 6388 | Crotalus | viridis | viridis | USA | Colorado | Weld |
| SDSNH | 6389 | Crotalus | viridis | viridis | USA | Colorado | Weld |
| SDSNH | 6389 | Crotalus | viridis | viridis | USA | Colorado | Weld |
| SDSNH | 6390 | Crotalus | viridis | viridis | USA | Colorado | Weld |
| SDSNH | 6391 | Crotalus | viridis | viridis | USA | Colorado | Weld |
| SDSNH | 6392 | Crotalus | viridis | viridis | USA | Colorado | Weld |
| SDSNH | 6393 | Crotalus | viridis | viridis | USA | Colorado | Weld |
| SDSNH | 6394 | Crotalus | viridis | viridis | USA | Colorado | Weld |
| SDSNH | 6395 | Crotalus | viridis | viridis | USA | Colorado | Weld |
| SDSNH | 6396 | Crotalus | viridis | viridis | USA | Colorado | Weld |
| SDSNH | 6397 | Crotalus | viridis | viridis | USA | Colorado | Weld |
| SDSNH | 6399 | Crotalus | viridis | viridis | USA | Colorado | Weld |
| SDSNH | 6400 | Crotalus | viridis | viridis | USA | Colorado | Weld |
| SDSNH | 6401 | Crotalus | viridis | viridis | USA | Colorado | Weld |
| SDSNH | 6402 | Crotalus | viridis | viridis | USA | Colorado | Weld |
| SDSNH | 6403 | Crotalus | viridis | viridis | USA | Colorado | Weld |
| SDSNH | 6549 | Crotalus | viridis | viridis | USA | Colorado | Weld |
| SDSNH | 6558 | Crotalus | viridis | viridis | USA | Colorado | Weld |
| SDSNH | 6559 | Crotalus | viridis | viridis | USA | Colorado | Weld |
| SDSNH | 6562 | Crotalus | viridis | viridis | USA | Colorado | Weld |
| SDSNH | 6593 | Crotalus | viridis | viridis | USA | Colorado | Weld |
| SDSNH | 6775 | Crotalus | oreganus | helleri | USA | California | San Diego |
| SDSNH | 7096 | Crotalus | oreganus | helleri | USA | California | San Diego |
| SDSNH | 7534 | Crotalus | oreganus | helleri | USA | California | San Diego |
| SDSNH | 7537 | Crotalus | oreganus | caliginis | Mexico | Baja California Norte |  |
| SDSNH | 7537 | Crotalus | oreganus | caliginis | Mexico | Baja California Norte |  |
| SDSNH | 7539 | Crotalus | oreganus | caliginis | Mexico | Baja California Norte |  |
| SDSNH | 7540 | Crotalus | oreganus | caliginis | Mexico | Baja California Norte |  |
| SDSNH | 8050 | Crotalus | oreganus | helleri | USA | California | San Diego |
| SDSNH | 8105 | Crotalus | oreganus | oreganus | USA | Idaho | Adams |
| SDSNH | 8119 | Crotalus | oreganus | helleri | USA | California | San Diego |
| SDSNH | 8177 | Crotalus | viridis | viridis | USA | Colorado | Weld |
| SDSNH | 8178 | Crotalus | viridis | viridis | USA | Colorado | Weld |
| SDSNH | 8179 | Crotalus | viridis | viridis | USA | Colorado | Weld |
| SDSNH | 8180 | Crotalus | viridis | viridis | USA | Colorado | Weld |
| SDSNH | 8181 | Crotalus | viridis | viridis | USA | Colorado | Weld |
| SDSNH | 8182 | Crotalus | viridis | viridis | USA | Colorado | Weld |
| SDSNH | 8183 | Crotalus | viridis | viridis | USA | Colorado | Weld |
| SDSNH | 8188 | Crotalus | viridis | viridis | USA | Colorado | Weld |
| SDSNH | 8189 | Crotalus | viridis | viridis | USA | Colorado | Weld |
| SDSNH | 8190 | Crotalus | viridis | viridis | USA | Colorado | Weld |
| SDSNH | 8191 | Crotalus | viridis | viridis | USA | Colorado | Weld |
| SDSNH | 8192 | Crotalus | viridis | viridis | USA | Colorado | Weld |
| SDSNH | 8193 | Crotalus | viridis | viridis | USA | Colorado | Weld |
| SDSNH | 8194 | Crotalus | viridis | viridis | USA | Colorado | Weld |
| SDSNH | 8195 | Crotalus | viridis | viridis | USA | Colorado | Weld |
| SDSNH | 8196 | Crotalus | viridis | viridis | USA | Colorado | Weld |
| SDSNH | 8197 | Crotalus | viridis | viridis | USA | Colorado | Weld |
| SDSNH | 8198 | Crotalus | viridis | viridis | USA | Colorado | Weld |
| SDSNH | 8199 | Crotalus | viridis | viridis | USA | Colorado | Weld |
| SDSNH | 8200 | Crotalus | viridis | viridis | USA | Colorado | Weld |
| SDSNH | 8201 | Crotalus | viridis | viridis | USA | Colorado | Weld |
| SDSNH | 8202 | Crotalus | viridis | viridis | USA | Colorado | Weld |
| SDSNH | 8203 | Crotalus | viridis | viridis | USA | Colorado | Weld |
| SDSNH | 8204 | Crotalus | viridis | viridis | USA | Colorado | Weld |
| SDSNH | 8205 | Crotalus | viridis | viridis | USA | Colorado | Weld |
| SDSNH | 8206 | Crotalus | viridis | viridis | USA | Colorado | Weld |
| SDSNH | 8207 | Crotalus | viridis | viridis | USA | Colorado | Weld |
| SDSNH | 8208 | Crotalus | viridis | viridis | USA | Colorado | Weld |
| SDSNH | 8209 | Crotalus | viridis | viridis | USA | Colorado | Weld |
| SDSNH | 8210 | Crotalus | viridis | viridis | USA | Colorado | Weld |
| SDSNH | 8211 | Crotalus | viridis | viridis | USA | Colorado | Weld |
| SDSNH | 8212 | Crotalus | viridis | viridis | USA | Colorado | Weld |
| SDSNH | 8213 | Crotalus | viridis | viridis | USA | Colorado | Weld |
| SDSNH | 8214 | Crotalus | viridis | viridis | USA | Colorado | Weld |
| SDSNH | 8216 | Crotalus | viridis | viridis | USA | Colorado | Weld |
| SDSNH | 8217 | Crotalus | viridis | viridis | USA | Colorado | Weld |
| SDSNH | 8217 | Crotalus | viridis | viridis | USA | Colorado | Weld |
| SDSNH | 8218 | Crotalus | viridis | viridis | USA | Colorado | Weld |
| SDSNH | 8219 | Crotalus | viridis | viridis | USA | Colorado | Weld |
| SDSNH | 8220 | Crotalus | viridis | viridis | USA | Colorado | Weld |
| SDSNH | 8221 | Crotalus | viridis | viridis | USA | Colorado | Weld |
| SDSNH | 8222 | Crotalus | viridis | viridis | USA | Colorado | Weld |
| SDSNH | 8223 | Crotalus | viridis | viridis | USA | Colorado | Weld |
| SDSNH | 8224 | Crotalus | viridis | viridis | USA | Colorado | Weld |
| SDSNH | 8225 | Crotalus | viridis | viridis | USA | Colorado | Weld |
| SDSNH | 8226 | Crotalus | viridis | viridis | USA | Colorado | Weld |
| SDSNH | 8227 | Crotalus | viridis | viridis | USA | Colorado | Weld |
| SDSNH | 8228 | Crotalus | viridis | viridis | USA | Colorado | Weld |
| SDSNH | 8229 | Crotalus | viridis | viridis | USA | Colorado | Weld |
| SDSNH | 8230 | Crotalus | viridis | viridis | USA | Colorado | Weld |
| SDSNH | 8231 | Crotalus | viridis | viridis | USA | Colorado | Weld |
| SDSNH | 8232 | Crotalus | viridis | viridis | USA | Colorado | Weld |
| SDSNH | 8233 | Crotalus | viridis | viridis | USA | Colorado | Weld |
| SDSNH | 8234 | Crotalus | viridis | viridis | USA | Colorado | Weld |
| SDSNH | 8235 | Crotalus | viridis | viridis | USA | Colorado | Weld |
| SDSNH | 8236 | Crotalus | viridis | viridis | USA | Colorado | Weld |
| SDSNH | 8237 | Crotalus | viridis | viridis | USA | Colorado | Weld |
| SDSNH | 8238 | Crotalus | viridis | viridis | USA | Colorado | Weld |
| SDSNH | 8239 | Crotalus | viridis | viridis | USA | Colorado | Weld |
| SDSNH | 8240 | Crotalus | viridis | viridis | USA | Colorado | Weld |
| SDSNH | 8241 | Crotalus | viridis | viridis | USA | Colorado | Weld |
| SDSNH | 8242 | Crotalus | viridis | viridis | USA | Colorado | Weld |
| SDSNH | 8243 | Crotalus | viridis | viridis | USA | Colorado | Weld |
| SDSNH | 8244 | Crotalus | viridis | viridis | USA | Colorado | Weld |
| SDSNH | 8245 | Crotalus | viridis | viridis | USA | Colorado | Weld |
| SDSNH | 8246 | Crotalus | viridis | viridis | USA | Colorado | Weld |
| SDSNH | 8247 | Crotalus | viridis | viridis | USA | Colorado | Weld |
| SDSNH | 8248 | Crotalus | viridis | viridis | USA | Colorado | Weld |
| SDSNH | 8249 | Crotalus | viridis | viridis | USA | Colorado | Weld |
| SDSNH | 8250 | Crotalus | viridis | viridis | USA | Colorado | Weld |
| SDSNH | 8251 | Crotalus | viridis | viridis | USA | Colorado | Weld |
| SDSNH | 8252 | Crotalus | viridis | viridis | USA | Colorado | Weld |
| SDSNH | 8253 | Crotalus | viridis | viridis | USA | Colorado | Weld |
| SDSNH | 8254 | Crotalus | viridis | viridis | USA | Colorado | Weld |
| SDSNH | 8255 | Crotalus | viridis | viridis | USA | Colorado | Weld |
| SDSNH | 8256 | Crotalus | viridis | viridis | USA | Colorado | Weld |
| SDSNH | 8314 | Crotalus | viridis | viridis | USA | Colorado | Weld |
| SDSNH | 8315 | Crotalus | viridis | viridis | USA | Colorado | Weld |
| SDSNH | 8316 | Crotalus | viridis | viridis | USA | Colorado | Weld |
| SDSNH | 8317 | Crotalus | viridis | viridis | USA | Colorado | Weld |
| SDSNH | 8319 | Crotalus | viridis | viridis | USA | Colorado | Weld |
| SDSNH | 8320 | Crotalus | viridis | viridis | USA | Colorado | Weld |
| SDSNH | 8321 | Crotalus | viridis | viridis | USA | Colorado | Weld |
| SDSNH | 8322 | Crotalus | viridis | viridis | USA | Colorado | Weld |
| SDSNH | 8443 | Crotalus | oreganus | oreganus | USA | Washington | Okanogan |
| SDSNH | 8444 | Crotalus | oreganus | oreganus | USA | Washington | Okanogan |
| SDSNH | 8445 | Crotalus | oreganus | oreganus | USA | Washington | Okanogan |
| SDSNH | 8446 | Crotalus | oreganus | oreganus | USA | Washington | Okanogan |
| SDSNH | 8448 | Crotalus | oreganus | oreganus | USA | Washington | Okanogan |
| SDSNH | 8449 | Crotalus | oreganus | oreganus | USA | Washington | Okanogan |
| SDSNH | 8450 | Crotalus | oreganus | oreganus | USA | Washington | Okanogan |
| SDSNH | 8451 | Crotalus | oreganus | oreganus | USA | Washington | Okanogan |
| SDSNH | 8452 | Crotalus | oreganus | oreganus | USA | Washington | Okanogan |
| SDSNH | 8452 | Crotalus | oreganus | oreganus | USA | Washington | Okanogan |
| SDSNH | 8454 | Crotalus | oreganus | oreganus | USA | Washington | Okanogan |
| SDSNH | 8455 | Crotalus | oreganus | oreganus | USA | Washington | Okanogan |
| SDSNH | 8456 | Crotalus | oreganus | oreganus | USA | Washington | Okanogan |
| SDSNH | 8457 | Crotalus | oreganus | oreganus | USA | Washington | Okanogan |
| SDSNH | 8458 | Crotalus | oreganus | oreganus | USA | Washington | Okanogan |
| SDSNH | 8459 | Crotalus | oreganus | oreganus | USA | Washington | Okanogan |
| SDSNH | 8460 | Crotalus | oreganus | oreganus | USA | Washington | Okanogan |
| SDSNH | 8461 | Crotalus | oreganus | oreganus | USA | Washington | Okanogan |
| SDSNH | 8462 | Crotalus | oreganus | oreganus | USA | Washington | Okanogan |
| SDSNH | 8478 | Crotalus | oreganus | oreganus | USA | Idaho | Adams |
| SDSNH | 8479 | Crotalus | oreganus | oreganus | USA | Idaho | Adams |
| SDSNH | 8480 | Crotalus | oreganus | oreganus | USA | Idaho | Adams |
| SDSNH | 8480 | Crotalus | oreganus | oreganus | USA | Idaho | Adams |
| SDSNH | 8481 | Crotalus | oreganus | oreganus | USA | Idaho | Adams |
| SDSNH | 8482 | Crotalus | oreganus | oreganus | USA | Idaho | Adams |
| SDSNH | 8483 | Crotalus | oreganus | oreganus | USA | Idaho | Adams |
| SDSNH | 8484 | Crotalus | oreganus | oreganus | USA | Idaho | Adams |
| SDSNH | 8485 | Crotalus | oreganus | oreganus | USA | Idaho | Adams |
| SDSNH | 8485 | Crotalus | oreganus | oreganus | USA | Idaho | Adams |
| SDSNH | 8485 | Crotalus | oreganus | oreganus | USA | Idaho | Adams |
| SDSNH | 8486 | Crotalus | oreganus | oreganus | USA | Idaho | Adams |
| SDSNH | 8487 | Crotalus | oreganus | oreganus | USA | Idaho | Adams |
| SDSNH | 8488 | Crotalus | oreganus | oreganus | USA | Idaho | Adams |
| SDSNH | 8489 | Crotalus | oreganus | oreganus | USA | Idaho | Adams |
| SDSNH | 8490 | Crotalus | oreganus | oreganus | USA | Idaho | Adams |
| SDSNH | 8491 | Crotalus | oreganus | oreganus | USA | Idaho | Adams |
| SDSNH | 8492 | Crotalus | oreganus | oreganus | USA | Idaho | Adams |
| SDSNH | 8493 | Crotalus | oreganus | oreganus | USA | Idaho | Adams |
| SDSNH | 8494 | Crotalus | oreganus | oreganus | USA | Idaho | Adams |
| SDSNH | 8496 | Crotalus | oreganus | oreganus | USA | Idaho | Adams |
| SDSNH | 8497 | Crotalus | oreganus | oreganus | USA | Idaho | Adams |
| SDSNH | 8498 | Crotalus | oreganus | oreganus | USA | Idaho | Adams |
| SDSNH | 8499 | Crotalus | oreganus | oreganus | USA | Idaho | Adams |
| SDSNH | 8500 | Crotalus | oreganus | helleri | USA | California | San Bernardino |
| SDSNH | 8510 | Crotalus | oreganus | helleri | USA | California | Los Angeles |
| SDSNH | 8554 | Crotalus | oreganus | oreganus | USA | California | Kern |
| SDSNH | 8555 | Crotalus | oreganus | oreganus | USA | California | Kern |
| SDSNH | 8615 | Crotalus | oreganus | helleri | USA | California | San Diego |
| SDSNH | 8630 | Crotalus | oreganus | oreganus | USA | Washington | Okanogan |
| SDSNH | 8631 | Crotalus | oreganus | oreganus | USA | Washington | Okanogan |
| SDSNH | 8632 | Crotalus | oreganus | oreganus | USA | Washington | Okanogan |
| SDSNH | 8633 | Crotalus | oreganus | oreganus | USA | Washington | Okanogan |
| SDSNH | 8634 | Crotalus | oreganus | oreganus | USA | Washington | Okanogan |
| SDSNH | 8635 | Crotalus | oreganus | oreganus | USA | Washington | Okanogan |
| SDSNH | 8636 | Crotalus | oreganus | oreganus | USA | Washington | Okanogan |
| SDSNH | 8637 | Crotalus | oreganus | oreganus | USA | Washington | Okanogan |
| SDSNH | 8638 | Crotalus | oreganus | oreganus | USA | Washington | Okanogan |
| SDSNH | 8639 | Crotalus | oreganus | oreganus | USA | Washington | Okanogan |
| SDSNH | 8640 | Crotalus | oreganus | oreganus | USA | Washington | Okanogan |
| SDSNH | 8641 | Crotalus | oreganus | oreganus | USA | Washington | Okanogan |
| SDSNH | 8642 | Crotalus | oreganus | oreganus | USA | Washington | Okanogan |
| SDSNH | 8643 | Crotalus | oreganus | oreganus | USA | Washington | Okanogan |
| SDSNH | 8644 | Crotalus | oreganus | oreganus | USA | Washington | Okanogan |
| SDSNH | 8646 | Crotalus | oreganus | helleri | USA | California | San Diego |
| SDSNH | 8647 | Crotalus | oreganus | oreganus | USA | California | Madera |
| SDSNH | 8648 | Crotalus | oreganus | oreganus | USA | California | Madera |
| SDSNH | 8649 | Crotalus | oreganus | lutosus | USA | Idaho | Canyon |
| SDSNH | 8650 | Crotalus | oreganus | lutosus | USA | Idaho | Canyon |
| SDSNH | 8651 | Crotalus | oreganus | lutosus | USA | Idaho | Canyon |
| SDSNH | 8652 | Crotalus | viridis | viridis | USA | Colorado | Weld |
| SDSNH | 8744 | Crotalus | oreganus | oreganus | USA | California | Madera |
| SDSNH | 8745 | Crotalus | oreganus | oreganus | USA | California | Madera |
| SDSNH | 8746 | Crotalus | oreganus | helleri | USA | California | San Bernardino |
| SDSNH | 8747 | Crotalus | oreganus | cerberus | USA | Arizona | Yavapai |
| SDSNH | 8748 | Crotalus | viridis | viridis | USA | Colorado | Weld |
| SDSNH | 8749 | Crotalus | viridis | viridis | USA | Colorado | Weld |
| SDSNH | 8750 | Crotalus | viridis | viridis | USA | Colorado | Weld |
| SDSNH | 8751 | Crotalus | viridis | viridis | USA | Colorado | Weld |
| SDSNH | 8752 | Crotalus | viridis | viridis | USA | Colorado | Weld |
| SDSNH | 8753 | Crotalus | viridis | nuntius | USA | Arizona | Navajo |
| SDSNH | 8754 | Crotalus | viridis | nuntius | USA | Arizona | Coconino |
| SDSNH | 8755 | Crotalus | viridis | nuntius | USA | Arizona | Apache |
| SDSNH | 8756 | Crotalus | viridis | nuntius | USA | Arizona | Apache |
| SDSNH | 8802 | Crotalus | oreganus | oreganus | USA | California | Madera |
| SDSNH | 9200 | Crotalus | oreganus | oreganus | USA | California | Madera |
| SDSNH | 9201 | Crotalus | oreganus | oreganus | USA | California | Madera |
| SDSNH | 9219 | Crotalus | viridis | viridis | USA | New Mexico | Valencia |
| SDSNH | 9257 | Crotalus | viridis | viridis | USA | New Mexico | McKinley |
| SDSNH | 9258 | Crotalus | viridis | viridis | USA | New Mexico | Valencia |
| SDSNH | 9259 | Crotalus | viridis | viridis | USA | New Mexico | Valencia |
| SDSNH | 9260 | Crotalus | viridis | viridis | USA | New Mexico | Valencia |
| SDSNH | 9261 | Crotalus | oreganus | oreganus | USA | California | Pulmas |
| SDSNH | 9262 | Crotalus | oreganus | oreganus | USA | California | Pulmas |
| SDSNH | 9264 | Crotalus | oreganus | oreganus | USA | California | Madera |
| SDSNH | 9265 | Crotalus | oreganus | oreganus | USA | California | Madera |
| SDSNH | 9273 | Crotalus | viridis | viridis | USA | Colorado | Yuma |
| SDSNH | 9275 | Crotalus | oreganus | concolor | USA | Colorado | Montrose |
| SDSNH | 9277 | Crotalus | oreganus | lutosus | USA | Nevada | Lander |
| SDSNH | 9334 | Crotalus | viridis | viridis | USA | New Mexico | Rio Arriba |
| SDSNH | 9335 | Crotalus | viridis | viridis | USA | New Mexico | Valencia |
| SDSNH | 9336 | Crotalus | oreganus | oreganus | USA | Washington | Lincoln |
| SDSNH | 9337 | Crotalus | oreganus | oreganus | USA | Washington | Lincoln |
| SDSNH | 9352 | Crotalus | oreganus | oreganus | USA | California | Mariposa |
| SDSNH | 9353 | Crotalus | oreganus | oreganus | USA | California | Mariposa |
| SDSNH | 9354 | Crotalus | oreganus | oreganus | USA | California | Mariposa |
| SDSNH | 9524 | Crotalus | oreganus | abyssus | USA | Arizona | Coconino |
| SDSNH | 9528 | Crotalus | viridis | viridis | USA | Montana | Wheatland |
| SDSNH | 9529 | Crotalus | viridis | viridis | USA | Montana | Wheatland |
| SDSNH | 9531 | Crotalus | viridis | viridis | USA | Montana | Toole |
| SDSNH | 9532 | Crotalus | viridis | viridis | USA | Montana | Toole |
| SDSNH | 9533 | Crotalus | oreganus | oreganus | USA | Oregon | Jackson |
| SDSNH | 9537 | Crotalus | oreganus | oreganus | USA | California | Kern |
| SDSNH | 9538 | Crotalus | oreganus | helleri | USA | California | San Diego |
| SDSNH | 9613 | Crotalus | viridis | viridis | USA | South Dakota | Stanley |
| SDSNH | 9614 | Crotalus | viridis | viridis | USA | South Dakota | Stanley |
| SDSNH | 9615 | Crotalus | viridis | viridis | USA | South Dakota | Stanley |
| SDSNH | 9632 | Crotalus | viridis | viridis | USA | South Dakota | Stanley |
| SDSNH | 9633 | Crotalus | viridis | viridis | USA | South Dakota | Stanley |
| SDSNH | 9634 | Crotalus | viridis | viridis | USA | South Dakota | Stanley |
| SDSNH | 9635 | Crotalus | viridis | viridis | USA | South Dakota | Stanley |
| SDSNH | 9636 | Crotalus | viridis | viridis | USA | South Dakota | Stanley |
| SDSNH | 9637 | Crotalus | viridis | viridis | USA | South Dakota | Stanley |
| SDSNH | 9638 | Crotalus | viridis | viridis | USA | South Dakota | Stanley |
| SDSNH | 9639 | Crotalus | viridis | viridis | USA | South Dakota | Stanley |
| SDSNH | 9640 | Crotalus | viridis | viridis | USA | South Dakota | Stanley |
| SDSNH | 9652 | Crotalus | viridis | viridis | USA | South Dakota | Stanley |
| SDSNH | 9653 | Crotalus | viridis | viridis | USA | South Dakota | Stanley |
| SDSNH | 9654 | Crotalus | viridis | viridis | USA | South Dakota | Stanley |
| SDSNH | 9655 | Crotalus | viridis | viridis | USA | South Dakota | Stanley |
| SDSNH | 9656 | Crotalus | viridis | viridis | USA | South Dakota | Stanley |
| SDSNH | 9657 | Crotalus | viridis | viridis | USA | South Dakota | Stanley |
| SDSNH | 9658 | Crotalus | viridis | viridis | USA | South Dakota | Stanley |
| SDSNH | 9659 | Crotalus | viridis | viridis | USA | South Dakota | Stanley |
| SDSNH | 9660 | Crotalus | viridis | viridis | USA | South Dakota | Stanley |
| SDSNH | 9661 | Crotalus | viridis | viridis | USA | South Dakota | Stanley |
| SDSNH | 9662 | Crotalus | viridis | viridis | USA | South Dakota | Stanley |
| SDSNH | 9663 | Crotalus | viridis | viridis | USA | South Dakota | Stanley |
| SDSNH | 9664 | Crotalus | viridis | viridis | USA | South Dakota | Stanley |
| SDSNH | 9665 | Crotalus | viridis | viridis | USA | South Dakota | Stanley |
| SDSNH | 9666 | Crotalus | viridis | viridis | USA | South Dakota | Stanley |
| SDSNH | 9667 | Crotalus | viridis | viridis | USA | South Dakota | Stanley |
| SDSNH | 9668 | Crotalus | viridis | viridis | USA | South Dakota | Stanley |
| SDSNH | 9669 | Crotalus | viridis | viridis | USA | South Dakota | Stanley |
| SDSNH | 9670 | Crotalus | viridis | viridis | USA | South Dakota | Stanley |
| SDSNH | 9671 | Crotalus | viridis | viridis | USA | South Dakota | Stanley |
| SDSNH | 9688 | Crotalus | viridis | viridis | USA | South Dakota | Stanley |
| SDSNH | 9689 | Crotalus | viridis | viridis | USA | South Dakota | Stanley |
| SDSNH | 9690 | Crotalus | viridis | viridis | USA | South Dakota | Stanley |
| SDSNH | 9691 | Crotalus | viridis | viridis | USA | South Dakota | Stanley |
| SDSNH | 9692 | Crotalus | viridis | viridis | USA | South Dakota | Stanley |
| SDSNH | 9693 | Crotalus | viridis | viridis | USA | South Dakota | Stanley |
| SDSNH | 9694 | Crotalus | viridis | viridis | USA | South Dakota | Stanley |
| SDSNH | 9695 | Crotalus | viridis | viridis | USA | South Dakota | Stanley |
| SDSNH | 9696 | Crotalus | viridis | viridis | USA | South Dakota | Stanley |
| SDSNH | 9697 | Crotalus | viridis | viridis | USA | South Dakota | Stanley |
| SDSNH | 9698 | Crotalus | viridis | viridis | USA | South Dakota | Stanley |
| SDSNH | 9699 | Crotalus | viridis | viridis | USA | South Dakota | Stanley |
| SDSNH | 9700 | Crotalus | viridis | viridis | USA | South Dakota | Stanley |
| SDSNH | 9701 | Crotalus | viridis | viridis | USA | South Dakota | Stanley |
| SDSNH | 9800 | Crotalus | viridis | viridis | USA | South Dakota | Stanley |
| SDSNH | 9801 | Crotalus | viridis | viridis | USA | South Dakota | Stanley |
| SDSNH | 9802 | Crotalus | viridis | viridis | USA | South Dakota | Stanley |
| SDSNH | 9803 | Crotalus | viridis | viridis | USA | South Dakota | Stanley |
| SDSNH | 9804 | Crotalus | viridis | viridis | USA | South Dakota | Stanley |
| SDSNH | 10036 | Crotalus | oreganus | helleri | Mexico | Baja California |  |
| SDSNH | 10037 | Crotalus | oreganus | helleri | Mexico | Baja California |  |
| SDSNH | 10340 | Crotalus | oreganus | helleri | Mexico | Baja California Norte |  |
| SDSNH | 10537 | Crotalus | oreganus | helleri | USA | California | San Diego |
| SDSNH | 10543 | Crotalus | oreganus | helleri | USA | California | San Diego |
| SDSNH | 10548 | Crotalus | oreganus | helleri | USA | California | San Diego |
| SDSNH | 10914 | Crotalus | oreganus | helleri | USA | California | San Diego |
| SDSNH | 11177 | Crotalus | oreganus | caliginis | Mexico | Baja California Norte |  |
| SDSNH | 11178 | Crotalus | oreganus | caliginis | Mexico | Baja California Norte |  |
| SDSNH | 11179 | Crotalus | oreganus | caliginis | Unknown | Unknown | Unknown |
| SDSNH | 11180 | Crotalus | oreganus | caliginis | Unknown | Unknown | Unknown |
| SDSNH | 11180 | Crotalus | oreganus | caliginis | Unknown | Unknown | Unknown |
| SDSNH | 11551 | Crotalus | oreganus | helleri | USA | California | Riverside |
| SDSNH | 11920 | Crotalus | oreganus | helleri | USA | California | Riverside |
| SDSNH | 13711 | Crotalus | oreganus | caliginis | Mexico | Baja California Norte |  |
| SDSNH | 13712 | Crotalus | oreganus | caliginis | Mexico | Baja California Norte |  |
| SDSNH | 13713 | Crotalus | oreganus | caliginis | Mexico | Baja California Norte |  |
| SDSNH | 13713 | Crotalus | oreganus | caliginis | Mexico | Baja California Norte |  |
| SDSNH | 13714 | Crotalus | oreganus | caliginis | Mexico | Baja California Norte |  |
| SDSNH | 13714 | Crotalus | oreganus | caliginis | Mexico | Baja California Norte |  |
| SDSNH | 13714 | Crotalus | oreganus | caliginis | Mexico | Baja California Norte |  |
| SDSNH | 13715 | Crotalus | oreganus | caliginis | Mexico | Baja California Norte |  |
| SDSNH | 16009 | Crotalus | oreganus | helleri | USA | California | San Diego |
| SDSNH | 16259 | Crotalus | oreganus | cerberus | USA | Arizona | Pima |
| SDSNH | 16259 | Crotalus | oreganus | cerberus | USA | Arizona | Pima |
| SDSNH | 16260 | Crotalus | oreganus | cerberus | USA | Arizona | Pima |
| SDSNH | 16261 | Crotalus | oreganus | cerberus | USA | Arizona | Pima |
| SDSNH | 16262 | Crotalus | oreganus | cerberus | USA | Arizona | Pima |
| SDSNH | 16267 | Crotalus | oreganus | lutosus | USA | California | Mono |
| SDSNH | 16268 | Crotalus | oreganus | lutosus | USA | California | Mono |
| SDSNH | 16533 | Crotalus | oreganus | helleri | USA | California | San Diego |
| SDSNH | 16701 | Crotalus | oreganus | helleri | USA | California | San Diego |
| SDSNH | 17032 | Crotalus | oreganus | helleri | USA | California | San Diego |
| SDSNH | 17138 | Crotalus | oreganus | lutosus | USA | Arizona | Mohave |
| SDSNH | 17139 | Crotalus | oreganus | lutosus | USA | Utah | Washington |
| SDSNH | 17140 | Crotalus | oreganus | lutosus | USA | Arizona | Coconino |
| SDSNH | 17176 | Crotalus | oreganus | helleri | USA | California | Los Angeles |
| SDSNH | 17372 | Crotalus | oreganus | helleri | USA | California | San Diego |
| SDSNH | 17578 | Crotalus | oreganus | cerberus | USA | Arizona | Apache |
| SDSNH | 18547 | Crotalus | oreganus | helleri | Mexico | Baja California Norte |  |
| SDSNH | 18731 | Crotalus | oreganus | helleri | Mexico | Baja California Norte |  |
| SDSNH | 19211 | Crotalus | oreganus | helleri | Mexico | Baja California Norte |  |
| SDSNH | 19571 | Crotalus | oreganus | helleri | USA | California | Los Angeles |
| SDSNH | 19692 | Crotalus | oreganus | helleri | Mexico | Baja California Norte |  |
| SDSNH | 20077 | Crotalus | oreganus | caliginis | Mexico | Baja California Norte |  |
| SDSNH | 20078 | Crotalus | oreganus | caliginis | Mexico | Baja California Norte |  |
| SDSNH | 20081 | Crotalus | oreganus | oreganus | USA | Washington | Okanogan |
| SDSNH | 20082 | Crotalus | oreganus | oreganus | USA | Washington | Okanogan |
| SDSNH | 20083 | Crotalus | oreganus | oreganus | USA | Washington | Okanogan |
| SDSNH | 20084 | Crotalus | oreganus | oreganus | USA | Washington | Okanogan |
| SDSNH | 20085 | Crotalus | oreganus | oreganus | USA | Washington | Okanogan |
| SDSNH | 20086 | Crotalus | oreganus | oreganus | USA | Washington | Okanogan |
| SDSNH | 20087 | Crotalus | oreganus | oreganus | USA | Washington | Okanogan |
| SDSNH | 20088 | Crotalus | oreganus | oreganus | USA | Washington | Okanogan |
| SDSNH | 20089 | Crotalus | oreganus | oreganus | USA | Washington | Okanogan |
| SDSNH | 20090 | Crotalus | oreganus | oreganus | USA | Washington | Okanogan |
| SDSNH | 20091 | Crotalus | oreganus | oreganus | USA | Washington | Okanogan |
| SDSNH | 20092 | Crotalus | oreganus | oreganus | USA | Washington | Okanogan |
| SDSNH | 20093 | Crotalus | oreganus | oreganus | USA | Washington | Okanogan |
| SDSNH | 20094 | Crotalus | oreganus | oreganus | USA | Washington | Okanogan |
| SDSNH | 20095 | Crotalus | oreganus | oreganus | USA | Washington | Okanogan |
| SDSNH | 20096 | Crotalus | oreganus | oreganus | USA | Washington | Okanogan |
| SDSNH | 20097 | Crotalus | oreganus | oreganus | USA | Washington | Okanogan |
| SDSNH | 20098 | Crotalus | oreganus | oreganus | USA | Washington | Okanogan |
| SDSNH | 20099 | Crotalus | oreganus | oreganus | USA | Washington | Okanogan |
| SDSNH | 20100 | Crotalus | oreganus | oreganus | USA | Washington | Okanogan |
| SDSNH | 20101 | Crotalus | oreganus | oreganus | USA | Washington | Okanogan |
| SDSNH | 20102 | Crotalus | oreganus | oreganus | USA | Washington | Okanogan |
| SDSNH | 20103 | Crotalus | oreganus | oreganus | USA | Washington | Okanogan |
| SDSNH | 20104 | Crotalus | oreganus | oreganus | USA | Washington | Okanogan |
| SDSNH | 20105 | Crotalus | oreganus | oreganus | USA | Washington | Okanogan |
| SDSNH | 20106 | Crotalus | oreganus | oreganus | USA | Washington | Okanogan |
| SDSNH | 20107 | Crotalus | oreganus | oreganus | USA | Washington | Okanogan |
| SDSNH | 20108 | Crotalus | oreganus | oreganus | USA | Washington | Okanogan |
| SDSNH | 20109 | Crotalus | oreganus | oreganus | USA | Washington | Okanogan |
| SDSNH | 20110 | Crotalus | oreganus | oreganus | USA | Washington | Okanogan |
| SDSNH | 20116 | Crotalus | oreganus | helleri | USA | California | Los Angeles |
| SDSNH | 20117 | Crotalus | oreganus | helleri | USA | California | Los Angeles |
| SDSNH | 20197 | Crotalus | oreganus | helleri | Mexico | Baja California Norte |  |
| SDSNH | 20198 | Crotalus | oreganus | helleri | Mexico | Baja California Norte |  |
| SDSNH | 20199 | Crotalus | oreganus | helleri | Mexico | Baja California Norte |  |
| SDSNH | 20403 | Crotalus | oreganus | helleri | USA | California | Riverside |
| SDSNH | 20414 | Crotalus | oreganus | lutosus | USA | Utah | Uintah |
| SDSNH | 20469 | Crotalus | viridis | viridis | USA | Montana | Wheatland |
| SDSNH | 20470 | Crotalus | viridis | viridis | USA | Montana | Wheatland |
| SDSNH | 20471 | Crotalus | viridis | viridis | USA | Montana | Wheatland |
| SDSNH | 20472 | Crotalus | viridis | viridis | USA | Montana | Wheatland |
| SDSNH | 20473 | Crotalus | viridis | viridis | USA | Montana | Wheatland |
| SDSNH | 20474 | Crotalus | viridis | viridis | USA | Montana | Wheatland |
| SDSNH | 20475 | Crotalus | viridis | viridis | USA | Montana | Wheatland |
| SDSNH | 20476 | Crotalus | viridis | viridis | USA | Montana | Wheatland |
| SDSNH | 20477 | Crotalus | viridis | viridis | USA | Montana | Wheatland |
| SDSNH | 20478 | Crotalus | viridis | viridis | USA | Montana | Wheatland |
| SDSNH | 20479 | Crotalus | viridis | viridis | USA | Montana | Wheatland |
| SDSNH | 20480 | Crotalus | viridis | viridis | USA | Montana | Wheatland |
| SDSNH | 20481 | Crotalus | viridis | viridis | USA | Montana | Wheatland |
| SDSNH | 20482 | Crotalus | viridis | viridis | USA | Montana | Wheatland |
| SDSNH | 20483 | Crotalus | viridis | viridis | USA | Montana | Wheatland |
| SDSNH | 20484 | Crotalus | viridis | viridis | USA | Montana | Wheatland |
| SDSNH | 20485 | Crotalus | viridis | viridis | USA | Montana | Wheatland |
| SDSNH | 20486 | Crotalus | viridis | viridis | USA | Montana | Wheatland |
| SDSNH | 20487 | Crotalus | viridis | viridis | USA | Montana | Wheatland |
| SDSNH | 20488 | Crotalus | viridis | viridis | USA | Montana | Wheatland |
| SDSNH | 20489 | Crotalus | viridis | viridis | USA | Montana | Wheatland |
| SDSNH | 20492 | Crotalus | oreganus | helleri | USA | California | San Diego |
| SDSNH | 20594 | Crotalus | oreganus | helleri | USA | California | Los Angeles |
| SDSNH | 20595 | Crotalus | oreganus | helleri | USA | California | Los Angeles |
| SDSNH | 20675 | Crotalus | viridis | viridis | USA | South Dakota | Corson |
| SDSNH | 20676 | Crotalus | viridis | viridis | USA | South Dakota | Corson |
| SDSNH | 20677 | Crotalus | viridis | viridis | USA | South Dakota | Corson |
| SDSNH | 20678 | Crotalus | viridis | viridis | USA | South Dakota | Corson |
| SDSNH | 20679 | Crotalus | viridis | viridis | USA | South Dakota | Corson |
| SDSNH | 20734 | Crotalus | oreganus | helleri | USA | California | Riverside |
| SDSNH | 20735 | Crotalus | oreganus | helleri | USA | California | Riverside |
| SDSNH | 20736 | Crotalus | oreganus | helleri | USA | California | Riverside |
| SDSNH | 20881 | Crotalus | oreganus | helleri | USA | California | San Diego |
| SDSNH | 20960 | Crotalus | oreganus | oreganus | USA | California | Siskiyou |
| SDSNH | 20960 | Crotalus | oreganus | oreganus | USA | California | Siskiyou |
| SDSNH | 20985 | Crotalus | oreganus | helleri | USA | California | San Diego |
| SDSNH | 21133 | Crotalus | oreganus | lutosus | USA | Utah | Washington |
| SDSNH | 21134 | Crotalus | oreganus | helleri | USA | California | San Diego |
| SDSNH | 21142 | Crotalus | oreganus | lutosus | USA | Utah | Tooele |
| SDSNH | 21342 | Crotalus | oreganus | concolor | USA | Colorado | Mesa |
| SDSNH | 21438 | Crotalus | viridis | viridis | USA | Wyoming | Platte |
| SDSNH | 21443 | Crotalus | oreganus | lutosus | USA | Utah | Washington |
| SDSNH | 21444 | Crotalus | oreganus | lutosus | USA | Utah | Washington |
| SDSNH | 21445 | Crotalus | oreganus | lutosus | USA | Utah | Washington |
| SDSNH | 21453 | Crotalus | viridis | viridis | USA | Nebraska | Cherry |
| SDSNH | 21454 | Crotalus | viridis | viridis | USA | Nebraska | Cherry |
| SDSNH | 21455 | Crotalus | viridis | viridis | USA | Nebraska | Cherry |
| SDSNH | 21455 | Crotalus | viridis | viridis | USA | Nebraska | Cherry |
| SDSNH | 21457 | Crotalus | viridis | viridis | USA | Nebraska | Cherry |
| SDSNH | 21458 | Crotalus | viridis | viridis | USA | Nebraska | Cherry |
| SDSNH | 21459 | Crotalus | viridis | viridis | USA | Nebraska | Cherry |
| SDSNH | 21460 | Crotalus | viridis | viridis | USA | Nebraska | Cherry |
| SDSNH | 21461 | Crotalus | viridis | viridis | USA | Nebraska | Cherry |
| SDSNH | 21462 | Crotalus | viridis | viridis | USA | Nebraska | Cherry |
| SDSNH | 21463 | Crotalus | viridis | viridis | USA | Nebraska | Cherry |
| SDSNH | 21464 | Crotalus | viridis | viridis | USA | Nebraska | Cherry |
| SDSNH | 21465 | Crotalus | viridis | viridis | USA | Nebraska | Cherry |
| SDSNH | 21466 | Crotalus | viridis | viridis | USA | Nebraska | Cherry |
| SDSNH | 21467 | Crotalus | viridis | viridis | USA | Nebraska | Cherry |
| SDSNH | 21486 | Crotalus | oreganus | oreganus | USA | California | Mariposa |
| SDSNH | 21487 | Crotalus | oreganus | oreganus | USA | California | Mariposa |
| SDSNH | 21509 | Crotalus | viridis | viridis | USA | Nebraska | Cherry |
| SDSNH | 21510 | Crotalus | viridis | viridis | USA | Nebraska | Cherry |
| SDSNH | 21511 | Crotalus | viridis | viridis | USA | Nebraska | Cherry |
| SDSNH | 21512 | Crotalus | viridis | viridis | USA | Nebraska | Cherry |
| SDSNH | 21700 | Crotalus | viridis | viridis | USA | Nebraska | Cherry |
| SDSNH | 21701 | Crotalus | viridis | viridis | USA | Nebraska | Cherry |
| SDSNH | 21702 | Crotalus | viridis | viridis | USA | Nebraska | Cherry |
| SDSNH | 21703 | Crotalus | viridis | viridis | USA | Nebraska | Cherry |
| SDSNH | 21823 | Crotalus | viridis | viridis | USA | New Mexico | Santa Fe |
| SDSNH | 21824 | Crotalus | oreganus | helleri | USA | California | San Diego |
| SDSNH | 22066 | Crotalus | viridis | viridis | USA | South Dakota | Hughes |
| SDSNH | 22067 | Crotalus | viridis | viridis | USA | South Dakota | Hughes |
| SDSNH | 22068 | Crotalus | viridis | viridis | USA | South Dakota | Hughes |
| SDSNH | 22102 | Crotalus | viridis | viridis | USA | South Dakota | Hughes |
| SDSNH | 22124 | Crotalus | oreganus | helleri | USA | California | San Diego |
| SDSNH | 22241 | Crotalus | oreganus | helleri | Mexico | Baja California Norte |  |
| SDSNH | 22256 | Crotalus | oreganus | helleri | USA | California | San Diego |
| SDSNH | 22271 | Crotalus | viridis | viridis | USA | South Dakota | Hughes |
| SDSNH | 22272 | Crotalus | viridis | viridis | USA | South Dakota | Hughes |
| SDSNH | 22273 | Crotalus | viridis | viridis | USA | South Dakota | Hughes |
| SDSNH | 22376 | Crotalus | oreganus | helleri | USA | California | San Diego |
| SDSNH | 22382 | Crotalus | oreganus | oreganus | USA | California | Sacramento |
| SDSNH | 22436 | Crotalus | oreganus | oreganus | USA | Washington | Okanogan |
| SDSNH | 22459 | Crotalus | oreganus | lutosus | USA | Utah | Kane |
| SDSNH | 22636 | Crotalus | oreganus | helleri | USA | California | San Diego |
| SDSNH | 22638 | Crotalus | oreganus | helleri | USA | California | San Diego |
| SDSNH | 22639 | Crotalus | oreganus | helleri | USA | California | San Diego |
| SDSNH | 22641 | Crotalus | oreganus | oreganus | USA | California | Madera |
| SDSNH | 22642 | Crotalus | oreganus | oreganus | USA | California | Madera |
| SDSNH | 22643 | Crotalus | oreganus | oreganus | USA | California | Madera |
| SDSNH | 22644 | Crotalus | oreganus | oreganus | USA | California | Madera |
| SDSNH | 22645 | Crotalus | oreganus | oreganus | USA | California | Madera |
| SDSNH | 22647 | Crotalus | oreganus | oreganus | USA | California | Madera |
| SDSNH | 22648 | Crotalus | oreganus | oreganus | USA | California | Madera |
| SDSNH | 22649 | Crotalus | oreganus | oreganus | USA | California | Madera |
| SDSNH | 22651 | Crotalus | oreganus | lutosus | USA | Idaho | Bannock |
| SDSNH | 22742 | Crotalus | oreganus | helleri | USA | California | Los Angeles |
| SDSNH | 23007 | Crotalus | oreganus | helleri | USA | California | San Diego |
| SDSNH | 23047 | Crotalus | viridis | nuntius | USA | Arizona | Navajo |
| SDSNH | 23049 | Crotalus | viridis | nuntius | USA | Arizona | Navajo |
| SDSNH | 23049 | Crotalus | viridis | nuntius | USA | Arizona | Navajo |
| SDSNH | 23081 | Crotalus | oreganus | helleri | USA | California | Riverside |
| SDSNH | 23082 | Crotalus | oreganus | helleri | USA | California | Orange |
| SDSNH | 23083 | Crotalus | oreganus | helleri | USA | California | Orange |
| SDSNH | 23084 | Crotalus | oreganus | helleri | USA | California | Orange |
| SDSNH | 23085 | Crotalus | oreganus | helleri | USA | California | Orange |
| SDSNH | 23098 | Crotalus | oreganus | helleri | USA | California | San Diego |
| SDSNH | 23124 | Crotalus | oreganus | oreganus | USA | California | Sacramento |
| SDSNH | 23157 | Crotalus | oreganus | lutosus | USA | California | Modoc |
| SDSNH | 23165 | Crotalus | oreganus | helleri | USA | California | San Diego |
| SDSNH | 23201 | Crotalus | oreganus | helleri | USA | California | San Diego |
| SDSNH | 23236 | Crotalus | oreganus | helleri | USA | California | San Diego |
| SDSNH | 23435 | Crotalus | oreganus | helleri | USA | California | Los Angeles |
| SDSNH | 23658 | Crotalus | oreganus | helleri | Mexico | Baja California Norte |  |
| SDSNH | 23660 | Crotalus | viridis | viridis | USA | South Dakota | Pennington |
| SDSNH | 23661 | Crotalus | viridis | viridis | USA | South Dakota | Pennington |
| SDSNH | 23790 | Crotalus | oreganus | lutosus | USA | Utah | Washington |
| SDSNH | 23894 | Crotalus | oreganus | lutosus | USA | Idaho | Lewis |
| SDSNH | 23965 | Crotalus | oreganus | oreganus | USA | California | Kern |
| SDSNH | 23989 | Crotalus | oreganus | oreganus | USA | California | Kern |
| SDSNH | 23990 | Crotalus | oreganus | oreganus | USA | California | Kern |
| SDSNH | 24038 | Crotalus | viridis | viridis | USA | South Dakota | Meade |
| SDSNH | 24039 | Crotalus | viridis | viridis | USA | South Dakota | Meade |
| SDSNH | 24040 | Crotalus | viridis | viridis | USA | South Dakota | Meade |
| SDSNH | 24041 | Crotalus | viridis | viridis | USA | South Dakota | Meade |
| SDSNH | 24042 | Crotalus | viridis | viridis | USA | South Dakota | Meade |
| SDSNH | 24044 | Crotalus | oreganus | helleri | USA | California | Riverside |
| SDSNH | 24062 | Crotalus | viridis | viridis | USA | Utah | Washington |
| SDSNH | 24078 | Crotalus | oreganus | lutosus | USA | Utah | Washington |
| SDSNH | 24219 | Crotalus | oreganus | lutosus | USA | Utah | Washington |
| SDSNH | 24276 | Crotalus | oreganus | helleri | USA | California | Orange |
| SDSNH | 24286 | Crotalus | oreganus | oreganus | USA | Washington | Okanogan |
| SDSNH | 24287 | Crotalus | oreganus | oreganus | USA | Washington | Okanogan |
| SDSNH | 24289 | Crotalus | oreganus | oreganus | USA | California | Mariposa |
| SDSNH | 24290 | Crotalus | oreganus | oreganus | USA | California | Mariposa |
| SDSNH | 24377 | Crotalus | viridis | viridis | USA | South Dakota | Pennington |
| SDSNH | 24669 | Crotalus | oreganus | lutosus | USA | Utah | Millard |
| SDSNH | 24670 | Crotalus | oreganus | lutosus | USA | Utah | Washington |
| SDSNH | 24671 | Crotalus | oreganus | lutosus | USA | Utah | Kane |
| SDSNH | 24672 | Crotalus | oreganus | lutosus | USA | Utah | Kane |
| SDSNH | 24679 | Crotalus | oreganus | lutosus | USA | Utah | Kane |
| SDSNH | 25257 | Crotalus | oreganus | lutosus | USA | Utah | Washington |
| SDSNH | 25258 | Crotalus | oreganus | lutosus | USA | Utah | Washington |
| SDSNH | 25287 | Crotalus | oreganus | lutosus | USA | Utah | Washington |
| SDSNH | 25318 | Crotalus | oreganus | helleri | USA | California | Los Angeles |
| SDSNH | 25319 | Crotalus | oreganus | helleri | USA | California | San Diego |
| SDSNH | 25515 | Crotalus | oreganus | lutosus | USA | Utah | Washington |
| SDSNH | 25542 | Crotalus | oreganus | oreganus | USA | California | San Luis Obispo |
| SDSNH | 25698 | Crotalus | viridis | nuntius | USA | Arizona | Coconino |
| SDSNH | 25794 | Crotalus | oreganus | abyssus | USA | Arizona | Coconino |
| SDSNH | 25795 | Crotalus | oreganus | abyssus | USA | Arizona | Coconino |
| SDSNH | 25838 | Crotalus | oreganus | lutosus | USA | Idaho | Bannock |
| SDSNH | 25839 | Crotalus | oreganus | lutosus | USA | Idaho | Bannock |
| SDSNH | 25840 | Crotalus | oreganus | lutosus | USA | Idaho | Bannock |
| SDSNH | 25841 | Crotalus | oreganus | lutosus | USA | Idaho | Bannock |
| SDSNH | 25842 | Crotalus | oreganus | lutosus | USA | Idaho | Bannock |
| SDSNH | 25843 | Crotalus | oreganus | lutosus | USA | Idaho | Bannock |
| SDSNH | 25844 | Crotalus | oreganus | lutosus | USA | Idaho | Bannock |
| SDSNH | 25845 | Crotalus | oreganus | lutosus | USA | Idaho | Bannock |
| SDSNH | 25946 | Crotalus | oreganus | lutosus | USA | Idaho | Bannock |
| SDSNH | 26054 | Crotalus | oreganus | lutosus | USA | Utah | Box Elder |
| SDSNH | 26065 | Crotalus | oreganus | lutosus | USA | Utah | Box Elder |
| SDSNH | 26078 | Crotalus | oreganus | lutosus | USA | Utah | Uintah |
| SDSNH | 26079 | Crotalus | oreganus | lutosus | USA | Utah | Uintah |
| SDSNH | 26446 | Crotalus | oreganus | helleri | USA | California | San Bernardino |
| SDSNH | 26518 | Crotalus | oreganus | oreganus | USA | California | Placer |
| SDSNH | 26714 | Crotalus | oreganus | helleri | USA | California | San Diego |
| SDSNH | 26718 | Crotalus | viridis | viridis | USA | Montana | Wheatland |
| SDSNH | 26734 | Crotalus | oreganus | lutosus | USA | Utah | Uintah |
| SDSNH | 26757 | Crotalus | oreganus | oreganus | USA | California | Monterey |
| SDSNH | 26763 | Crotalus | oreganus | helleri | USA | California | San Diego |
| SDSNH | 26768 | Crotalus | oreganus | helleri | USA | California | San Diego |
| SDSNH | 26769 | Crotalus | viridis | viridis | USA | Iowa | Plymouth |
| SDSNH | 26770 | Crotalus | viridis | viridis | USA | Iowa | Plymouth |
| SDSNH | 26776 | Crotalus | oreganus | helleri | USA | California | San Diego |
| SDSNH | 26843 | Crotalus | oreganus | helleri | USA | California | San Diego |
| SDSNH | 26931 | Crotalus | oreganus | helleri | USA | California | San Diego |
| SDSNH | 26932 | Crotalus | oreganus | helleri | USA | California | San Diego |
| SDSNH | 27148 | Crotalus | oreganus | oreganus | Canada | British Columbia |  |
| SDSNH | 27247 | Crotalus | oreganus | helleri | USA | California | Los Angeles |
| SDSNH | 27262 | Crotalus | oreganus | helleri | USA | California | San Diego |
| SDSNH | 27425 | Crotalus | oreganus | cerberus | USA | Arizona | Coconino |
| SDSNH | 27535 | Crotalus | oreganus | oreganus | USA | California | Monterey |
| SDSNH | 27555 | Crotalus | oreganus | helleri | USA | California | San Diego |
| SDSNH | 27722 | Crotalus | oreganus | helleri | USA | California | Los Angeles |
| SDSNH | 27723 | Crotalus | oreganus | helleri | USA | California | San Diego |
| SDSNH | 27993 | Crotalus | oreganus | lutosus | USA | California | Mono |
| SDSNH | 27994 | Crotalus | oreganus | lutosus | USA | California | Mono |
| SDSNH | 27995 | Crotalus | oreganus | lutosus | USA | Nevada | Eureka |
| SDSNH | 27997 | Crotalus | oreganus | oreganus | USA | California | Mendocino |
| SDSNH | 27998 | Crotalus | oreganus | oreganus | USA | California | Kern |
| SDSNH | 28056 | Crotalus | oreganus | oreganus | USA | California | Humboldt |
| SDSNH | 28058 | Crotalus | oreganus | oreganus | USA | California | Humboldt |
| SDSNH | 28059 | Crotalus | oreganus | oreganus | USA | California | Humboldt |
| SDSNH | 28060 | Crotalus | oreganus | lutosus | USA | Utah | Uintah |
| SDSNH | 28061 | Crotalus | oreganus | lutosus | USA | Utah | Uintah |
| SDSNH | 28061 | Crotalus | oreganus | lutosus | USA | Utah | Uintah |
| SDSNH | 28063 | Crotalus | oreganus | helleri | USA | California | San Diego |
| SDSNH | 28078 | Crotalus | oreganus | helleri | USA | California | San Diego |
| SDSNH | 28079 | Crotalus | oreganus | helleri | USA | California | San Diego |
| SDSNH | 28093 | Crotalus | oreganus | helleri | USA | California | San Diego |
| SDSNH | 28115 | Crotalus | viridis | viridis | USA | South Dakota | Dewey |
| SDSNH | 28116 | Crotalus | viridis | viridis | USA | South Dakota | Dewey |
| SDSNH | 28117 | Crotalus | viridis | viridis | USA | South Dakota | Dewey |
| SDSNH | 28118 | Crotalus | viridis | viridis | USA | South Dakota | Dewey |
| SDSNH | 28119 | Crotalus | viridis | viridis | USA | South Dakota | Dewey |
| SDSNH | 28120 | Crotalus | viridis | viridis | USA | South Dakota | Dewey |
| SDSNH | 28121 | Crotalus | viridis | viridis | USA | South Dakota | Dewey |
| SDSNH | 28122 | Crotalus | viridis | viridis | USA | South Dakota | Dewey |
| SDSNH | 28123 | Crotalus | viridis | viridis | USA | South Dakota | Dewey |
| SDSNH | 28124 | Crotalus | viridis | viridis | USA | South Dakota | Dewey |
| SDSNH | 28125 | Crotalus | viridis | viridis | USA | South Dakota | Dewey |
| SDSNH | 28126 | Crotalus | viridis | viridis | USA | South Dakota | Dewey |
| SDSNH | 28127 | Crotalus | viridis | viridis | USA | South Dakota | Dewey |
| SDSNH | 28128 | Crotalus | viridis | viridis | USA | South Dakota | Dewey |
| SDSNH | 28129 | Crotalus | viridis | viridis | USA | South Dakota | Dewey |
| SDSNH | 28130 | Crotalus | viridis | viridis | USA | South Dakota | Dewey |
| SDSNH | 28131 | Crotalus | viridis | viridis | USA | South Dakota | Dewey |
| SDSNH | 28132 | Crotalus | viridis | viridis | USA | South Dakota | Dewey |
| SDSNH | 28133 | Crotalus | viridis | viridis | USA | South Dakota | Dewey |
| SDSNH | 28134 | Crotalus | viridis | viridis | USA | South Dakota | Dewey |
| SDSNH | 28135 | Crotalus | viridis | viridis | USA | South Dakota | Dewey |
| SDSNH | 28136 | Crotalus | viridis | viridis | USA | South Dakota | Dewey |
| SDSNH | 28137 | Crotalus | viridis | viridis | USA | South Dakota | Dewey |
| SDSNH | 28138 | Crotalus | viridis | viridis | USA | South Dakota | Dewey |
| SDSNH | 28139 | Crotalus | viridis | viridis | USA | South Dakota | Dewey |
| SDSNH | 28140 | Crotalus | viridis | viridis | USA | South Dakota | Dewey |
| SDSNH | 28141 | Crotalus | viridis | viridis | USA | South Dakota | Dewey |
| SDSNH | 28142 | Crotalus | viridis | viridis | USA | South Dakota | Dewey |
| SDSNH | 28143 | Crotalus | viridis | viridis | USA | South Dakota | Dewey |
| SDSNH | 28144 | Crotalus | viridis | viridis | USA | South Dakota | Dewey |
| SDSNH | 28145 | Crotalus | viridis | viridis | USA | South Dakota | Dewey |
| SDSNH | 28146 | Crotalus | viridis | viridis | USA | South Dakota | Dewey |
| SDSNH | 28147 | Crotalus | viridis | viridis | USA | South Dakota | Dewey |
| SDSNH | 28148 | Crotalus | viridis | viridis | USA | South Dakota | Dewey |
| SDSNH | 28149 | Crotalus | viridis | viridis | USA | South Dakota | Dewey |
| SDSNH | 28150 | Crotalus | viridis | viridis | USA | South Dakota | Dewey |
| SDSNH | 28151 | Crotalus | viridis | viridis | USA | South Dakota | Dewey |
| SDSNH | 28152 | Crotalus | viridis | viridis | USA | South Dakota | Dewey |
| SDSNH | 28153 | Crotalus | viridis | viridis | USA | South Dakota | Dewey |
| SDSNH | 28154 | Crotalus | viridis | viridis | USA | South Dakota | Dewey |
| SDSNH | 28155 | Crotalus | viridis | viridis | USA | South Dakota | Dewey |
| SDSNH | 28156 | Crotalus | viridis | viridis | USA | South Dakota | Dewey |
| SDSNH | 28157 | Crotalus | viridis | viridis | USA | South Dakota | Dewey |
| SDSNH | 28158 | Crotalus | viridis | viridis | USA | South Dakota | Dewey |
| SDSNH | 28159 | Crotalus | viridis | viridis | USA | South Dakota | Dewey |
| SDSNH | 28160 | Crotalus | viridis | viridis | USA | South Dakota | Dewey |
| SDSNH | 28161 | Crotalus | viridis | viridis | USA | South Dakota | Dewey |
| SDSNH | 28162 | Crotalus | viridis | viridis | USA | South Dakota | Dewey |
| SDSNH | 28163 | Crotalus | viridis | viridis | USA | South Dakota | Dewey |
| SDSNH | 28164 | Crotalus | viridis | viridis | USA | South Dakota | Dewey |
| SDSNH | 28165 | Crotalus | viridis | viridis | USA | South Dakota | Dewey |
| SDSNH | 28166 | Crotalus | viridis | viridis | USA | South Dakota | Dewey |
| SDSNH | 28167 | Crotalus | viridis | viridis | USA | South Dakota | Dewey |
| SDSNH | 28168 | Crotalus | viridis | viridis | USA | South Dakota | Dewey |
| SDSNH | 28169 | Crotalus | viridis | viridis | USA | South Dakota | Dewey |
| SDSNH | 28224 | Crotalus | oreganus | oreganus | USA | California | Alpine |
| SDSNH | 28321 | Crotalus | oreganus | helleri | USA | California | Los Angeles |
| SDSNH | 28324 | Crotalus | oreganus | helleri | USA | California | San Diego |
| SDSNH | 28384 | Crotalus | viridis | viridis | USA | South Dakota | Pennington |
| SDSNH | 28461 | Crotalus | oreganus | oreganus | USA | California | San Luis Obispo |
| SDSNH | 28480 | Crotalus | oreganus | helleri | USA | California | San Diego |
| SDSNH | 28684 | Crotalus | oreganus | helleri | USA | California | San Diego |
| SDSNH | 28685 | Crotalus | oreganus | helleri | USA | California | San Diego |
| SDSNH | 28731 | Crotalus | oreganus | lutosus | USA | Idaho | Ada |
| SDSNH | 28732 | Crotalus | oreganus | lutosus | USA | Idaho | Ada |
| SDSNH | 28733 | Crotalus | oreganus | lutosus | USA | Idaho | Ada |
| SDSNH | 28809 | Crotalus | oreganus | cerberus | USA | Arizona | Coconino |
| SDSNH | 29100 | Crotalus | oreganus | lutosus | USA | Idaho | Ada |
| SDSNH | 29101 | Crotalus | oreganus | lutosus | USA | Idaho | Ada |
| SDSNH | 29102 | Crotalus | oreganus | lutosus | USA | Idaho | Ada |
| SDSNH | 29103 | Crotalus | oreganus | lutosus | USA | Idaho | Ada |
| SDSNH | 29104 | Crotalus | oreganus | lutosus | USA | Idaho | Ada |
| SDSNH | 29105 | Crotalus | oreganus | lutosus | USA | Idaho | Ada |
| SDSNH | 29106 | Crotalus | oreganus | lutosus | USA | Idaho | Ada |
| SDSNH | 29107 | Crotalus | oreganus | lutosus | USA | Idaho | Ada |
| SDSNH | 29108 | Crotalus | oreganus | lutosus | USA | Idaho | Ada |
| SDSNH | 29109 | Crotalus | oreganus | lutosus | USA | Idaho | Ada |
| SDSNH | 29205 | Crotalus | oreganus | oreganus | USA | Washington | Okanogan |
| SDSNH | 29269 | Crotalus | oreganus | oreganus | USA | California | Lake |
| SDSNH | 29470 | Crotalus | oreganus | lutosus | USA | Idaho | Ada |
| SDSNH | 29471 | Crotalus | oreganus | lutosus | USA | Idaho | Ada |
| SDSNH | 29472 | Crotalus | oreganus | lutosus | USA | Idaho | Ada |
| SDSNH | 29473 | Crotalus | oreganus | lutosus | USA | Idaho | Ada |
| SDSNH | 29474 | Crotalus | oreganus | lutosus | USA | Idaho | Ada |
| SDSNH | 29511 | Crotalus | oreganus | helleri | USA | California | San Diego |
| SDSNH | 29512 | Crotalus | oreganus | helleri | USA | California | San Diego |
| SDSNH | 29593 | Crotalus | oreganus | oreganus | USA | Washington | Okanogan |
| SDSNH | 29593 | Crotalus | oreganus | oreganus | USA | Washington | Okanogan |
| SDSNH | 29594 | Crotalus | oreganus | oreganus | USA | Washington | Okanogan |
| SDSNH | 29595 | Crotalus | oreganus | oreganus | USA | Washington | Okanogan |
| SDSNH | 29596 | Crotalus | oreganus | oreganus | USA | Washington | Okanogan |
| SDSNH | 29597 | Crotalus | oreganus | oreganus | USA | Washington | Okanogan |
| SDSNH | 29599 | Crotalus | oreganus | oreganus | USA | Washington | Okanogan |
| SDSNH | 29600 | Crotalus | oreganus | oreganus | USA | Washington | Okanogan |
| SDSNH | 29601 | Crotalus | oreganus | oreganus | USA | Washington | Okanogan |
| SDSNH | 29602 | Crotalus | oreganus | oreganus | USA | Washington | Okanogan |
| SDSNH | 29603 | Crotalus | oreganus | oreganus | USA | Washington | Okanogan |
| SDSNH | 29605 | Crotalus | oreganus | oreganus | USA | Washington | Okanogan |
| SDSNH | 29606 | Crotalus | oreganus | oreganus | USA | Washington | Okanogan |
| SDSNH | 29607 | Crotalus | oreganus | oreganus | USA | Washington | Okanogan |
| SDSNH | 29608 | Crotalus | oreganus | oreganus | USA | Washington | Okanogan |
| SDSNH | 29609 | Crotalus | oreganus | oreganus | USA | Washington | Okanogan |
| SDSNH | 29610 | Crotalus | oreganus | oreganus | USA | Washington | Okanogan |
| SDSNH | 29634 | Crotalus | oreganus | oreganus | USA | California |  |
| SDSNH | 29635 | Crotalus | oreganus | oreganus | USA | California |  |
| SDSNH | 29766 | Crotalus | oreganus | oreganus | USA | Washington | Okanogan |
| SDSNH | 29767 | Crotalus | oreganus | oreganus | USA | Washington | Okanogan |
| SDSNH | 29768 | Crotalus | oreganus | oreganus | USA | Washington | Okanogan |
| SDSNH | 29769 | Crotalus | oreganus | oreganus | USA | Washington | Okanogan |
| SDSNH | 29770 | Crotalus | oreganus | oreganus | USA | Washington | Okanogan |
| SDSNH | 29771 | Crotalus | oreganus | oreganus | USA | Washington | Okanogan |
| SDSNH | 29772 | Crotalus | oreganus | oreganus | USA | Washington | Okanogan |
| SDSNH | 29773 | Crotalus | oreganus | oreganus | USA | Washington | Okanogan |
| SDSNH | 29774 | Crotalus | oreganus | oreganus | USA | Washington | Okanogan |
| SDSNH | 29775 | Crotalus | oreganus | oreganus | USA | Washington | Okanogan |
| SDSNH | 29776 | Crotalus | oreganus | oreganus | USA | Washington | Okanogan |
| SDSNH | 29777 | Crotalus | oreganus | oreganus | USA | Washington | Okanogan |
| SDSNH | 29779 | Crotalus | oreganus | oreganus | USA | Washington | Okanogan |
| SDSNH | 29780 | Crotalus | oreganus | oreganus | USA | Washington | Okanogan |
| SDSNH | 29781 | Crotalus | oreganus | oreganus | USA | Washington | Okanogan |
| SDSNH | 29782 | Crotalus | oreganus | oreganus | USA | Washington | Okanogan |
| SDSNH | 29783 | Crotalus | oreganus | oreganus | USA | Washington | Okanogan |
| SDSNH | 29784 | Crotalus | oreganus | oreganus | USA | Washington | Okanogan |
| SDSNH | 29785 | Crotalus | oreganus | oreganus | USA | Washington | Okanogan |
| SDSNH | 29786 | Crotalus | oreganus | oreganus | USA | Washington | Okanogan |
| SDSNH | 29787 | Crotalus | oreganus | oreganus | USA | Washington | Okanogan |
| SDSNH | 29788 | Crotalus | oreganus | oreganus | USA | Washington | Okanogan |
| SDSNH | 29789 | Crotalus | oreganus | oreganus | USA | Washington | Okanogan |
| SDSNH | 29790 | Crotalus | oreganus | oreganus | USA | Washington | Okanogan |
| SDSNH | 29791 | Crotalus | oreganus | oreganus | USA | Washington | Okanogan |
| SDSNH | 29816 | Crotalus | oreganus | helleri | USA | California | Riverside |
| SDSNH | 29819 | Crotalus | oreganus | lutosus | USA | Utah | Weber |
| SDSNH | 29820 | Crotalus | oreganus | lutosus | USA | Utah | Weber |
| SDSNH | 29833 | Crotalus | oreganus | oreganus | USA | Washington | Okanogan |
| SDSNH | 29834 | Crotalus | oreganus | oreganus | USA | Washington | Okanogan |
| SDSNH | 29835 | Crotalus | oreganus | oreganus | USA | Washington | Okanogan |
| SDSNH | 29836 | Crotalus | oreganus | oreganus | USA | Washington | Okanogan |
| SDSNH | 29837 | Crotalus | oreganus | oreganus | USA | Washington | Okanogan |
| SDSNH | 29838 | Crotalus | oreganus | oreganus | USA | Washington | Okanogan |
| SDSNH | 29840 | Crotalus | oreganus | oreganus | USA | Washington | Okanogan |
| SDSNH | 29841 | Crotalus | oreganus | oreganus | USA | Washington | Okanogan |
| SDSNH | 29842 | Crotalus | oreganus | oreganus | USA | Washington | Okanogan |
| SDSNH | 29843 | Crotalus | oreganus | oreganus | USA | Washington | Okanogan |
| SDSNH | 29844 | Crotalus | oreganus | oreganus | USA | Washington | Okanogan |
| SDSNH | 29845 | Crotalus | oreganus | oreganus | USA | Washington | Okanogan |
| SDSNH | 29846 | Crotalus | oreganus | oreganus | USA | Washington | Okanogan |
| SDSNH | 29847 | Crotalus | oreganus | oreganus | USA | Washington | Okanogan |
| SDSNH | 29848 | Crotalus | oreganus | oreganus | USA | Washington | Okanogan |
| SDSNH | 29849 | Crotalus | oreganus | oreganus | USA | Washington | Okanogan |
| SDSNH | 29850 | Crotalus | oreganus | oreganus | USA | Washington | Okanogan |
| SDSNH | 29851 | Crotalus | oreganus | oreganus | USA | Washington | Okanogan |
| SDSNH | 29852 | Crotalus | oreganus | oreganus | USA | Washington | Okanogan |
| SDSNH | 29853 | Crotalus | oreganus | oreganus | USA | Washington | Okanogan |
| SDSNH | 29854 | Crotalus | oreganus | oreganus | USA | Washington | Okanogan |
| SDSNH | 29856 | Crotalus | oreganus | oreganus | USA | Washington | Okanogan |
| SDSNH | 29856 | Crotalus | oreganus | oreganus | USA | Washington | Okanogan |
| SDSNH | 29857 | Crotalus | oreganus | oreganus | USA | Washington | Okanogan |
| SDSNH | 29858 | Crotalus | oreganus | oreganus | USA | Washington | Okanogan |
| SDSNH | 29859 | Crotalus | oreganus | oreganus | USA | Washington | Okanogan |
| SDSNH | 29860 | Crotalus | oreganus | oreganus | USA | Washington | Okanogan |
| SDSNH | 29861 | Crotalus | oreganus | oreganus | USA | Washington | Okanogan |
| SDSNH | 29862 | Crotalus | oreganus | oreganus | USA | Washington | Okanogan |
| SDSNH | 29863 | Crotalus | oreganus | oreganus | USA | Washington | Okanogan |
| SDSNH | 29864 | Crotalus | oreganus | oreganus | USA | Washington | Okanogan |
| SDSNH | 29950 | Crotalus | oreganus | oreganus | USA | Washington | Okanogan |
| SDSNH | 29951 | Crotalus | oreganus | oreganus | USA | Washington | Okanogan |
| SDSNH | 29952 | Crotalus | oreganus | oreganus | USA | Washington | Okanogan |
| SDSNH | 29953 | Crotalus | oreganus | oreganus | USA | Washington | Okanogan |
| SDSNH | 29953 | Crotalus | oreganus | oreganus | USA | Washington | Okanogan |
| SDSNH | 29954 | Crotalus | oreganus | oreganus | USA | Washington | Okanogan |
| SDSNH | 29955 | Crotalus | oreganus | oreganus | USA | Washington | Okanogan |
| SDSNH | 29956 | Crotalus | oreganus | oreganus | USA | Washington | Okanogan |
| SDSNH | 29957 | Crotalus | oreganus | oreganus | USA | Washington | Okanogan |
| SDSNH | 29959 | Crotalus | oreganus | oreganus | USA | Washington | Okanogan |
| SDSNH | 29960 | Crotalus | oreganus | oreganus | USA | Washington | Okanogan |
| SDSNH | 29961 | Crotalus | oreganus | oreganus | USA | Washington | Okanogan |
| SDSNH | 29962 | Crotalus | oreganus | oreganus | USA | Washington | Okanogan |
| SDSNH | 29963 | Crotalus | oreganus | oreganus | USA | Washington | Okanogan |
| SDSNH | 29964 | Crotalus | oreganus | oreganus | USA | Washington | Okanogan |
| SDSNH | 29965 | Crotalus | oreganus | oreganus | USA | Washington | Okanogan |
| SDSNH | 29966 | Crotalus | oreganus | oreganus | USA | Washington | Okanogan |
| SDSNH | 29967 | Crotalus | oreganus | oreganus | USA | Washington | Okanogan |
| SDSNH | 30441 | Crotalus | oreganus | helleri | USA | California | San Diego |
| SDSNH | 30469 | Crotalus | oreganus | helleri | USA | California | Riverside |
| SDSNH | 30475 | Crotalus | oreganus | helleri | USA | California | Riverside |
| SDSNH | 30514 | Crotalus | viridis | viridis | USA | Idaho | Lemhi |
| SDSNH | 30515 | Crotalus | viridis | viridis | USA | Idaho | Lemhi |
| SDSNH | 30516 | Crotalus | oreganus | oreganus | USA | California | Lassen |
| SDSNH | 30524 | Crotalus | oreganus | oreganus | USA | California | Plumas |
| SDSNH | 30728 | Crotalus | viridis | viridis | USA | South Dakota | Pennington |
| SDSNH | 30729 | Crotalus | viridis | viridis | USA | South Dakota | Pennington |
| SDSNH | 30730 | Crotalus | viridis | viridis | USA | South Dakota | Pennington |
| SDSNH | 30731 | Crotalus | viridis | viridis | USA | South Dakota | Pennington |
| SDSNH | 30732 | Crotalus | viridis | viridis | USA | South Dakota | Pennington |
| SDSNH | 30733 | Crotalus | viridis | viridis | USA | South Dakota | Pennington |
| SDSNH | 30734 | Crotalus | viridis | viridis | USA | South Dakota | Pennington |
| SDSNH | 31015 | Crotalus | viridis | viridis | USA | South Dakota | Mellette |
| SDSNH | 31016 | Crotalus | viridis | viridis | USA | South Dakota | Mellette |
| SDSNH | 31222 | Crotalus | oreganus | helleri | USA | California | San Diego |
| SDSNH | 31258 | Crotalus | oreganus | helleri | USA | California | San Diego |
| SDSNH | 31435 | Crotalus | oreganus | helleri | Mexico | Baja California Norte |  |
| SDSNH | 31437 | Crotalus | oreganus | helleri | Mexico | Baja California Norte |  |
| SDSNH | 31439 | Crotalus | oreganus | helleri | Mexico | Baja California Norte |  |
| SDSNH | 31462 | Crotalus | oreganus | oreganus | USA | California | Fresno |
| SDSNH | 31463 | Crotalus | oreganus | oreganus | USA | California | Fresno |
| SDSNH | 31484 | Crotalus | oreganus | oreganus | USA | California | Fresno |
| SDSNH | 31488 | Crotalus | oreganus | helleri | USA | California | San Diego |
| SDSNH | 31529 | Crotalus | oreganus | oreganus | USA | California | Kern |
| SDSNH | 31530 | Crotalus | oreganus | oreganus | USA | California | Kings |
| SDSNH | 31541 | Crotalus | oreganus | helleri | USA | California | San Diego |
| SDSNH | 31545 | Crotalus | oreganus | oreganus | USA | California | San Luis Obispo |
| SDSNH | 31761 | Crotalus | oreganus | lutosus | USA | Utah | Weber |
| SDSNH | 31805 | Crotalus | oreganus | lutosus | USA | Utah | Washington |
| SDSNH | 31807 | Crotalus | oreganus | helleri | USA | California | San Diego |
| SDSNH | 31809 | Crotalus | oreganus | oreganus | USA | California | Fresno |
| SDSNH | 31810 | Crotalus | oreganus | oreganus | USA | California | Fresno |
| SDSNH | 31814 | Crotalus | viridis | viridis | USA | Montana | Beaverhead |
| SDSNH | 31815 | Crotalus | viridis | viridis | USA | Montana | Beaverhead |
| SDSNH | 31827 | Crotalus | viridis | viridis | USA | Montana | Beaverhead |
| SDSNH | 31828 | Crotalus | viridis | viridis | USA | Montana | Beaverhead |
| SDSNH | 31829 | Crotalus | viridis | viridis | USA | Montana | Beaverhead |
| SDSNH | 31830 | Crotalus | viridis | viridis | USA | Montana | Beaverhead |
| SDSNH | 31831 | Crotalus | viridis | viridis | USA | Montana | Beaverhead |
| SDSNH | 31832 | Crotalus | viridis | viridis | USA | Montana | Beaverhead |
| SDSNH | 31950 | Crotalus | oreganus | helleri | USA | California | San Diego |
| SDSNH | 31962 | Crotalus | oreganus | helleri | USA | California | San Diego |
| SDSNH | 32069 | Crotalus | viridis | viridis | USA | South Dakota | Jackson |
| SDSNH | 32134 | Crotalus | oreganus | helleri | USA | California | Santa Barbara |
| SDSNH | 32282 | Crotalus | oreganus | helleri | USA | California | San Diego |
| SDSNH | 32283 | Crotalus | oreganus | helleri | USA | California | Los Angeles |
| SDSNH | 32522 | Crotalus | viridis | viridis | USA | Idaho | Lemhi |
| SDSNH | 32547 | Crotalus | viridis | viridis | USA | South Dakota | Jones |
| SDSNH | 32553 | Crotalus | viridis | viridis | USA | Idaho | Lemhi |
| SDSNH | 32554 | Crotalus | viridis | viridis | USA | Idaho | Lemhi |
| SDSNH | 32555 | Crotalus | viridis | viridis | USA | Idaho | Lemhi |
| SDSNH | 32567 | Crotalus | viridis | viridis | USA | South Dakota | Jones |
| SDSNH | 32568 | Crotalus | viridis | viridis | USA | South Dakota | Jones |
| SDSNH | 32569 | Crotalus | viridis | viridis | USA | South Dakota | Jones |
| SDSNH | 32570 | Crotalus | viridis | viridis | USA | South Dakota | Jones |
| SDSNH | 32571 | Crotalus | viridis | viridis | USA | South Dakota | Jones |
| SDSNH | 32572 | Crotalus | viridis | viridis | USA | South Dakota | Jones |
| SDSNH | 32573 | Crotalus | viridis | viridis | USA | South Dakota | Jones |
| SDSNH | 32575 | Crotalus | viridis | viridis | USA | South Dakota | Jones |
| SDSNH | 32576 | Crotalus | viridis | viridis | USA | South Dakota | Jones |
| SDSNH | 32577 | Crotalus | viridis | viridis | USA | South Dakota | Jones |
| SDSNH | 32578 | Crotalus | viridis | viridis | USA | South Dakota | Jones |
| SDSNH | 32579 | Crotalus | viridis | viridis | USA | South Dakota | Jones |
| SDSNH | 32580 | Crotalus | viridis | viridis | USA | South Dakota | Jones |
| SDSNH | 32581 | Crotalus | viridis | viridis | USA | South Dakota | Jones |
| SDSNH | 32581 | Crotalus | viridis | viridis | USA | South Dakota | Jones |
| SDSNH | 32582 | Crotalus | viridis | viridis | USA | South Dakota | Jones |
| SDSNH | 32583 | Crotalus | viridis | viridis | USA | South Dakota | Jones |
| SDSNH | 32584 | Crotalus | viridis | viridis | USA | South Dakota | Jones |
| SDSNH | 32585 | Crotalus | viridis | viridis | USA | South Dakota | Jones |
| SDSNH | 32586 | Crotalus | viridis | viridis | USA | South Dakota | Jones |
| SDSNH | 32588 | Crotalus | viridis | viridis | USA | South Dakota | Jones |
| SDSNH | 32608 | Crotalus | viridis | viridis | USA | South Dakota | Jackson |
| SDSNH | 32610 | Crotalus | viridis | viridis | USA | South Dakota | Jackson |
| SDSNH | 32611 | Crotalus | viridis | viridis | USA | South Dakota | Jackson |
| SDSNH | 32612 | Crotalus | viridis | viridis | USA | South Dakota | Jackson |
| SDSNH | 32613 | Crotalus | viridis | viridis | USA | South Dakota | Jackson |
| SDSNH | 32614 | Crotalus | viridis | viridis | USA | South Dakota | Jackson |
| SDSNH | 32615 | Crotalus | viridis | viridis | USA | South Dakota | Jackson |
| SDSNH | 32616 | Crotalus | viridis | viridis | USA | South Dakota | Jackson |
| SDSNH | 32655 | Crotalus | oreganus | lutosus | USA | Idaho | Lemhi |
| SDSNH | 32670 | Crotalus | oreganus | helleri | USA | California | Los Angeles |
| SDSNH | 32749 | Crotalus | oreganus | helleri | USA | California | San Diego |
| SDSNH | 32750 | Crotalus | oreganus | oreganus | USA | California | Kern |
| SDSNH | 32775 | Crotalus | oreganus | helleri | USA | California | San Diego |
| SDSNH | 32874 | Crotalus | oreganus | helleri | USA | California | Los Angeles |
| SDSNH | 32959 | Crotalus | oreganus | helleri | USA | California | San Diego |
| SDSNH | 33045 | Crotalus | oreganus | abyssus | USA | Arizona | Mohave |
| SDSNH | 33046 | Crotalus | oreganus | abyssus | USA | Arizona | Mohave |
| SDSNH | 33047 | Crotalus | oreganus | oreganus | USA | Oregon | Wallowa |
| SDSNH | 33221 | Crotalus | oreganus | helleri | USA | California | San Diego |
| SDSNH | 33405 | Crotalus | oreganus | helleri | USA | California | Santa Barbara |
| SDSNH | 33578 | Crotalus | oreganus | helleri | USA | California | San Diego |
| SDSNH | 33579 | Crotalus | oreganus | helleri | USA | California | San Diego |
| SDSNH | 33779 | Crotalus | oreganus | helleri | USA | California | San Diego |
| SDSNH | 33809 | Crotalus | oreganus | helleri | USA | California | San Diego |
| SDSNH | 33929 | Crotalus | oreganus | oreganus | USA | California | Fresno |
| SDSNH | 33930 | Crotalus | oreganus | helleri | USA | California | San Diego |
| SDSNH | 33931 | Crotalus | oreganus | lutosus | USA | Utah | Box Elder |
| SDSNH | 33932 | Crotalus | oreganus | lutosus | USA | Utah | Box Elder |
| SDSNH | 33933 | Crotalus | oreganus | lutosus | USA | Utah | Box Elder |
| SDSNH | 33934 | Crotalus | oreganus | lutosus | USA | Utah | Box Elder |
| SDSNH | 33935 | Crotalus | oreganus | lutosus | USA | Utah | Box Elder |
| SDSNH | 33936 | Crotalus | oreganus | lutosus | USA | Utah | Box Elder |
| SDSNH | 33937 | Crotalus | oreganus | lutosus | USA | Utah | Box Elder |
| SDSNH | 33938 | Crotalus | oreganus | lutosus | USA | Utah | Box Elder |
| SDSNH | 33939 | Crotalus | oreganus | lutosus | USA | Utah | Box Elder |
| SDSNH | 34080 | Crotalus | oreganus | helleri | USA | California | San Diego |
| SDSNH | 34081 | Crotalus | oreganus | helleri | USA | California | San Diego |
| SDSNH | 34082 | Crotalus | oreganus | oreganus | USA | California | Fresno |
| SDSNH | 34243 | Crotalus | oreganus | helleri | USA | California | Santa Barbara |
| SDSNH | 34312 | Crotalus | oreganus | helleri | USA | California | San Diego |
| SDSNH | 34340 | Crotalus | viridis | viridis | USA | South Dakota | Butte |
| SDSNH | 34341 | Crotalus | viridis | viridis | USA | Wyoming | Natrona |
| SDSNH | 34348 | Crotalus | oreganus | helleri | USA | California | San Diego |
| SDSNH | 34376 | Crotalus | oreganus | helleri | USA | California | San Diego |
| SDSNH | 34445 | Crotalus | viridis | nuntius | USA | Arizona | Coconino |
| SDSNH | 34446 | Crotalus | viridis | nuntius | USA | Arizona | Coconino |
| SDSNH | 34568 | Crotalus | oreganus | helleri | USA | California | San Diego |
| SDSNH | 34568 | Crotalus | oreganus | helleri | USA | California | San Diego |
| SDSNH | 34636 | Crotalus | oreganus | helleri | USA | California | San Diego |
| SDSNH | 34651 | Crotalus | viridis | viridis | USA | Montana | Sweetgrass |
| SDSNH | 34652 | Crotalus | viridis | viridis | USA | Montana | Blaine |
| SDSNH | 34653 | Crotalus | viridis | viridis | USA | Montana | Blaine |
| SDSNH | 34654 | Crotalus | viridis | nuntius | USA | Arizona | Coconino |
| SDSNH | 34655 | Crotalus | viridis | nuntius | USA | Arizona | Coconino |
| SDSNH | 34656 | Crotalus | viridis | nuntius | USA | Arizona | Coconino |
| SDSNH | 34778 | Crotalus | viridis | nuntius | USA | Arizona | Coconino |
| SDSNH | 34784 | Crotalus | oreganus | lutosus | USA | Utah | Box Elder |
| SDSNH | 35100 | Crotalus | oreganus | helleri | USA | California | San Diego |
| SDSNH | 35300 | Crotalus | oreganus | helleri | USA | California | San Diego |
| SDSNH | 35312 | Crotalus | oreganus | helleri | USA | California | San Diego |
| SDSNH | 35313 | Crotalus | oreganus | helleri | USA | California | San Diego |
| SDSNH | 35347 | Crotalus | oreganus | oreganus | USA | California | Monterey |
| SDSNH | 35398 | Crotalus | oreganus | oreganus | USA | California | San Luis Obispo |
| SDSNH | 35399 | Crotalus | oreganus | oreganus | USA | California | San Luis Obispo |
| SDSNH | 35462 | Crotalus | oreganus | oreganus | USA | Idaho | Washington |
| SDSNH | 35463 | Crotalus | oreganus | oreganus | USA | Idaho | Washington |
| SDSNH | 35464 | Crotalus | oreganus | oreganus | USA | Idaho | Washington |
| SDSNH | 35636 | Crotalus | oreganus | helleri | USA | California | San Diego |
| SDSNH | 35669 | Crotalus | oreganus | helleri | USA | California | San Diego |
| SDSNH | 35670 | Crotalus | oreganus | helleri | USA | California | San Diego |
| SDSNH | 35834 | Crotalus | oreganus | helleri | USA | California | San Diego |
| SDSNH | 35835 | Crotalus | oreganus | oreganus | USA | California | Monterey |
| SDSNH | 36093 | Crotalus | oreganus | oreganus | USA | Oregon | Harney |
| SDSNH | 36094 | Crotalus | oreganus | oreganus | USA | Oregon | Harney |
| SDSNH | 36095 | Crotalus | oreganus | oreganus | USA | Oregon | Harney |
| SDSNH | 36096 | Crotalus | oreganus | oreganus | USA | Oregon | Harney |
| SDSNH | 36097 | Crotalus | viridis | viridis | USA | Iowa | Plymouth |
| SDSNH | 36098 | Crotalus | viridis | viridis | USA | Iowa | Plymouth |
| SDSNH | 36099 | Crotalus | viridis | viridis | USA | Iowa | Plymouth |
| SDSNH | 36100 | Crotalus | viridis | viridis | USA | Iowa | Plymouth |
| SDSNH | 36101 | Crotalus | viridis | viridis | USA | Iowa | Plymouth |
| SDSNH | 36102 | Crotalus | viridis | viridis | USA | Iowa | Plymouth |
| SDSNH | 36103 | Crotalus | viridis | viridis | USA | Iowa | Plymouth |
| SDSNH | 36104 | Crotalus | viridis | viridis | USA | Iowa | Plymouth |
| SDSNH | 36105 | Crotalus | viridis | viridis | USA | Iowa | Plymouth |
| SDSNH | 36106 | Crotalus | viridis | viridis | USA | Iowa | Plymouth |
| SDSNH | 36107 | Crotalus | viridis | viridis | USA | Iowa | Plymouth |
| SDSNH | 36108 | Crotalus | viridis | viridis | USA | Iowa | Plymouth |
| SDSNH | 36109 | Crotalus | viridis | viridis | USA | Iowa | Plymouth |
| SDSNH | 36110 | Crotalus | viridis | viridis | USA | Iowa | Plymouth |
| SDSNH | 36111 | Crotalus | viridis | viridis | USA | Iowa | Plymouth |
| SDSNH | 36162 | Crotalus | oreganus | helleri | USA | California | San Diego |
| SDSNH | 36163 | Crotalus | oreganus | helleri | USA | California | San Diego |
| SDSNH | 36179 | Crotalus | oreganus | helleri | USA | California | San Diego |
| SDSNH | 36208 | Crotalus | viridis | viridis | USA | Texas | El Paso |
| SDSNH | 36209 | Crotalus | viridis | viridis | USA | Texas | El Paso |
| SDSNH | 36228 | Crotalus | oreganus | oreganus | USA | California | Monterey |
| SDSNH | 36229 | Crotalus | viridis | viridis | USA | Montana | Wheatland |
| SDSNH | 36252 | Crotalus | oreganus | helleri | USA | California | Riverside |
| SDSNH | 36355 | Crotalus | oreganus | helleri | USA | California | San Diego |
| SDSNH | 36371 | Crotalus | oreganus | lutosus | USA | Nevada | Washoe |
| SDSNH | 36398 | Crotalus | oreganus | oreganus | Canada | British Columbia |  |
| SDSNH | 36399 | Crotalus | oreganus | oreganus | Canada | British Columbia |  |
| SDSNH | 36399 | Crotalus | oreganus | oreganus | Canada | British Columbia |  |
| SDSNH | 36400 | Crotalus | oreganus | oreganus | Canada | British Columbia |  |
| SDSNH | 36401 | Crotalus | oreganus | oreganus | Canada | British Columbia |  |
| SDSNH | 36402 | Crotalus | oreganus | oreganus | Canada | British Columbia |  |
| SDSNH | 36403 | Crotalus | oreganus | oreganus | Canada | British Columbia |  |
| SDSNH | 36404 | Crotalus | oreganus | oreganus | Canada | British Columbia |  |
| SDSNH | 36406 | Crotalus | oreganus | oreganus | Canada | British Columbia |  |
| SDSNH | 36407 | Crotalus | oreganus | oreganus | Canada | British Columbia |  |
| SDSNH | 36409 | Crotalus | oreganus | oreganus | Canada | British Columbia |  |
| SDSNH | 36409 | Crotalus | oreganus | oreganus | Canada | British Columbia |  |
| SDSNH | 36410 | Crotalus | oreganus | oreganus | Canada | British Columbia |  |
| SDSNH | 36412 | Crotalus | oreganus | oreganus | Canada | British Columbia |  |
| SDSNH | 36413 | Crotalus | oreganus | oreganus | Canada | British Columbia |  |
| SDSNH | 36626 | Crotalus | oreganus | helleri | USA | California | San Diego |
| SDSNH | 36627 | Crotalus | oreganus | lutosus | USA | Nevada | Washoe |
| SDSNH | 36628 | Crotalus | oreganus | lutosus | USA | Nevada | Washoe |
| SDSNH | 36629 | Crotalus | oreganus | lutosus | USA | Nevada | Washoe |
| SDSNH | 36630 | Crotalus | oreganus | lutosus | USA | Nevada | Washoe |
| SDSNH | 36631 | Crotalus | oreganus | lutosus | USA | Nevada | Washoe |
| SDSNH | 36632 | Crotalus | oreganus | lutosus | USA | Nevada | Washoe |
| SDSNH | 36633 | Crotalus | oreganus | lutosus | USA | Nevada | Washoe |
| SDSNH | 36634 | Crotalus | oreganus | lutosus | USA | Nevada | Washoe |
| SDSNH | 36635 | Crotalus | oreganus | lutosus | USA | Nevada | Washoe |
| SDSNH | 36636 | Crotalus | oreganus | lutosus | USA | Nevada | Washoe |
| SDSNH | 36637 | Crotalus | oreganus | lutosus | USA | Nevada | Washoe |
| SDSNH | 36638 | Crotalus | oreganus | lutosus | USA | Nevada | Washoe |
| SDSNH | 36639 | Crotalus | oreganus | lutosus | USA | Nevada | Washoe |
| SDSNH | 36640 | Crotalus | oreganus | lutosus | USA | Nevada | Washoe |
| SDSNH | 36641 | Crotalus | oreganus | lutosus | USA | Nevada | Washoe |
| SDSNH | 36642 | Crotalus | oreganus | lutosus | USA | Nevada | Washoe |
| SDSNH | 36643 | Crotalus | oreganus | lutosus | USA | Nevada | Washoe |
| SDSNH | 36644 | Crotalus | oreganus | lutosus | USA | Nevada | Washoe |
| SDSNH | 36645 | Crotalus | oreganus | lutosus | USA | Nevada | Washoe |
| SDSNH | 36646 | Crotalus | oreganus | lutosus | USA | Nevada | Washoe |
| SDSNH | 36647 | Crotalus | oreganus | lutosus | USA | Nevada | Washoe |
| SDSNH | 36648 | Crotalus | oreganus | lutosus | USA | Nevada | Washoe |
| SDSNH | 36676 | Crotalus | oreganus | helleri | USA | California | San Diego |
| SDSNH | 36677 | Crotalus | oreganus | helleri | USA | California | San Diego |
| SDSNH | 36678 | Crotalus | oreganus | helleri | USA | California | San Diego |
| SDSNH | 36682 | Crotalus | viridis | viridis | USA | Texas | Terry |
| SDSNH | 37234 | Crotalus | oreganus | helleri | USA | California | Los Angeles |
| SDSNH | 37247 | Crotalus | viridis | nuntius | USA | Arizona | Coconino |
| SDSNH | 37268 | Crotalus | oreganus | helleri | USA | California | San Diego |
| SDSNH | 37268 | Crotalus | oreganus | helleri | USA | California | San Diego |
| SDSNH | 37325 | Crotalus | oreganus | helleri | USA | California | San Diego |
| SDSNH | 37349 | Crotalus | oreganus | oreganus | USA | California | Monterey |
| SDSNH | 37370 | Crotalus | oreganus | oreganus | USA | California | Monterey |
| SDSNH | 37398 | Crotalus | oreganus | helleri | USA | California | San Diego |
| SDSNH | 37450 | Crotalus | oreganus | oreganus | USA | California | San Luis Obispo |
| SDSNH | 37451 | Crotalus | oreganus | oreganus | USA | California | San Luis Obispo |
| SDSNH | 37452 | Crotalus | oreganus | oreganus | USA | California | San Luis Obispo |
| SDSNH | 37453 | Crotalus | oreganus | oreganus | USA | California | San Luis Obispo |
| SDSNH | 37454 | Crotalus | oreganus | oreganus | USA | California | San Luis Obispo |
| SDSNH | 37632 | Crotalus | oreganus | oreganus | USA | California | Monterey |
| SDSNH | 37715 | Crotalus | oreganus | oreganus | USA | California | Santa Barbara |
| SDSNH | 37749 | Crotalus | oreganus | helleri | USA | California | San Diego |
| SDSNH | 37970 | Crotalus | oreganus | oreganus | USA | California | Monterey |
| SDSNH | 37987 | Crotalus | oreganus | helleri | Mexico | Baja California Norte |  |
| SDSNH | 37998 | Crotalus | oreganus | helleri | USA | California | San Diego |
| SDSNH | 38185 | Crotalus | oreganus | oreganus | USA | California | Monterey |
| SDSNH | 38186 | Crotalus | oreganus | oreganus | USA | California | Monterey |
| SDSNH | 38214 | Crotalus | oreganus | oreganus | USA | California | Madera |
| SDSNH | 38218 | Crotalus | oreganus | oreganus | USA | California | Madera |
| SDSNH | 38219 | Crotalus | oreganus | oreganus | USA | California | Madera |
| SDSNH | 38221 | Crotalus | oreganus | oreganus | USA | California | Madera |
| SDSNH | 38222 | Crotalus | oreganus | oreganus | USA | California | Madera |
| SDSNH | 38223 | Crotalus | oreganus | oreganus | USA | California | Madera |
| SDSNH | 38224 | Crotalus | oreganus | oreganus | USA | California | Madera |
| SDSNH | 38225 | Crotalus | oreganus | oreganus | USA | California | Madera |
| SDSNH | 38226 | Crotalus | oreganus | oreganus | USA | California | Madera |
| SDSNH | 38227 | Crotalus | oreganus | oreganus | USA | California | Madera |
| SDSNH | 38227 | Crotalus | oreganus | oreganus | USA | California | Madera |
| SDSNH | 38228 | Crotalus | oreganus | oreganus | USA | California | Madera |
| SDSNH | 38229 | Crotalus | oreganus | oreganus | USA | California | Madera |
| SDSNH | 38230 | Crotalus | oreganus | oreganus | USA | California | Madera |
| SDSNH | 38231 | Crotalus | oreganus | oreganus | USA | California | Madera |
| SDSNH | 38232 | Crotalus | oreganus | oreganus | USA | California | Madera |
| SDSNH | 38233 | Crotalus | oreganus | oreganus | USA | California | Madera |
| SDSNH | 38303 | Crotalus | oreganus | helleri | USA | California | San Diego |
| SDSNH | 38374 | Crotalus | oreganus | lutosus | USA | Nevada | Washoe |
| SDSNH | 38375 | Crotalus | oreganus | lutosus | USA | Nevada | Washoe |
| SDSNH | 38393 | Crotalus | oreganus | helleri | USA | California | San Diego |
| SDSNH | 38394 | Crotalus | oreganus | helleri | USA | California | San Diego |
| SDSNH | 38670 | Crotalus | oreganus | helleri | USA | California | San Diego |
| SDSNH | 38838 | Crotalus | oreganus | helleri | USA | California | San Bernardino |
| SDSNH | 38839 | Crotalus | oreganus | helleri | USA | California | San Bernardino |
| SDSNH | 38855 | Crotalus | oreganus | helleri | USA | California | Orange |
| SDSNH | 38912 | Crotalus | oreganus | helleri | Mexico | Baja California Norte |  |
| SDSNH | 38913 | Crotalus | oreganus | helleri | Mexico | Baja California Norte |  |
| SDSNH | 38966 | Crotalus | oreganus | lutosus | USA | Nevada | Washoe |
| SDSNH | 38969 | Crotalus | oreganus | lutosus | USA | Nevada | Washoe |
| SDSNH | 38993 | Crotalus | oreganus | helleri | USA | California | San Diego |
| SDSNH | 39022 | Crotalus | oreganus | helleri | Mexico | Baja California Norte |  |
| SDSNH | 39026 | Crotalus | oreganus | helleri | Mexico | Baja California Norte |  |
| SDSNH | 39041 | Crotalus | oreganus | lutosus | USA | Nevada | Washoe |
| SDSNH | 39060 | Crotalus | oreganus | lutosus | USA | Nevada | Washoe |
| SDSNH | 39064 | Crotalus | oreganus | lutosus | USA | Nevada | Washoe |
| SDSNH | 39068 | Crotalus | oreganus | lutosus | USA | Nevada | Washoe |
| SDSNH | 39069 | Crotalus | oreganus | oreganus | USA | Idaho | Idaho |
| SDSNH | 39087 | Crotalus | oreganus | helleri | USA | California | San Diego |
| SDSNH | 39242 | Crotalus | oreganus | oreganus | USA | California | Lassen |
| SDSNH | 39243 | Crotalus | oreganus | oreganus | USA | California | Lassen |
| SDSNH | 39244 | Crotalus | oreganus | oreganus | USA | California | Lassen |
| SDSNH | 39245 | Crotalus | oreganus | lutosus | USA | Nevada | Washoe |
| SDSNH | 39246 | Crotalus | oreganus | lutosus | USA | Nevada | Washoe |
| SDSNH | 39247 | Crotalus | oreganus | lutosus | USA | Nevada | Washoe |
| SDSNH | 39248 | Crotalus | oreganus | lutosus | USA | Nevada | Washoe |
| SDSNH | 39249 | Crotalus | oreganus | lutosus | USA | Nevada | Washoe |
| SDSNH | 39250 | Crotalus | oreganus | lutosus | USA | Nevada | Washoe |
| SDSNH | 39252 | Crotalus | oreganus | lutosus | USA | Nevada | Washoe |
| SDSNH | 39269 | Crotalus | oreganus | lutosus | Nevada | Washoe |  |
| SDSNH | 39270 | Crotalus | oreganus | lutosus | Nevada | Washoe |  |
| SDSNH | 39271 | Crotalus | oreganus | lutosus | Nevada | Washoe |  |
| SDSNH | 39272 | Crotalus | oreganus | lutosus | Nevada | Washoe |  |
| SDSNH | 39273 | Crotalus | oreganus | lutosus | Nevada | Washoe |  |
| SDSNH | 39283 | Crotalus | oreganus | lutosus | USA | Nevada | Nye |
| SDSNH | 39284 | Crotalus | oreganus | lutosus | USA | Nevada | Nye |
| SDSNH | 39580 | Crotalus | oreganus | oreganus | USA | California | Lassen |
| SDSNH | 39581 | Crotalus | oreganus | oreganus | USA | California | Lassen |
| SDSNH | 39582 | Crotalus | oreganus | oreganus | USA | California | Lassen |
| SDSNH | 39584 | Crotalus | oreganus | oreganus | USA | California | Lassen |
| SDSNH | 39585 | Crotalus | oreganus | oreganus | USA | California | Lassen |
| SDSNH | 39587 | Crotalus | oreganus | lutosus | USA | Nevada | Washoe |
| SDSNH | 39588 | Crotalus | oreganus | lutosus | USA | Nevada | Washoe |
| SDSNH | 39593 | Crotalus | oreganus | lutosus | USA | Nevada | Washoe |
| SDSNH | 39595 | Crotalus | viridis | viridis | USA | South Dakota | Mellette |
| SDSNH | 39596 | Crotalus | viridis | viridis | USA | South Dakota | Mellette |
| SDSNH | 39597 | Crotalus | viridis | viridis | USA | South Dakota | Mellette |
| SDSNH | 39598 | Crotalus | viridis | viridis | USA | South Dakota | Mellette |
| SDSNH | 39599 | Crotalus | viridis | viridis | USA | South Dakota | Mellette |
| SDSNH | 39651 | Crotalus | oreganus | helleri | USA | California | Los Angeles |
| SDSNH | 39670 | Crotalus | viridis | viridis | USA | South Dakota | Mellette |
| SDSNH | 39671 | Crotalus | viridis | viridis | USA | South Dakota | Mellette |
| SDSNH | 39671 | Crotalus | viridis | viridis | USA | South Dakota | Mellette |
| SDSNH | 39673 | Crotalus | viridis | viridis | USA | South Dakota | Mellette |
| SDSNH | 39674 | Crotalus | viridis | viridis | USA | South Dakota | Mellette |
| SDSNH | 39675 | Crotalus | viridis | viridis | USA | South Dakota | Mellette |
| SDSNH | 39676 | Crotalus | viridis | viridis | USA | South Dakota | Mellette |
| SDSNH | 39677 | Crotalus | oreganus | oreganus | USA | California | Madera |
| SDSNH | 39699 | Crotalus | oreganus | helleri | USA | California | San Diego |
| SDSNH | 39701 | Crotalus | oreganus | helleri | USA | California | San Diego |
| SDSNH | 39730 | Crotalus | oreganus | helleri | Mexico | Baja California Norte |  |
| SDSNH | 39731 | Crotalus | oreganus | helleri | USA | California | San Diego |
| SDSNH | 39791 | Crotalus | viridis | viridis | USA | South Dakota | Mellette |
| SDSNH | 39805 | Crotalus | oreganus | helleri | USA | California | San Diego |
| SDSNH | 39806 | Crotalus | oreganus | helleri | Mexico | Baja California Norte |  |
| SDSNH | 39832 | Crotalus | oreganus | helleri | Mexico | Baja California Norte |  |
| SDSNH | 39844 | Crotalus | oreganus | helleri | USA | California | Santa Barbara |
| SDSNH | 39845 | Crotalus | oreganus | helleri | USA | California | Santa Barbara |
| SDSNH | 39846 | Crotalus | oreganus | helleri | USA | California | Santa Barbara |
| SDSNH | 39864 | Crotalus | oreganus | helleri | USA | California | Riverside |
| SDSNH | 39944 | Crotalus | oreganus | helleri | USA | California | San Diego |
| SDSNH | 39945 | Crotalus | oreganus | oreganus | USA | California | San Luis Obispo |
| SDSNH | 39946 | Crotalus | oreganus | helleri | Mexico | Baja California Norte |  |
| SDSNH | 39946 | Crotalus | oreganus | helleri | Mexico | Baja California Norte |  |
| SDSNH | 40107 | Crotalus | oreganus | helleri | USA | California | San Diego |
| SDSNH | 40140 | Crotalus | oreganus | helleri | Mexico | Baja California Norte |  |
| SDSNH | 40267 | Crotalus | oreganus | oreganus | USA | California | Kern |
| SDSNH | 40300 | Crotalus | oreganus | helleri | USA | California | San Diego |
| SDSNH | 40532 | Crotalus | oreganus | oreganus | USA | California | Nevada |
| SDSNH | 40533 | Crotalus | oreganus | oreganus | USA | California | Nevada |
| SDSNH | 40534 | Crotalus | oreganus | oreganus | USA | California | Nevada |
| SDSNH | 40535 | Crotalus | oreganus | oreganus | USA | California | Nevada |
| SDSNH | 40685 | Crotalus | viridis | nuntius | USA | Arizona | Coconino |
| SDSNH | 40750 | Crotalus | oreganus | helleri | USA | California | San Diego |
| SDSNH | 40751 | Crotalus | oreganus | caliginis | Unknown | Unknown | Unknown |
| SDSNH | 40787 | Crotalus | oreganus | helleri | USA | California | San Diego |
| SDSNH | 40868 | Crotalus | oreganus | helleri | USA | California | San Diego |
| SDSNH | 40871 | Crotalus | viridis | nuntius | USA | Arizona | Navajo |
| SDSNH | 40872 | Crotalus | viridis | nuntius | USA | Arizona | Navajo |
| SDSNH | 40874 | Crotalus | oreganus | cerberus | USA | Arizona | Navajo |
| SDSNH | 41108 | Crotalus | oreganus | oreganus | USA | California | Monterey |
| SDSNH | 41186 | Crotalus | oreganus | helleri | USA | California | San Diego |
| SDSNH | 41190 | Crotalus | oreganus | helleri | USA | California | San Diego |
| SDSNH | 41191 | Crotalus | oreganus | helleri | USA | California | San Diego |
| SDSNH | 41198 | Crotalus | oreganus | caliginis | Mexico | Baja California Norte |  |
| SDSNH | 41230 | Crotalus | oreganus | helleri | USA | California | San Diego |
| SDSNH | 41230 | Crotalus | oreganus | helleri | USA | California | San Diego |
| SDSNH | 41265 | Crotalus | oreganus | helleri | USA | California | San Diego |
| SDSNH | 41336 | Crotalus | oreganus | oreganus | USA | California | Kings |
| SDSNH | 41467 | Crotalus | oreganus | oreganus | USA | California | Napa |
| SDSNH | 41520 | Crotalus | oreganus | helleri | USA | California | San Diego |
| SDSNH | 41532 | Crotalus | oreganus | helleri | Mexico | Baja California Norte |  |
| SDSNH | 41752 | Crotalus | oreganus | helleri | USA | California | San Diego |
| SDSNH | 41758 | Crotalus | oreganus | oreganus | USA | California | Kings |
| SDSNH | 42017 | Crotalus | oreganus | helleri | Mexico | Baja California Norte |  |
| SDSNH | 42018 | Crotalus | oreganus | helleri | Mexico | Baja California Norte |  |
| SDSNH | 42074 | Crotalus | oreganus | oreganus | USA | California | Kern |
| SDSNH | 42106 | Crotalus | oreganus | helleri | USA | California | San Diego |
| SDSNH | 42162 | Crotalus | oreganus | oreganus | USA | Oregon | Malheur |
| SDSNH | 42199 | Crotalus | oreganus | oreganus | USA | California | Plumas |
| SDSNH | 42200 | Crotalus | oreganus | oreganus | USA | California | Plumas |
| SDSNH | 42201 | Crotalus | oreganus | helleri | USA | California | Santa Barbara |
| SDSNH | 42298 | Crotalus | oreganus | helleri | USA | California | Los Angeles |
| SDSNH | 42314 | Crotalus | oreganus | oreganus | USA | California | Kings |
| SDSNH | 42331 | Crotalus | oreganus | oreganus | USA | California | Merced |
| SDSNH | 42332 | Crotalus | oreganus | oreganus | USA | California | Merced |
| SDSNH | 42333 | Crotalus | oreganus | oreganus | USA | California | Merced |
| SDSNH | 42334 | Crotalus | oreganus | oreganus | USA | California | Merced |
| SDSNH | 42335 | Crotalus | oreganus | oreganus | USA | California | Merced |
| SDSNH | 42336 | Crotalus | oreganus | oreganus | USA | California | Merced |
| SDSNH | 42352 | Crotalus | oreganus | oreganus | USA | California | Kern |
| SDSNH | 42353 | Crotalus | oreganus | oreganus | USA | California | Merced |
| SDSNH | 42380 | Crotalus | oreganus | helleri | USA | California | Santa Barbara |
| SDSNH | 42381 | Crotalus | oreganus | helleri | USA | California | Santa Barbara |
| SDSNH | 42397 | Crotalus | oreganus | helleri | USA | California | San Diego |
| SDSNH | 42548 | Crotalus | oreganus | helleri | USA | California | San Diego |
| SDSNH | 42550 | Crotalus | oreganus | helleri | Mexico | Baja California Norte |  |
| SDSNH | 42551 | Crotalus | oreganus | helleri | USA | California | San Diego |
| SDSNH | 42552 | Crotalus | oreganus | helleri | USA | California | Los Angeles |
| SDSNH | 42569 | Crotalus | oreganus | helleri | USA | California | San Diego |
| SDSNH | 42672 | Crotalus | oreganus | helleri | USA | California | San Diego |
| SDSNH | 42691 | Crotalus | oreganus | helleri | Mexico | Baja California Norte |  |
| SDSNH | 42715 | Crotalus | oreganus | helleri | USA | California | San Diego |
| SDSNH | 42810 | Crotalus | oreganus | oreganus | USA | California | San Bernardino |
| SDSNH | 42811 | Crotalus | oreganus | oreganus | USA | California | San Bernardino |
| SDSNH | 42812 | Crotalus | oreganus | helleri | USA | California | San Diego |
| SDSNH | 42818 | Crotalus | oreganus | oreganus | USA | Washington | Garfield |
| SDSNH | 42859 | Crotalus | oreganus | caliginis | Mexico | Baja California Norte |  |
| SDSNH | 42868 | Crotalus | oreganus | helleri | USA | California | San Diego |
| SDSNH | 42880 | Crotalus | oreganus | helleri | Mexico | Baja California Norte |  |
| SDSNH | 43096 | Crotalus | oreganus | oreganus | USA | California | San Bernardino |
| SDSNH | 43097 | Crotalus | oreganus | oreganus | USA | California | San Bernardino |
| SDSNH | 43099 | Crotalus | oreganus | oreganus | USA | California | Kern |
| SDSNH | 43102 | Crotalus | oreganus | helleri | USA | California | San Diego |
| SDSNH | 43176 | Crotalus | oreganus | oreganus | USA | California | San Bernardino |
| SDSNH | 43200 | Crotalus | oreganus | helleri | USA | California | San Diego |
| SDSNH | 43249 | Crotalus | oreganus | helleri | USA | California | San Diego |
| SDSNH | 43250 | Crotalus | oreganus | helleri | USA | California | San Diego |
| SDSNH | 43287 | Crotalus | oreganus | helleri | USA | California | San Diego |
| SDSNH | 43290 | Crotalus | oreganus | helleri | USA | California | San Diego |
| SDSNH | 43314 | Crotalus | oreganus | helleri | USA | California | San Diego |
| SDSNH | 43331 | Crotalus | oreganus | helleri | USA | California | San Diego |
| SDSNH | 43332 | Crotalus | oreganus | oreganus | USA | California | Kern |
| SDSNH | 43347 | Crotalus | oreganus | helleri | USA | California | San Diego |
| SDSNH | 43348 | Crotalus | oreganus | helleri | USA | California | San Diego |
| SDSNH | 43355 | Crotalus | oreganus | oreganus | USA | California | Placer |
| SDSNH | 43356 | Crotalus | oreganus | helleri | USA | California | Riverside |
| SDSNH | 43381 | Crotalus | oreganus | lutosus | USA | Nevada | Washoe |
| SDSNH | 43393 | Crotalus | oreganus | lutosus | USA | Nevada | Washoe |
| SDSNH | 43396 | Crotalus | oreganus | helleri | USA | California | San Diego |
| SDSNH | 43397 | Crotalus | oreganus | helleri | USA | California | San Diego |
| SDSNH | 43406 | Crotalus | oreganus | helleri | USA | California | San Diego |
| SDSNH | 43411 | Crotalus | oreganus | helleri | USA | California | Los Angeles |
| SDSNH | 43468 | Crotalus | oreganus | oreganus | USA | California | Kern |
| SDSNH | 43469 | Crotalus | oreganus | oreganus | USA | California | Kern |
| SDSNH | 43569 | Crotalus | oreganus | helleri | USA | California | San Diego |
| SDSNH | 43583 | Crotalus | oreganus | helleri | USA | California | San Diego |
| SDSNH | 43584 | Crotalus | viridis | viridis | USA | New Mexico | Bernalillo |
| SDSNH | 43585 | Crotalus | viridis | viridis | USA | New Mexico | Bernalillo |
| SDSNH | 43586 | Crotalus | viridis | viridis | USA | New Mexico | Bernalillo |
| SDSNH | 43587 | Crotalus | viridis | viridis | USA | New Mexico | Bernalillo |
| SDSNH | 43604 | Crotalus | oreganus | helleri | USA | California | Riverside |
| SDSNH | 43605 | Crotalus | oreganus | cerberus | USA | Arizona | Yavapai |
| SDSNH | 43730 | Crotalus | oreganus | lutosus | USA | Nevada | Nye |
| SDSNH | 43731 | Crotalus | oreganus | lutosus | USA | Nevada | Lincoln |
| SDSNH | 43732 | Crotalus | oreganus | lutosus | USA | Nevada | Lincoln |
| SDSNH | 43733 | Crotalus | viridis | nuntius | USA | Utah | San Juan |
| SDSNH | 43736 | Crotalus | oreganus | cerberus | USA | Arizona | Yavapai |
| SDSNH | 43738 | Crotalus | oreganus | cerberus | USA | Arizona | Yavapai |
| SDSNH | 43743 | Crotalus | oreganus | lutosus | USA | Nevada | Washoe |
| SDSNH | 43780 | Crotalus | oreganus | helleri | USA | California | San Diego |
| SDSNH | 43781 | Crotalus | oreganus | helleri | USA | California | San Diego |
| SDSNH | 43802 | Crotalus | oreganus | helleri | USA | California | San Diego |
| SDSNH | 43951 | Crotalus | oreganus | lutosus | USA | Utah | San Juan |
| SDSNH | 43998 | Crotalus | oreganus | helleri | USA | California | San Diego |
| SDSNH | 44070 | Crotalus | oreganus | helleri | USA | California | San Diego |
| SDSNH | 44078 | Crotalus | oreganus | helleri | USA | California | San Diego |
| SDSNH | 44079 | Crotalus | oreganus | helleri | USA | California | San Diego |
| SDSNH | 44090 | Crotalus | oreganus | helleri | Mexico | Baja California Norte |  |
| SDSNH | 44111 | Crotalus | oreganus | helleri | Mexico | Baja California Norte |  |
| SDSNH | 44113 | Crotalus | oreganus | helleri | USA | California | San Diego |
| SDSNH | 44114 | Crotalus | oreganus | helleri | USA | California | San Diego |
| SDSNH | 44182 | Crotalus | oreganus | helleri | USA | California | San Diego |
| SDSNH | 44193 | Crotalus | oreganus | helleri | USA | California | San Diego |
| SDSNH | 44193 | Crotalus | oreganus | helleri | USA | California | San Diego |
| SDSNH | 44194 | Crotalus | oreganus | helleri | USA | California | San Diego |
| SDSNH | 44195 | Crotalus | oreganus | helleri | USA | California | San Diego |
| SDSNH | 44199 | Crotalus | oreganus | helleri | USA | California | San Diego |
| SDSNH | 44206 | Crotalus | oreganus | helleri | USA | California | San Diego |
| SDSNH | 44260 | Crotalus | oreganus | helleri | USA | California | San Diego |
| SDSNH | 44331 | Crotalus | oreganus | oreganus | USA | Oregon | Harney |
| SDSNH | 44332 | Crotalus | oreganus | oreganus | USA | Oregon | Harney |
| SDSNH | 45976 | Crotalus | oreganus | helleri | Mexico | Baja California Norte |  |
| SDSNH | 46185 | Crotalus | oreganus | lutosus | USA | California | Mono |
| SDSNH | 48057 | Crotalus | oreganus | helleri | Mexico | Baja California Norte |  |
| SDSNH | 48061 | Crotalus | oreganus | helleri | Mexico | Baja California Norte |  |
| SDSNH | 48062 | Crotalus | oreganus | helleri | Mexico | Baja California Norte |  |
| SDSNH | 48149 | Crotalus | oreganus | helleri | Mexico | Baja California Norte |  |
| SDSNH | 48717 | Crotalus | oreganus | helleri | USA | California | San Diego |
| SDSNH | 49725 | Crotalus | oreganus | helleri | USA | California | San Diego |
| SDSNH | 49728 | Crotalus | oreganus | helleri | USA | California | San Diego |
| SDSNH | 49729 | Crotalus | oreganus | helleri | USA | California | San Diego |
| SDSNH | 49730 | Crotalus | oreganus | helleri | USA | California | San Diego |
| SDSNH | 49960 | Crotalus | oreganus | caliginis | Mexico | Baja California Norte |  |
| SDSNH | 52706 | Crotalus | oreganus | helleri | USA | California | San Diego |
| SDSNH | 52742 | Crotalus | oreganus | helleri | USA | California | San Diego |
| SDSNH | 53150 | Crotalus | oreganus | helleri | USA | California | San Diego |
| SDSNH | 57473 | Crotalus | oreganus | helleri | Mexico | Baja California Norte |  |
| SDSNH | 57474 | Crotalus | oreganus | helleri | Mexico | Baja California Norte |  |
| SDSNH | 57861 | Crotalus | viridis | viridis | USA | New Mexico | Rio Arriba |
| SDSNH | 58234 | Crotalus | oreganus | helleri | USA | California | Santa Barbara |
| SDSNH | 58234 | Crotalus | oreganus | helleri | USA | California | Santa Barbara |
| SDSNH | 58234 | Crotalus | oreganus | helleri | USA | California | Santa Barbara |
| SDSNH | 58235 | Crotalus | oreganus | helleri | USA | California | Santa Barbara |
| SDSNH | 58236 | Crotalus | oreganus | helleri | USA | California | Santa Barbara |
| SDSNH | 58237 | Crotalus | oreganus | helleri | USA | California | Santa Barbara |
| SDSNH | 58238 | Crotalus | oreganus | helleri | USA | California | Santa Barbara |
| SDSNH | 58240 | Crotalus | oreganus | helleri | USA | California | Santa Barbara |
| SDSNH | 58241 | Crotalus | oreganus | helleri | USA | California | Santa Barbara |
| SDSNH | 58242 | Crotalus | oreganus | helleri | USA | California | Santa Barbara |
| SDSNH | 58244 | Crotalus | oreganus | helleri | USA | California | Santa Barbara |
| SDSNH | 58245 | Crotalus | oreganus | helleri | USA | California | Santa Barbara |
| SDSNH | 58246 | Crotalus | oreganus | helleri | USA | California | Santa Barbara |
| SDSNH | 58247 | Crotalus | oreganus | helleri | USA | California | Santa Barbara |
| SDSNH | 58440 | Crotalus | oreganus | helleri | USA | California | Santa Barbara |
| SDSNH | 58440 | Crotalus | oreganus | helleri | USA | California | Santa Barbara |
| SDSNH | 58515 | Crotalus | oreganus | helleri | USA | California | Santa Barbara |
| SDSNH | 67235 | Crotalus | oreganus | helleri | USA | California | San Diego |
| SDSNH | 68917 | Crotalus | viridis | viridis | USA | New Mexico | Valencia |
| SDSNH | 68960 | Crotalus | oreganus | helleri | USA | California | Riverside |
| SDSNH | 68961 | Crotalus | oreganus | helleri | USA | California | Riverside |
| SDSNH | 72176 | Crotalus | oreganus | oreganus | USA | California | Santa Barbara |
| SDSNH | 72177 | Crotalus | oreganus | oreganus | USA | California | San Bernardino |
| SDSNH | 72708 | Crotalus | oreganus | helleri | USA | California | San Diego |
| SDSNH | 78376 | Crotalus | oreganus | lutosus |  |  |  |
| SDSNH | 89700 | Crotalus | oreganus | helleri | USA | California |  |
| UAZ | 9331 | Crotalus | oreganus | cerberus | USA | Arizona | Pima |
| UAZ | 14706 | Crotalus | oreganus | lutosus | USA | Arizona | Mohave |
| UAZ | 23304 | Crotalus | oreganus | helleri | Mexico | Baja |  |
| UAZ | 23305 | Crotalus | oreganus | helleri | Mexico | Baja |  |
| UAZ | 27337 | Crotalus | viridis | viridis | USA |  |  |
| UAZ | 27851 | Crotalus | oreganus | cerberus | USA | Arizona | Yavapai |
| UAZ | 27852 | Crotalus | oreganus | cerberus | USA | Arizona | Navajo |
| UAZ | 27853 | Crotalus | viridis | nuntius | USA | Arizona | Navajo |
| UAZ | 27854 | Crotalus | oreganus | cerberus | USA | Arizona | Pima |
| UAZ | 27855 | Crotalus | oreganus | abyssus | USA | Arizona | Coconino |
| UAZ | 27857 | Crotalus | oreganus | cerberus | USA | Arizona | Mohave |
| UAZ | 27858 | Crotalus | oreganus | cerberus | USA | Arizona | Mohave |
| UAZ | 27859 | Crotalus | oreganus | cerberus | USA | Arizona | Pima |
| UAZ | 27860 | Crotalus | oreganus | cerberus | USA | Arizona | Gila |
| UAZ | 27861 | Crotalus | oreganus | abyssus | USA | Arizona | Coconino |
| UAZ | 27863 | Crotalus | viridis | nuntius | USA | Arizona | Coconino |
| UAZ | 27864 | Crotalus | oreganus | lutosus | USA | Arizona |  |
| UAZ | 27865 | Crotalus | oreganus | lutosus | USA | Arizona | Mohave |
| UAZ | 27866 | Crotalus | oreganus | cerberus | USA | Arizona | Yavapai |
| UAZ | 27867 | Crotalus | oreganus | cerberus | USA | Arizona | Yavapai |
| UAZ | 27870 | Crotalus | oreganus | cerberus | USA | Arizona | Yavapai |
| UAZ | 27871 | Crotalus | oreganus | cerberus | USA | Arizona | Pinal |
| UAZ | 27872 | Crotalus | oreganus | cerberus | USA | Arizona | Coconino |
| UAZ | 27873 | Crotalus | oreganus | lutosus | USA | Arizona | Coconino |
| UAZ | 27874 | Crotalus | oreganus | lutosus | USA | Arizona | Coconino |
| UAZ | 27876 | Crotalus | oreganus | cerberus | USA | Arizona | Gila |
| UAZ | 27877 | Crotalus | oreganus | nuntius | USA | Arizona | Navajo |
| UAZ | 27878 | Crotalus | viridis | nuntius | USA | Arizona | Coconino |
| UAZ | 27879 | Crotalus | oreganus | nuntius | USA | Arizona | Apache |
| UAZ | 27881 | Crotalus | viridis | nuntius | USA | Arizona | Apache |
| UAZ | 27882 | Crotalus | oreganus | nuntius | USA | Arizona | Coconino |
| UAZ | 27884 | Crotalus | oreganus | nuntius | USA | Arizona | Coconino |
| UAZ | 27885 | Crotalus | oreganus | cerberus | USA | Arizona | Coconino |
| UAZ | 27886 | Crotalus | oreganus | nuntius | USA | Arizona | Coconino |
| UAZ | 27887 | Crotalus | viridis | viridis | USA | Colorado |  |
| UAZ | 27888 | Crotalus | viridis | viridis | USA | Colorado |  |
| UAZ | 27889 | Crotalus | viridis | viridis | USA | Colorado |  |
| UAZ | 27890 | Crotalus | viridis | viridis | USA | Colorado |  |
| UAZ | 27891 | Crotalus | oreganus | lutosus | USA | Idaho |  |
| UAZ | 27892 | Crotalus | oreganus | lutosus | USA | Idaho |  |
| UAZ | 27914 | Crotalus |  |  |  |  |  |
| UAZ | 27916 | Crotalus | oreganus | lutosus | USA | Nevada |  |
| UAZ | 27917 | Crotalus | viridis | viridis | USA |  |  |
| UAZ | 27918 | Crotalus | viridis | viridis | USA |  |  |
| UAZ | 27919 | Crotalus | viridis | viridis | USA |  |  |
| UAZ | 27920 | Crotalus | oreganus | cerberus | USA | Arizona | Pima |
| UAZ | 27921 | Crotalus | oreganus | cerberus | USA | Arizona | Pima |
| UAZ | 27922 | Crotalus | oreganus | cerberus | USA | Arizona | Pinal |
| UAZ | 27923 | Crotalus | oreganus | cerberus | USA | Arizona | Pima |
| UAZ | 27924 | Crotalus | oreganus | cerberus | USA | Arizona | Cochise |
| UAZ | 27925 | Crotalus | oreganus | cerberus | USA | Arizona | Graham |
| UAZ | 27927 | Crotalus | oreganus | oreganus | USA |  |  |
| UAZ | 27932 | Crotalus | oreganus | oreganus | USA | Utah |  |
| UAZ | 28026 | Crotalus | oreganus | cerberus | USA | Arizona | Pima |
| UAZ | 28392 | Crotalus | viridis | nuntius | USA | Arizona | Apache |
| UAZ | 28398 | Crotalus | viridis | nuntius | USA | Arizona | Apache |
| UAZ | 28399 | Crotalus | viridis | nuntius | USA | Arizona | Apache |
| UAZ | 29862 | Crotalus | oreganus | cerberus | USA | Arizona |  |
| UAZ | 30155 | Crotalus | viridis | viridis | USA |  |  |
| UAZ | 30220 | Crotalus | viridis | viridis | USA |  |  |
| UAZ | 30431 | Crotalus | oreganus | oreganus | USA |  |  |
| UAZ | 30432 | Crotalus | oreganus | oreganus | USA |  |  |
| UAZ | 30433 | Crotalus | oreganus | oreganus | USA |  |  |
| UAZ | 30434 | Crotalus | oreganus | oreganus | USA |  |  |
| UAZ | 30435 | Crotalus | oreganus | oreganus | USA |  |  |
| UAZ | 30436 | Crotalus | oreganus | oreganus | USA |  |  |
| UAZ | 30437 | Crotalus | oreganus | oreganus | USA |  |  |
| UAZ | 30438 | Crotalus | oreganus | oreganus | USA |  |  |
| UAZ | 30440 | Crotalus | oreganus | oreganus | USA | Washington |  |
| UAZ | 30441 | Crotalus | oreganus | oreganus | USA | Washington |  |
| UAZ | 30442 | Crotalus | oreganus | oreganus | USA | Washington |  |
| UAZ | 30443 | Crotalus | oreganus | oreganus | USA | Washington |  |
| UAZ | 31317 | Crotalus | viridis | nuntius | USA | Arizona | Apache |
| UAZ | 32195 | Crotalus | oreganus | oreganus | USA | Oregon |  |
| UAZ | 32364 | Crotalus | oreganus | lutosus | USA | Arizona |  |
| UAZ | 32620 | Crotalus | viridis | nuntius | USA | Arizona | Coconino |
| UAZ | 32942 | Crotalus | oreganus | abyssus | USA | Arizona | Coconino |
| UAZ | 32999 | Crotalus | oreganus | cerberus | USA | Arizona | Pima |
| UAZ | 33000 | Crotalus | oreganus | lutosus | USA | Arizona | Mohave |
| UAZ | 33449 | Crotalus | viridis | nuntius | USA | Arizona | Apache |
| UAZ | 33450 | Crotalus | viridis | nuntius | USA | Arizona | Apache |
| UAZ | 33522 | Crotalus | viridis | nuntius | USA | Arizona | Apache |
| UAZ | 33637 | Crotalus | viridis | nuntius | USA | Arizona | Apache |
| UAZ | 34031 | Crotalus | viridis | cerberus | USA | Arizona |  |
| UAZ | 34392 | Crotalus | viridis | nuntius | USA | Arizona | Navajo |
| UAZ | 34782 | Crotalus | viridis | nuntius | USA | Arizona | Coconino |
| UAZ | 35379 | Crotalus | viridis | nuntius | USA | Arizona |  |
| UAZ | 35986 | Crotalus | oreganus | cerberus | USA | Arizona | Pima |
| UAZ | 35987 | Crotalus | oreganus | cerberus | USA | Arizona | Pima |
| UAZ | 35988 | Crotalus | oreganus | cerberus | USA | Arizona | Yavapai |
| UAZ | 35989 | Crotalus | oreganus | cerberus | USA | Arizona | Navajo |
| UAZ | 35990 | Crotalus | oreganus | nuntius | USA | Arizona | Navajo |
| UAZ | 35991 | Crotalus | oreganus | nuntius | USA | Arizona | Navajo |
| UAZ | 35992 | Crotalus | oreganus | nuntius | USA | Arizona | Navajo |
| UAZ | 35994 | Crotalus | viridis | nuntius | USA | Arizona | Navajo |
| UAZ | 35995 | Crotalus | viridis | nuntius | USA | Arizona | Coconino |
| UAZ | 36325 | Crotalus | oreganus | lutosus | USA | Arizona | Mohave |
| UAZ | 37755 | Crotalus | viridis | viridis | USA | New Mexico |  |
| UAZ | 37756 | Crotalus | viridis | viridis | USA | Colorado |  |
| UAZ | 37845 | Crotalus | viridis | nuntius | USA | Arizona | Navajo |
| UAZ | 37846 | Crotalus | viridis | nuntius | USA | Arizona | Navajo |
| UAZ | 38358 | Crotalus | viridis | viridis | USA | New Mexico |  |
| UAZ | 38359 | Crotalus | viridis | viridis | USA | New Mexico |  |
| UAZ | 38483 | Crotalus | viridis | viridis | USA |  |  |
| UAZ | 38502 | Crotalus | viridis | nuntius | USA | Arizona | Apache |
| UAZ | 38503 | Crotalus | viridis | nuntius | USA | Arizona | Apache |
| UAZ | 38916 | Crotalus | oreganus | lutosus | USA | Arizona | Mohave |
| UAZ | 38917 | Crotalus | viridis | nuntius | USA | Arizona | Navajo |
| UAZ | 38918 | Crotalus | viridis | viridis | USA |  |  |
| UAZ | 39360 | Crotalus | viridis | viridis | USA | New Mexico |  |
| UAZ | 39486 | Crotalus | viridis | viridis | USA | New Mexico |  |
| UAZ | 39831 | Crotalus | viridis | nuntius | USA | Arizona | Apache |
| UAZ | 39832 | Crotalus | oreganus | abyssus | USA | Arizona | Coconino |
| UAZ | 39883 | Crotalus | oreganus | abyssus | USA | Arizona | Coconino |
| UAZ | 40086 | Crotalus | oreganus | cerberus | USA | Arizona | Navajo |
| UAZ | 40157 | Crotalus | oreganus | lutosus | USA | Arizona | Mohave |
| UAZ | 40162 | Crotalus | viridis |  | USA | Arizona |  |
| UAZ | 41537 | Crotalus | viridis | viridis | USA | Colorado |  |
| UAZ | 41538 | Crotalus | oreganus | oreganus | USA | Utah |  |
| UAZ | 41539 | Crotalus | oreganus | cerberus | USA | Arizona | Pima |
| UAZ | 41540 | Crotalus | oreganus | cerberus | USA | Arizona | Pima |
| UAZ | 41540 | Crotalus | oreganus | cerberus | USA | Arizona | Pima |
| UAZ | 41542 | Crotalus | viridis | nuntius | USA | Arizona | Apache |
| UAZ | 41970 | Crotalus | viridis | viridis | USA | Colorado |  |
| UAZ | 42589 | Crotalus | viridis | viridis | USA | New Mexico |  |
| UAZ | 43032 | Crotalus | viridis | viridis | USA | New Mexico |  |
| UAZ | 43292 | Crotalus | viridis | viridis | USA |  |  |
| UAZ | 44807 | Crotalus | viridis | nuntius | USA | Arizona | Apache |
| UAZ | 46405 |  |  |  |  |  |  |
| UAZ | 46406 |  |  |  |  |  |  |
| UAZ | 46573 | Crotalus | viridis | nuntius | USA | Arizona | Apache |
| UAZ | 47265 | Crotalus | oreganus | abyssus | USA | Arizona | Coconino |
| UAZ | 47267 | Crotalus | oreganus | cerberus | USA | Arizona | Pima |
| UAZ | 47268 | Crotalus | viridis | nuntius | USA | Arizona | Coconino |
| UAZ | 47269 | Crotalus | viridis | nuntius | USA | Arizona | Coconino |
| UAZ | 47270 | Crotalus | viridis | viridis | USA | New Mexico | Navajo |
| UAZ | 47838 | Crotalus | viridis | nuntius | USA | Arizona |  |
| UAZ | 47839 | Crotalus | viridis | nuntius | USA | Arizona | Navajo |
| UAZ | 48008 | Crotalus | oreganus | lutosus | USA | Arizona | Coconino |
| UAZ | 48014 | Crotalus | oreganus | lutosus | USA | Arizona |  |
| UAZ | 48014 | Crotalus | oreganus | lutosus | USA | Arizona |  |
| UAZ | 48251 | Crotalus | oreganus | cerberus | USA | Arizona | Gila |
| UAZ | 50292 | Crotalus | oreganus | nuntius | USA | Arizona | Coconino |
| UAZ | 50293 | Crotalus | oreganus | cerberus | USA | Arizona | Gila |
| UAZ | 50691 | Crotalus | viridis | viridis | USA | New Mexico |  |
| UAZ | 51059 | Crotalus | viridis | nuntius | USA | Arizona | Coconino |
| UAZ | 51060 | Crotalus | viridis | nuntius | USA | Arizona | Coconino |
| UAZ | 51940 | Crotalus | viridis | nuntius | USA | Arizona | Navajo |
| UAZ | 52114 | Crotalus | viridis | viridis | USA | New Mexico |  |
| UAZ | 52115 | Crotalus | viridis | nuntius | USA | Arizona | Coconino |
| UAZ | 52587 | Crotalus | viridis | nuntius | USA | Arizona |  |
| UAZ | 53409 | Crotalus | oreganus | cerberus | USA | Arizona | Yavapai |
| UAZ | 53535 | Crotalus | viridis | nuntius | USA | Arizona | Coconino |
| UAZ | 53577 | Crotalus | viridis | nuntius | USA | Arizona | Coconino |
| UAZ | 53578 | Crotalus | viridis | nuntius | USA | Arizona | Coconino |
| UAZ | 53626 | Crotalus | oreganus | lutosus | USA | Arizona | Coconino |
| UAZ | 55413 | Crotalus | oreganus | cerberus | USA | Arizona | Greenlee |
| UAZ | 55717 | Crotalus | viridis | nuntius | USA | Arizona |  |
| UAZ | 55731 | Crotalus | oreganus | lutosus | USA | Arizona |  |
| UAZ | 55732 | Crotalus | oreganus | lutosus | USA | Arizona |  |
| UAZ | 56300 | Crotalus | oreganus | cerberus | USA | Arizona | Pinal |
| UMNH | 6 | Crotalus | oreganus | lutosus | USA | Utah | Sanpete |
| UMNH | 92 | Crotalus | oreganus | lutosus | USA | Idaho | Bonneville |
| UMNH | 93 | Crotalus | oreganus | lutosus | USA | Idaho | Bonneville |
| UMNH | 94 | Crotalus | oreganus | lutosus | USA | Idaho | Bonneville |
| UMNH | 108 | Crotalus | oreganus | lutosus | USA | Idaho | Bonneville |
| UMNH | 111 | Crotalus | oreganus | lutosus | USA | Utah | Washington |
| UMNH | 288 | Crotalus | oreganus | lutosus | USA | Utah | Tooele |
| UMNH | 323 | Crotalus | oreganus | lutosus | USA | Utah | Washington |
| UMNH | 338 | Crotalus | oreganus | lutosus | USA | Utah | Washington |
| UMNH | 352 | Crotalus | oreganus | lutosus | USA | Utah | Salt Lake |
| UMNH | 635 | Crotalus | oreganus | lutosus | USA | Utah | Washington |
| UMNH | 676 | Crotalus | oreganus | lutosus | USA | Utah | Washington |
| UMNH | 680 | Crotalus | oreganus | lutosus | USA | Utah | Sanpete |
| UMNH | 681 | Crotalus | oreganus | lutosus | USA | Utah | Washington |
| UMNH | 691 | Crotalus | oreganus | oreganus | USA | Idaho |  |
| UMNH | 831 | Crotalus | viridis | nuntius | USA | Colorado | Moffat |
| UMNH | 832 | Crotalus | viridis | nuntius | USA | Colorado | Moffat |
| UMNH | 844 | Crotalus | unknown | unknown | USA |  |  |
| UMNH | 855 | Crotalus | oreganus | concolor | USA | Wyoming | Sweetwater |
| UMNH | 860 | Crotalus | viridis | viridis | USA | Colorado | Moffat |
| UMNH | 876 | Crotalus | oreganus | concolor | USA | Utah | San Juan |
| UMNH | 907 | Crotalus | oreganus | lutosus | USA | Utah | Box Elder |
| UMNH | 907 | Crotalus | oreganus | lutosus | USA | Utah | Box Elder |
| UMNH | 911 | Crotalus | oreganus | lutosus | USA | Utah | Box Elder |
| UMNH | 912 | Crotalus | oreganus | lutosus | USA | Utah | Box Elder |
| UMNH | 913 | Crotalus | oreganus | lutosus | USA | Utah | Box Elder |
| UMNH | 914 | Crotalus | oreganus | lutosus | USA | Utah | Box Elder |
| UMNH | 915 | Crotalus | oreganus | lutosus | USA | Utah | Box Elder |
| UMNH | 916 | Crotalus | oreganus | lutosus | USA | Utah | Box Elder |
| UMNH | 919 | Crotalus | oreganus | lutosus | USA | Utah | Box Elder |
| UMNH | 920 | Crotalus | oreganus | lutosus | USA | Utah | Box Elder |
| UMNH | 921 | Crotalus | oreganus | lutosus | USA | Utah | Box Elder |
| UMNH | 942 | Crotalus | oreganus | lutosus | USA | Utah | Box Elder |
| UMNH | 944 | Crotalus | unknown | unknown |  |  |  |
| UMNH | 962 | Crotalus | oreganus | concolor | USA | Utah | San Juan |
| UMNH | 987 | Crotalus | oreganus | lutosus | USA |  |  |
| UMNH | 1004 | Crotalus | viridis | viridis | USA | Colorado | Moffat |
| UMNH | 1023 | Crotalus | viridis | viridis | USA | Colorado | Moffat |
| UMNH | 1025 | Crotalus | viridis | viridis | USA | Colorado | Moffat |
| UMNH | 1026 | Crotalus | viridis | viridis | USA | Colorado | Moffat |
| UMNH | 1027 | Crotalus | viridis | viridis | USA | Colorado | Moffat |
| UMNH | 1045 | Crotalus | viridis | viridis | USA | Colorado | Moffat |
| UMNH | 1045 | Crotalus | viridis | viridis | USA | Colorado | Moffat |
| UMNH | 1051 | Crotalus | viridis | viridis | USA | Colorado | Moffat |
| UMNH | 1058 | Crotalus | oreganus | lutosus | USA | Nevada | Lander |
| UMNH | 1060 | Crotalus | oreganus | lutosus | USA | Nevada | Lander |
| UMNH | 1112 | Crotalus | viridis | viridis | USA | Montana | Phillips |
| UMNH | 1133 | Crotalus | unknown | unknown |  |  |  |
| UMNH | 1134 | Crotalus | viridis | nuntius | USA | Arizona | Navajo |
| UMNH | 1137 | Crotalus | viridis | nuntius | USA | Arizona | Navajo |
| UMNH | 1138 | Crotalus | unknown | unknown |  |  |  |
| UMNH | 1139 | Crotalus | unknown | unknown |  |  |  |
| UMNH | 1141 | Crotalus | unknown | unknown |  |  |  |
| UMNH | 1142 | Crotalus | unknown | unknown |  |  |  |
| UMNH | 1143 | Crotalus | unknown | unknown |  |  |  |
| UMNH | 1143 | Crotalus | unknown | unknown |  |  |  |
| UMNH | 1150 | Crotalus | unknown | unknown |  |  |  |
| UMNH | 1151 | Crotalus | unknown | unknown |  |  |  |
| UMNH | 1153 | Crotalus | unknown | unknown |  |  |  |
| UMNH | 1157 | Crotalus | oreganus | concolor | USA | Utah | San Juan |
| UMNH | 1159 | Crotalus | oreganus | concolor | USA | Utah | San Juan |
| UMNH | 1160 | Crotalus | oreganus | concolor | USA | Utah | San Juan |
| UMNH | 1160 | Crotalus | oreganus | concolor | USA | Utah | San Juan |
| UMNH | 1162 | Crotalus | oreganus | concolor | USA | Utah | San Juan |
| UMNH | 1197 | Crotalus | unknown | unknown |  |  |  |
| UMNH | 1198 | Crotalus | unknown | unknown |  |  |  |
| UMNH | 1199 | Crotalus | unknown | unknown |  |  |  |
| UMNH | 1200 | Crotalus | unknown | unknown |  |  |  |
| UMNH | 1201 | Crotalus | unknown | unknown |  |  |  |
| UMNH | 1207 | Crotalus | oreganus | lutosus | USA | Utah | Morgan |
| UMNH | 1208 | Crotalus | oreganus | lutosus | USA | Utah | Morgan |
| UMNH | 1261 | Crotalus | oreganus | concolor | USA | Arizona |  |
| UMNH | 1262 | Crotalus | viridis | nuntius | USA | Arizona | Navajo |
| UMNH | 1263 | Crotalus | viridis | nuntius | USA | Arizona | Navajo |
| UMNH | 1264 | Crotalus | viridis | nuntius | USA | Arizona | Navajo |
| UMNH | 1266 | Crotalus | viridis | nuntius | USA | Arizona | Navajo |
| UMNH | 1268 | Crotalus | oreganus | lutosus | USA | Utah | Washington |
| UMNH | 1268 | Crotalus | oreganus | lutosus | USA | Utah | Washington |
| UMNH | 1269 | Crotalus | oreganus | lutosus | USA | Utah | Washington |
| UMNH | 1270 | Crotalus | oreganus | lutosus | USA | Utah | Washington |
| UMNH | 1271 | Crotalus | oreganus | lutosus | USA | Utah | Washington |
| UMNH | 1294 | Crotalus | oreganus | lutosus | USA | Utah | Utah |
| UMNH | 1296 | Crotalus | oreganus | lutosus | USA | Utah | Utah |
| UMNH | 1303 | Crotalus | oreganus | lutosus | USA | Utah | Salt Lake |
| UMNH | 1304 | Crotalus | oreganus | lutosus | USA | Utah | Carbon |
| UMNH | 1311 | Crotalus | unknown | unknown |  |  |  |
| UMNH | 1359 | Crotalus | oreganus | lutosus | USA | Utah | Carbon |
| UMNH | 1432 | Crotalus | oreganus | lutosus | USA | Utah | Tooele |
| UMNH | 1433 | Crotalus | oreganus | lutosus | USA | Utah | Tooele |
| UMNH | 1626 | Crotalus | viridis | viridis | USA | Arizona | Navajo |
| UMNH | 1930 | Crotalus | viridis | nuntius | USA | Arizona |  |
| UMNH | 1944 | Crotalus | oreganus | lutosus | USA | Utah | Summit |
| UMNH | 1971 | Crotalus | oreganus | lutosus | USA | Utah | Tooele |
| UMNH | 1972 | Crotalus | oreganus | lutosus | USA | Utah | Tooele |
| UMNH | 1973 | Crotalus | oreganus | lutosus | USA | Utah | Tooele |
| UMNH | 1974 | Crotalus | oreganus | lutosus | USA | Utah | Summit |
| UMNH | 1976 | Crotalus | oreganus | lutosus | USA | Utah | Morgan |
| UMNH | 1977 | Crotalus | oreganus | lutosus | USA | Utah | Tooele |
| UMNH | 1977 | Crotalus | oreganus | lutosus | USA | Utah | Tooele |
| UMNH | 1979 | Crotalus | oreganus | lutosus | USA | Utah | Tooele |
| UMNH | 1989 | Crotalus | oreganus | lutosus | USA | Utah | Tooele |
| UMNH | 1992 | Crotalus | oreganus | lutosus | USA | Utah | Tooele |
| UMNH | 1993 | Crotalus | oreganus | lutosus | USA | Utah | Tooele |
| UMNH | 1994 | Crotalus | oreganus | lutosus | USA | Utah | Summit |
| UMNH | 1995 | Crotalus | oreganus | lutosus | USA | Utah | Tooele |
| UMNH | 2017 | Crotalus | oreganus | lutosus | USA | Utah | Summit |
| UMNH | 2022 | Crotalus | oreganus | lutosus | USA | Utah | Tooele |
| UMNH | 2105 | Crotalus | oreganus | lutosus | USA | Utah | Summit |
| UMNH | 2106 | Crotalus | oreganus | lutosus | USA | Utah | Summit |
| UMNH | 2106 | Crotalus | oreganus | lutosus | USA | Utah | Summit |
| UMNH | 2114 | Crotalus | oreganus | lutosus | USA | Utah | Tooele |
| UMNH | 2195 | Crotalus | oreganus | lutosus | USA | Utah | Summit |
| UMNH | 2196 | Crotalus | oreganus | lutosus | USA | Utah | Summit |
| UMNH | 2197 | Crotalus | oreganus | lutosus | USA | Utah | Summit |
| UMNH | 2199 | Crotalus | oreganus | lutosus | USA | Utah | Summit |
| UMNH | 2200 | Crotalus | oreganus | lutosus | USA | Utah | Summit |
| UMNH | 2201 | Crotalus | oreganus | lutosus | USA | Utah | Summit |
| UMNH | 2202 | Crotalus | oreganus | lutosus | USA | Utah | Summit |
| UMNH | 2203 | Crotalus | oreganus | lutosus | USA | Utah | Summit |
| UMNH | 2204 | Crotalus | oreganus | lutosus | USA | Utah | Summit |
| UMNH | 2205 | Crotalus | oreganus | lutosus | USA | Utah | Summit |
| UMNH | 2206 | Crotalus | oreganus | lutosus | USA | Utah | Summit |
| UMNH | 2207 | Crotalus | oreganus | lutosus | USA | Utah | Summit |
| UMNH | 2208 | Crotalus | oreganus | lutosus | USA | Utah | Summit |
| UMNH | 2209 | Crotalus | oreganus | lutosus | USA | Utah | Summit |
| UMNH | 2217 | Crotalus | oreganus | lutosus | USA | Utah | Summit |
| UMNH | 2218 | Crotalus | oreganus | lutosus | USA | Utah | Summit |
| UMNH | 2219 | Crotalus | oreganus | lutosus | USA | Utah | Summit |
| UMNH | 2220 | Crotalus | oreganus | lutosus | USA | Utah | Summit |
| UMNH | 2221 | Crotalus | oreganus | lutosus | USA | Utah | Summit |
| UMNH | 2222 | Crotalus | oreganus | lutosus | USA | Utah | Summit |
| UMNH | 2223 | Crotalus | oreganus | lutosus | USA | Utah | Morgan |
| UMNH | 2224 | Crotalus | oreganus | lutosus | USA | Utah | Morgan |
| UMNH | 2226 | Crotalus | oreganus | lutosus | USA | Utah | Morgan |
| UMNH | 2228 | Crotalus | oreganus | lutosus | USA | Utah | Morgan |
| UMNH | 2229 | Crotalus | oreganus | lutosus | USA | Utah | Morgan |
| UMNH | 2230 | Crotalus | oreganus | lutosus | USA | Utah | Morgan |
| UMNH | 2231 | Crotalus | oreganus | lutosus | USA | Utah | Morgan |
| UMNH | 2237 | Crotalus | oreganus | lutosus | USA | Utah | Tooele |
| UMNH | 2357 | Crotalus | oreganus | concolor | USA | Utah | Carbon |
| UMNH | 2360 | Crotalus | viridis | nuntius | USA | Arizona | Navajo |
| UMNH | 2361 | Crotalus | viridis | nuntius | USA | Arizona | Navajo |
| UMNH | 2408 | Crotalus | oreganus | lutosus | USA | Utah | Summit |
| UMNH | 2480 | Crotalus | oreganus | lutosus | USA | Utah | Utah |
| UMNH | 2494 | Crotalus | oreganus | lutosus | USA | Utah | Tooele |
| UMNH | 2807 | Crotalus | oreganus | lutosus | USA | Utah | Millard |
| UMNH | 2825 | Crotalus | oreganus | lutosus | USA | Utah |  |
| UMNH | 2833 | Crotalus | unknown | unknown |  |  |  |
| UMNH | 2852 | Crotalus | unknown | unknown |  |  |  |
| UMNH | 2854 | Crotalus | oreganus | concolor | USA | Utah | Salt Lake |
| UMNH | 2858 | Crotalus | oreganus | concolor | USA | Utah | San Juan |
| UMNH | 2859 | Crotalus | oreganus | concolor | USA | Utah | San Juan |
| UMNH | 2868 | Crotalus | oreganus | concolor | USA | Utah | Unitah |
| UMNH | 2875 | Crotalus | oreganus | concolor | USA | Utah | San Juan |
| UMNH | 2877 | Crotalus | oreganus | concolor | USA | Utah | San Juan |
| UMNH | 2878 | Crotalus | oreganus | concolor | USA | Utah | San Juan |
| UMNH | 2878 | Crotalus | oreganus | concolor | USA | Utah | San Juan |
| UMNH | 2879 | Crotalus | oreganus | concolor | USA | Utah | San Juan |
| UMNH | 2880 | Crotalus | oreganus | concolor | USA | Utah | San Juan |
| UMNH | 2881 | Crotalus | oreganus | concolor | USA | Utah | San Juan |
| UMNH | 2882 | Crotalus | oreganus | concolor | USA | Utah | San Juan |
| UMNH | 2883 | Crotalus | oreganus | concolor | USA | Utah | San Juan |
| UMNH | 2884 | Crotalus | oreganus | concolor | USA | Utah | San Juan |
| UMNH | 3117 | Crotalus | oreganus | lutosus | USA | Utah | Utah |
| UMNH | 3249 | Crotalus | oreganus | lutosus | USA | Utah | Box Elder |
| UMNH | 3288 | Crotalus | oreganus | lutosus | USA | Utah | Kane |
| UMNH | 3321 | Crotalus | oreganus | concolor | USA | Utah |  |
| UMNH | 3323 | Crotalus | oreganus | concolor | USA | Utah |  |
| UMNH | 3342 | Crotalus | oreganus | lutosus | USA | Utah | Salt Lake |
| UMNH | 3378 | Crotalus | viridis | viridis | USA | Utah | San Juan |
| UMNH | 3548 | Crotalus | oreganus | concolor | USA | Wyoming | Sweetwater |
| UMNH | 3549 | Crotalus | oreganus | concolor | USA | Wyoming | Sweetwater |
| UMNH | 3550 | Crotalus | oreganus | concolor | USA | Wyoming | Sweetwater |
| UMNH | 3551 | Crotalus | oreganus | concolor | USA | Wyoming | Sweetwater |
| UMNH | 3552 | Crotalus | oreganus | concolor | USA | Wyoming | Sweetwater |
| UMNH | 3553 | Crotalus | oreganus | concolor | USA | Wyoming | Sweetwater |
| UMNH | 3751 | Crotalus | viridis | viridis | USA | Colorado | Archuleta |
| UMNH | 3752 | Crotalus | viridis | viridis | USA | Colorado | Archuleta |
| UMNH | 3753 | Crotalus | viridis | viridis | USA | Colorado | Archuleta |
| UMNH | 8023 | Crotalus | oreganus | lutosus | USA | Nevada | White Pine |
| UMNH | 8031 | Crotalus | unknown | unknown |  |  |  |
| UMNH | 8034 | Crotalus | unknown | unknown |  |  |  |
| UMNH | 8035 | Crotalus | unknown | unknown |  |  |  |
| UMNH | 8036 | Crotalus | unknown | unknown |  |  |  |
| UMNH | 8036 | Crotalus | unknown | unknown |  |  |  |
| UMNH | 8041 | Crotalus | unknown | unknown |  |  |  |
| UMNH | 8165 | Crotalus | oreganus | lutosus | USA | Utah | Washington |
