# Supplementary material for: Deconstructing a Species-Complex: Geometric Morphometric and Molecular Analyses Define Species in the Western Rattlesnake (Crotalus viridis)
Source: PLoS One. 2016 Jan 27;11(1):e0146166. doi: 10.1371/journal.pone.0146166 (PMC4731396; doi:10.1371/journal.pone.0146166)
Supplement: S2 Table — List of mitochondrial (mt) DNA sequences accessed in this study. A total of 117 Western Rattlesnake sequences, representing six mtDNA regions, were accessed and subsequently concatenated by subspecies, with these data yielding a Bayesian Inference phylogenetic hypothesis. (DOCX) [file pone.0146166.s003.docx]

**S3. Sequence Accession**

List of mitochondrial (mt) DNA sequences accessed in this study. A total of 117 sequences from 5 mtDNA regions were accessed to construct concatenated sequences for Western Rattlesnake subspecies and to derive single-region Bayesian Inference phylogenetic hypotheses.

| Accession Number | Genus | species | subspecies | mtDNA Region | Authors |
| --- | --- | --- | --- | --- | --- |
| AY016037 | *Crotalus* | *viridis* | *abyssus* | D-Loop | Ashton and de Queiroz (2001) |
| AY016038 | *Crotalus* | *viridis* | *caliginis* | D-Loop | Ashton and de Queiroz (2001) |
| AY016042 | *Crotalus* | *viridis* | *concolor* | D-Loop | Ashton and de Queiroz (2001) |
| AY016032 | *Crotalus* | *viridis* | *helleri* | D-Loop | Ashton and de Queiroz (2001) |
| AY016229 | *Crotalus* | *viridis* | *helleri* | D-Loop | Ashton and de Queiroz (2001) |
| AY016040 | *Crotalus* | *viridis* | *lutosus* | D-Loop | Ashton and de Queiroz (2001) |
| AY016026 | *Crotalus* | *viridis* | *lutosus* | D-Loop | Ashton and de Queiroz (2001) |
| AY016033 | *Crotalus* | *viridis* | *lutosus* | D-Loop | Ashton and de Queiroz (2001) |
| AY016036 | *Crotalus* | *viridis* | *lutosus* | D-Loop | Ashton and de Queiroz (2001) |
| AY016218 | *Crotalus* | *viridis* | *nuntius* | D-Loop | Ashton and de Queiroz (2001) |
| AY016027 | *Crotalus* | *viridis* | *oreganus* | D-Loop | Ashton and de Queiroz (2001) |
| AY016031 | *Crotalus* | *viridis* | *oreganus* | D-Loop | Ashton and de Queiroz (2001) |
| AY016034 | *Crotalus* | *viridis* | *oreganus* | D-Loop | Ashton and de Queiroz (2001) |
| AY016046 | *Crotalus* | *viridis* | *viridis* | D-Loop | Ashton and de Queiroz (2001) |
| AY016029 | *Crotalus* | *viridis* | *viridis* | D-Loop | Ashton and de Queiroz (2001) |
| AY016030 | *Crotalus* | *viridis* | *viridis* | D-Loop | Ashton and de Queiroz (2001) |
| AY016045 | *Crotalus* | *viridis* | *viridis* | D-Loop | Ashton and de Queiroz (2001) |
| AY016044 | *Crotalus* | *viridis* | *viridis* | D-Loop | Ashton and de Queiroz (2001) |
| AY016049 | *Crotalus* | *atrox* |  | D-Loop | Ashton and de Queiroz (2001) |
| AY016051 | *Crotalus* | *mitchelli* |  | D-Loop | Ashton and de Queiroz (2001) |
| AY016048 | *Crotalus* | *scutulatus* |  | D-Loop | Ashton and de Queiroz (2001) |
| AY016047 | *Crotalus* | *scutulatus* |  | D-Loop | Ashton and de Queiroz (2001) |
| AY016050 | *Crotalus* | *tigris* |  | D-Loop | Ashton and de Queiroz (2001) |
| AY016227 | *Crotalus* | *viridis* | *abyssus* | ND2 | Ashton and de Queiroz (2001) |
| AY016228 | *Crotalus* | *viridis* | *caliginis* | ND2 | Ashton and de Queiroz (2001) |
| AY016225 | *Crotalus* | *viridis* | *cerberus* | ND2 | Ashton and de Queiroz (2001) |
| AY016232 | *Crotalus* | *viridis* | *concolor* | ND2 | Ashton and de Queiroz (2001) |
| AY016231 | *Crotalus* | *viridis* | *concolor* | ND2 | Ashton and de Queiroz (2001) |
| AY016233 | *Crotalus* | *viridis* | *concolor* | ND2 | Ashton and de Queiroz (2001) |
| AY016222 | *Crotalus* | *viridis* | *helleri* | ND2 | Ashton and de Queiroz (2001) |
| AY016229 | *Crotalus* | *viridis* | *helleri* | ND2 | Ashton and de Queiroz (2001) |
| AY016244 | *Crotalus* | *viridis* | *lutosus* | ND2 | Ashton and de Queiroz (2001) |
| AY016215 | *Crotalus* | *viridis* | *lutosus* | ND2 | Ashton and de Queiroz (2001) |
| AY016223 | *Crotalus* | *viridis* | *lutosus* | ND2 | Ashton and de Queiroz (2001) |
| AY016230 | *Crotalus* | *viridis* | *lutosus* | ND2 | Ashton and de Queiroz (2001) |
| AY016226 | *Crotalus* | *viridis* | *lutosus* | ND2 | Ashton and de Queiroz (2001) |
| AY016218 | *Crotalus* | *viridis* | *nuntius* | ND2 | Ashton and de Queiroz (2001) |
| AY016217 | *Crotalus* | *viridis* | *oreganus* | ND2 | Ashton and de Queiroz (2001) |
| AY016221 | *Crotalus* | *viridis* | *oreganus* | ND2 | Ashton and de Queiroz (2001) |
| AY016224 | *Crotalus* | *viridis* | *oreganus* | ND2 | Ashton and de Queiroz (2001) |
| AY016216 | *Crotalus* | *viridis* | *viridis* | ND2 | Ashton and de Queiroz (2001) |
| AY016243 | *Crotalus* | *viridis* | *viridis* | ND2 | Ashton and de Queiroz (2001) |
| AY016219 | *Crotalus* | *viridis* | *viridis* | ND2 | Ashton and de Queiroz (2001) |
| AY016242 | *Crotalus* | *viridis* | *viridis* | ND2 | Ashton and de Queiroz (2001) |
| AY016220 | *Crotalus* | *viridis* | *viridis* | ND2 | Ashton and de Queiroz (2001) |
| AY704799 | *Crotalus* | *viridis* | *viridis* | ND2 | Ashton and de Queiroz (2001) |
| AY016236 | *Crotalus* | *viridis* | *viridis* | ND2 | Ashton and de Queiroz (2001) |
| AY016235 | *Crotalus* | *viridis* | *viridis* | ND2 | Ashton and de Queiroz (2001) |
| AY016234 | *Crotalus* | *viridis* | *viridis* | ND2 | Ashton and de Queiroz (2001) |
| AY016239 | *Crotalus* | *atrox* |  | ND2 | Ashton and de Queiroz (2001) |
| AY016245 | *Crotalus* | *cerastes* |  | ND2 | Ashton and de Queiroz (2001) |
| AY016246 | *Crotalus* | *enyo* |  | ND2 | Ashton and de Queiroz (2001) |
| AY016241 | *Crotalus* | *mitchelli* |  | ND2 | Ashton and de Queiroz (2001) |
| AY016237 | *Crotalus* | *scutulatus* |  | ND2 | Ashton and de Queiroz (2001) |
| AY016238 | *Crotalus* | *scutulatus* |  | ND2 | Ashton and de Queiroz (2001) |
| AY016240 | *Crotalus* | *tigris* |  | ND2 | Ashton and de Queiroz (2001) |
| HQ257746 | *Crotalus* | *triseriatus* | *armstrongi* | ATPase 6&8 | Bryson et al. (20110 |
| CVU65377 | *Crotalus* | *viridis* | *viridis* | Cytochrome B | Cullings et al. (1996) |
| AF462374 | *Crotalus* | *viridis* | *cerberus* | ATPase 6&8 | Douglas et al. (2002) |
| AF462371 | *Crotalus* | *viridis* | *nuntius* | ATPase 6&8 | Douglas et al. (2002) |
| AF462367 | *Crotalus* | *viridis* | *viridis* | ATPase 6&8 | Douglas et al. (2002) |
| AF462368 | *Crotalus* | *viridis* | *viridis* | ATPase 6&8 | Douglas et al. (2002) |
| AF462369 | *Crotalus* | *viridis* | *viridis* | ATPase 6&8 | Douglas et al. (2002) |
| AF462370 | *Crotalus* | *viridis* | *viridis* | ATPase 6&8 | Douglas et al. (2002) |
| AF462360 | *Crotalus* | *viridis* | *concolor* | ATPase 6&8 | Douglas et al. (2002) |
| AF462361 | *Crotalus* | *viridis* | *concolor* | ATPase 6&8 | Douglas et al. (2002) |
| AF462375 | *Crotalus* | *viridis* | *helleri* | ATPase 6&8 | Douglas et al. (2002) |
| AF462364 | *Crotalus* | *viridis* | *lutosus* | ATPase 6&8 | Douglas et al. (2002) |
| AF462365 | *Crotalus* | *viridis* | *lutosus* | ATPase 6&8 | Douglas et al. (2002) |
| AF462366 | *Crotalus* | *viridis* | *lutosus* | ATPase 6&8 | Douglas et al. (2002) |
| AF462372 | *Crotalus* | *viridis* | *oreganus* | ATPase 6&8 | Douglas et al. (2002) |
| AF462373 | *Crotalus* | *viridis* | *oreganus* | ATPase 6&8 | Douglas et al. (2002) |
| AF462362 | *Crotalus* | *viridis* | *abyssus* | ATPase 6&8 | Douglas et al. (2002) |
| AF462363 | *Crotalus* | *viridis* | *abyssus* | ATPase 6&8 | Douglas et al. (2002) |
| AF147857 | *Crotalus* | *viridis* | *abyssus* | Cytochrome B | Pook et al. (2000) |
| AF147858 | *Crotalus* | *viridis* | *caliginis* | Cytochrome B | Pook et al. (2000) |
| AF147859 | *Crotalus* | *viridis* | *cerberus* | Cytochrome B | Pook et al. (2000) |
| AF147860 | *Crotalus* | *viridis* | *concolor* | Cytochrome B | Pook et al. (2000) |
| AF147863 | *Crotalus* | *viridis* | *helleri* | Cytochrome B | Pook et al. (2000) |
| AF147862 | *Crotalus* | *viridis* | *helleri* | Cytochrome B | Pook et al. (2000) |
| AF147861 | *Crotalus* | *viridis* | *helleri* | Cytochrome B | Pook et al. (2000) |
| AF147864 | *Crotalus* | *viridis* | *lutosus* | Cytochrome B | Pook et al. (2000) |
| AF147865 | *Crotalus* | *viridis* | *lutosus* | Cytochrome B | Pook et al. (2000) |
| AF147875 | *Crotalus* | *viridis* | *nuntius* | Cytochrome B | Pook et al. (2000) |
| AF147871 | *Crotalus* | *viridis* | *oreganus* | Cytochrome B | Pook et al. (2000) |
| AF147872 | *Crotalus* | *viridis* | *oreganus* | Cytochrome B | Pook et al. (2000) |
| AF147870 | *Crotalus* | *viridis* | *oreganus* | Cytochrome B | Pook et al. (2000) |
| AF147874 | *Crotalus* | *viridis* | *oreganus* | Cytochrome B | Pook et al. (2000) |
| AF147873 | *Crotalus* | *viridis* | *oreganus* | Cytochrome B | Pook et al. (2000) |
| AF471066 | *Crotalus* | *viridis* | *viridis* | Cytochrome B | Pook et al. (2000) |
| AF147866 | *Crotalus* | *viridis* | *viridis* | Cytochrome B | Pook et al. (2000) |
| AF147869 | *Crotalus* | *viridis* | *viridis* | Cytochrome B | Pook et al. (2000) |
| AF147868 | *Crotalus* | *viridis* | *viridis* | Cytochrome B | Pook et al. (2000) |
| AF147867 | *Crotalus* | *viridis* | *viridis* | Cytochrome B | Pook et al. (2000) |
| AF147876 | *Crotalus* | *scutulatus* |  | Cytochrome B | Pook et al. (2000) |
| AF194148 | *Crotalus* | *viridis* | *abyssus* | ND4 | Pook et al. (2000) |
| AF194149 | *Crotalus* | *viridis* | *caliginis* | ND4 | Pook et al. (2000) |
| AF194150 | *Crotalus* | *viridis* | *cerberus* | ND4 | Pook et al. (2000) |
| AF194151 | *Crotalus* | *viridis* | *concolor* | ND4 | Pook et al. (2000) |
| CVU41882 | *Crotalus* | *viridis* | *concolor* | ND4 | Pook et al. (2000) |
| AF194154 | *Crotalus* | *viridis* | *helleri* | ND4 | Pook et al. (2000) |
| AF194153 | *Crotalus* | *viridis* | *helleri* | ND4 | Pook et al. (2000) |
| AF194152 | *Crotalus* | *viridis* | *helleri* | ND4 | Pook et al. (2000) |
| AF194155 | *Crotalus* | *viridis* | *lutosus* | ND4 | Pook et al. (2000) |
| AF194156 | *Crotalus* | *viridis* | *lutosus* | ND4 | Pook et al. (2000) |
| AF194166 | *Crotalus* | *viridis* | *nuntius* | ND4 | Pook et al. (2000) |
| AF194162 | *Crotalus* | *viridis* | *oreganus* | ND4 | Pook et al. (2000) |
| AF194163 | *Crotalus* | *viridis* | *oreganus* | ND4 | Pook et al. (2000) |
| AF194161 | *Crotalus* | *viridis* | *oreganus* | ND4 | Pook et al. (2000) |
| AF194165 | *Crotalus* | *viridis* | *oreganus* | ND4 | Pook et al. (2000) |
| AF194164 | *Crotalus* | *viridis* | *oreganus* | ND4 | Pook et al. (2000) |
| AF194157 | *Crotalus* | *viridis* | *viridis* | ND4 | Pook et al. (2000) |
| AF194160 | *Crotalus* | *viridis* | *viridis* | ND4 | Pook et al. (2000) |
| AF194159 | *Crotalus* | *viridis* | *viridis* | ND4 | Pook et al. (2000) |
| AF194158 | *Crotalus* | *viridis* | *viridis* | ND4 | Pook et al. (2000) |
| AF194168 | *Crotalus* | *durissus* |  | ND4 | Pook et al. (2000) |
| AF194167 | *Crotalus* | *scutulatus* |  | ND4 | Pook et al. (2000) |
