# Supplementary material for: Deconstructing a Species-Complex: Geometric Morphometric and Molecular Analyses Define Species in the Western Rattlesnake (Crotalus viridis)
Source: PLoS One. 2016 Jan 27;11(1):e0146166. doi: 10.1371/journal.pone.0146166 (PMC4731396; doi:10.1371/journal.pone.0146166)
Supplement: S1 Text — We created a phylogenetic hypothesis based on 6 concatenated mtDNA regions acquired from GenBank (S2 Table: ATPase 8&6 (ATP8&6: TN+G); cytochrome B (Cyt-b: HKY + G); Displacement Loop (D-loop: HKY+G+I); NADH dehydrogenase subunit 2 (ND2: TIM (Transition Model)+G); and NADH dehydrogenase subunit 4L (ND4L: TN93+G+I). We merged sequences using the concatenation function in Geneious R8 [37] and arrayed them according to their presence in the mitochondrial genome, moving clockwise from D-loop (at 12 o’clock). The final arrangement was: D-loop—Cyt-b—ND4L—ATP8—ATP6—ND2, with sequences aligned using default settings in MUSCLE v 3.8.31 [38] (e.g. Gap Opening Cost = 15, Gap Extending Cost = 7). The model of nucleotide substitution for each mtDNA region was determined using ModelTest v 3.7 [39], with the concatenated sequences imported and partitioned into Mr. Bayes 3.2.5 [40–42], and the respective model of evolution applied to each partition. The Western Rattlesnake phylogeny was then estimated using the following conditions: heated chains = 4 (temp = 0.2); run simultaneously for 5,100,00 iterations; random seed = 16,288; burn-in = 1,000,000 iterations; subsampling frequency = 5,000 iterations; results = 4,000 trees, [i.e., ((5,100,000–1,000,000) x 4 / 5,000)]; branch lengths = unconstrained. Convergence was achieved within the first 250,000 iterations, autocorrelation was negligible within 1,024 samples, and log-likelihood plot indicated sufficient mixing. The resulting phylogeny was used for analyses requiring a phylogenetic comparative approach. (DOCX) [file pone.0146166.s004.docx]

**Supplemental Information**

1. **Analytical details for Model Selection**

When comparing multiple models to determine which combination of explanatory factors is appropriate for a matrix of dependent variables, some form of penalized log likelihood for models is calculated, such as Aikaike’s information criterion (AIC, Burnham and Anderson, 2002). For multivariate data, AIC is computed as

,

where *p* is the number of dependent variables, *k* is the number of model parameters, and is the *k* × *p* matrix of estimated coefficients for the model [1]. The model likelihood, is typically the determinant of the error covariance matrix of the model, , which is estimated as,

,

where **X** is the *n* × *k* model design matrix for the *n* subjects, and **^t^** means a matrix transpose. The matrix, **E**, is *n* × *p* matrix of residuals and the matrix product, **S**, is the matrix of sums of squares and cross-products for the model error. Therefore, the formula for AIC can be rewritten as

.

It can be inferred from this formula that a good model is one that has low error in spite of the number of parameters used. The first part of the formula is the model log-likelihood; the second part of the formula is the parameter penalty. A parsimonious model is one that produces the lowest AIC from a set of candidate models. However, with high-dimensional data (large *p*), when is not full rank (which is especially the case with landmark data containing many semi-landmarks), might be singular, rendering AIC calculations impossible. An alternative is to use the trace of . Furthermore, the trace of is equal to the sum of eigenvalues from a singular value decomposition of . Performing a singular value decomposition is helpful because the number of positive eigenvalues determines the rank of . Thus, one could assign *p’* equal to the number of positive eigenvalues as the number of relevant variables, which can be substituted into the AIC equation above, as well as replacing with the sum of positive eigenvalues. This is not a panacea, however, as different models might produce different numbers of *p’* variables, and the statistical issues with such a method have not been explored.

Rather than force model likelihoods into a computation of AIC, we simply calculated the log likelihood of models as

,

where $\Lambda'$ is reduced form of the *p* × *p* diagonal matrix of eigenvalues, $\Lambda$, found from the singular value decomposition of , such that

,

where **P** is the set of eigenvectors produced. By plotting the log of $trace\left(\Lambda'\right)()$ calculated this way versus the log of parameter penalties (as obtained from the second half of the AIC equation), one can find the parsimonious model as the most negative residuals from a linear fit (i.e., the model that produces the least error compared to expected from the number of parameters). The residuals function as a model selection index (MSI) for comparing candidate models. (Note that calculation by -2 is an unnecessary linear transformation for this method. Doing so would produce the same residual values.)

We calculated the trace of the relevant dimensions of model error covariance matrix and parameter penalties of 40 different models combining head size (calculated as the log of centroid size of the dorsal landmark configuration) as a covariate; developmental stage (juvenile, adult), sex, and either species or subspecies as factors; plus, all factor-covariate interactions. These 40 models are indexed in Table S1, and a graphical representation of the results is shown in Fig. S1.

Table S1. Information for a method of model selection. Models are indexed (see Fig. S1) and contain effects including CS = log(*CS*), ST = developmental stage, SEX = sex, SU = subspecies, and S = species, plus interactions. Model rank is the same as the number of model parameters, $trace\left(\Lambda'\right)()$ is the trace of the relevant dimensions of the error covariance matrix, the parameter penalty is equal to, where $p'$ is the number of relevant dimensions, and the model selection index (MSI) is the residual $trace\left(\Lambda'\right)()$ after regression against the parameter penalty. By subtracting the minimum value of MSI from all MSI, ΔMSI provided a positive departure from the best model for all models. The gray cells highlight the best model.

| Index | Model | Model rank |  | Parameter penalty |  | MSI | ΔMSI |
| --- | --- | --- | --- | --- | --- | --- | --- |
| 1 | Null | 1 | 0.0028 | 10300 | 100 | 0.0412 | 0.0705 |
| 2 | CS | 2 | 0.0026 | 10706 | 101 | -0.0086 | 0.0206 |
| 3 | ST | 4 | 0.0027 | 11110 | 101 | 0.0145 | 0.0438 |
| 4 | SEX | 2 | 0.0028 | 10500 | 100 | 0.0432 | 0.0724 |
| 5 | SU | 9 | 0.0027 | 12120 | 101 | 0.0212 | 0.0505 |
| 6 | S | 2 | 0.0027 | 10500 | 100 | 0.0272 | 0.0565 |
| 7 | CS, ST | 5 | 0.0026 | 11526 | 102 | -0.0008 | 0.0284 |
| 8 | CS, SEX | 3 | 0.0026 | 10908 | 101 | -0.0067 | 0.0226 |
| 9 | CS, SU | 10 | 0.0025 | 12546 | 102 | -0.0293 | 0.0000 |
| 10 | CS, S | 3 | 0.0026 | 11118 | 102 | -0.0197 | 0.0096 |
| 11 | ST, SEX | 5 | 0.0027 | 11312 | 101 | 0.0165 | 0.0458 |
| 12 | ST, SU | 12 | 0.0026 | 12954 | 102 | -0.0104 | 0.0189 |
| 13 | SEX, SU | 5 | 0.0026 | 11312 | 101 | -0.0011 | 0.0282 |
| 14 | ST, S | 10 | 0.0027 | 12322 | 101 | 0.0229 | 0.0521 |
| 15 | SEX, S | 3 | 0.0027 | 10700 | 100 | 0.0293 | 0.0585 |
| 16 | CS, ST, SEX | 6 | 0.0026 | 11730 | 102 | 0.0009 | 0.0302 |
| 17 | CS, ST, SU | 13 | 0.0025 | 13158 | 102 | -0.0245 | 0.0047 |
| 18 | CS, SEX, SU | 6 | 0.0026 | 11730 | 102 | -0.0155 | 0.0138 |
| 19 | CS, ST, S | 11 | 0.0025 | 12750 | 102 | -0.0278 | 0.0014 |
| 20 | CS, SEX, S | 4 | 0.0026 | 11322 | 102 | -0.0178 | 0.0115 |
| 21 | ST, SEX, SU | 13 | 0.0026 | 13158 | 102 | -0.0087 | 0.0205 |
| 22 | CS, ST, SEX, SU | 6 | 0.0026 | 11514 | 101 | 0.0010 | 0.0303 |
| 23 | ST, SEX, S | 14 | 0.0025 | 13362 | 102 | -0.0232 | 0.0061 |
| 24 | CS, ST, SEX, S | 7 | 0.0026 | 11934 | 102 | -0.0137 | 0.0156 |
| 25 | CS, ST, CS×ST | 7 | 0.0026 | 11934 | 102 | 0.0010 | 0.0303 |
| 26 | CS, SEX, CS×SEX | 4 | 0.0026 | 11110 | 101 | -0.0043 | 0.0250 |
| 27 | CS, SU, CS×SU | 18 | 0.0025 | 14178 | 102 | -0.0167 | 0.0126 |
| 28 | CS, S, CS×S | 4 | 0.0026 | 11322 | 102 | -0.0184 | 0.0109 |
| 29 | CS, ST×SEX | 8 | 0.0026 | 12138 | 102 | 0.0059 | 0.0351 |
| 30 | CS, ST×SU | 29 | 0.0025 | 16422 | 102 | 0.0019 | 0.0311 |
| 31 | CS, ST×S | 8 | 0.0026 | 12138 | 102 | -0.0120 | 0.0172 |
| 32 | CS, SEX×SU | 19 | 0.0025 | 14382 | 102 | -0.0115 | 0.0178 |
| 33 | CS, SEX×S | 5 | 0.0026 | 11526 | 102 | -0.0154 | 0.0139 |
| 34 | CS, ST×SEX, CS×SEX, CS×ST | 13 | 0.0026 | 13158 | 102 | 0.0142 | 0.0435 |
| 35 | CS, ST×SU, CS×SU, CS×ST | 53 | 0.0024 | 21318 | 102 | 0.0291 | 0.0584 |
| 36 | CS, ST×S, CS×S, CS×ST | 13 | 0.0026 | 13158 | 102 | -0.0056 | 0.0237 |
| 37 | CS, SU×SEX, CS×SU, CS×SEX | 36 | 0.0025 | 17850 | 102 | 0.0132 | 0.0424 |
| 38 | CS, S×SEX, CS×S, CS×SEX | 8 | 0.0026 | 12138 | 102 | -0.0096 | 0.0197 |
| 39 | CS, SU×ST, CS×SU, CS×ST | 28 | 0.0025 | 16218 | 102 | 0.0158 | 0.0451 |
| 40 | CS, S×ST, CS×S, CS×ST | 7 | 0.0026 | 11716 | 101 | 0.0021 | 0.0314 |

Figure S1. Graphical evidence of model selection. In the left plot, a log-linear fit is shown. The log(error) is the log of $trace\left(\Lambda'\right)()$ and the log(penalty) is the log of , as described in Table S1. The plot on the right shows the residuals from the plot on the left in the order of models as indexed in Table S1. The red value indicates the best model.

Reference

1. Bedrick EJ, Tsai CL. Model selection for multivariate regression in small samples. Biometrics. 1994;50(1):226-31. doi: 10.2307/2533213. PubMed PMID: WOS:A1994NH26500022.
